# Reinvestigation of the Deceptively Simple Reaction of Toluene with OH and the Fate of the Benzyl Radical: The “Hidden” Routes to Cresols and Benzaldehyde

**DOI:** 10.1021/acs.jpca.0c03727

**Published:** 2020-06-16

**Authors:** Zoi Salta, Agnie M. Kosmas, Marc E. Segovia, Martina Kieninger, Nicola Tasinato, Vincenzo Barone, Oscar N. Ventura

**Affiliations:** † Scuola Normale Superiore, Piazza dei Cavalieri 7, 56126 Pisa, Italy; ‡ Physical Chemistry Sector, Department of Chemistry, University of Ioannina, 45110 Ioannina, Greece; § Computational Chemistry and Biology Group, CCBG, DETEMA, Facultad de Química, Universidad de la República, 11400 Montevideo, Uruguay

## Abstract

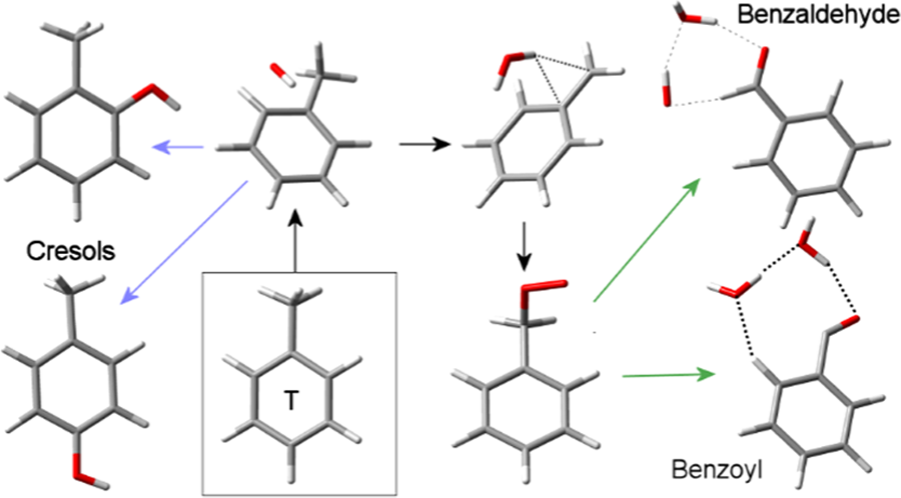

In a previous work, we have investigated the initial steps of the reaction of toluene with the hydroxyl radical using several quantum chemical approaches including density functional and composite post-Hartree–Fock models. Comparison of H-abstraction from the methyl group and additions at different positions of the phenyl ring showed that the former reaction channel is favored at room temperature. This conclusion appears at first sight incompatible with the experimental observation of a lower abundance of the product obtained from abstraction (benzaldehyde) with respect to those originating from addition (cresols). Further reactions of the intermediate radicals with oxygen, water, and additional OH radicals are explored in this paper through theoretical calculations on more than 120 species on the corresponding potential energy surface. The study of the addition reactions, to obtain the cresols through hydroxy methylcyclodienyl intermediate radicals, showed that only in the case of *o*-cresol the reaction proceeds by addition of O_2_ to the ring, internal H-transfer, and hydroperoxyl abstraction and not through direct H-abstraction. For both *p*- and *m*-cresol, instead, the reaction occurs through a higher-energy direct H-abstraction, thus explaining in part the observed larger concentration of the ortho isomer in the final products. It was also found that the benzyl radical, formed by H-abstraction from the methyl group, is able to react further if additional OH is present. Two reaction paths leading to *o*-cresol, two leading to *p*-cresol, and one leading to *m*-cresol were determined. Moreover, in this situation, the benzyl radical is predicted to produce benzyl alcohol, as was found in some experiments. The commonly accepted route to benzaldehyde was found to be not the energetically favored one. Instead, a route leading to the benzoyl radical (and ultimately to benzoic acid) with the participation of one water molecule was clearly more favorable, both thermodynamically and kinetically.

## Introduction

Gas-phase atmospheric chemistry of organic species is a subarea of research that has received considerable attention.^1^ Because of its importance, the atmospheric reaction of the hydroxyl radical, OH, with organic compounds (especially aromatic species) deserves particular consideration.^2−4^

Toluene (methylbenzene) is a major volatile aromatic constituent of gasoline. Many experimental studies have been performed on the combustion and atmospheric oxidation of this species, which is predominantly initiated by OH radicals. Because of its presence in gasoline and in cars’ exhaust, toluene is one of the most important contaminants in urban areas, where it can reach some 10 ppbv levels in polluted air^5^ (and even 1 ppmv in or near refueling stations^6^). Being ubiquitous in urban atmospheres, toluene is a precursor of tropospheric ozone and aerosol (smog).^7^ Previous work on aqueous solutions of benzene showed that the initial reaction with the OH radicals produced hydroxy cyclohexadienyl radicals.^8^ Extension of this study to toluene^9^ led to the same conclusion, that is, ring addition was the predominant mechanism. Additional information about the behavior of toluene and other aromatics in the atmosphere can be found in reviews like those of Calvert et al.^10^ and Vereecken.^11,12^ It must be pointed out that a very recent study by Zhang et al.^13^ reported that about 30% of the products of the reaction of toluene with OH does actually come from H-abstraction from the methyl group even at room temperature.

Several studies have been devoted to the determination of the rate constants for the oxidation of toluene. Davis et al.^14^ were the first to determine the absolute rate constants for the reactions of OH with benzene and toluene at 300 K. They obtained values of 1.59 × 10^–12^ and (6.11 ± 0.40) × 10^–12^ cm^3^ molecule^–1^ s^–1^, respectively, the latter corresponding to global H-abstraction (rate constant *k*_1a_) and ring addition reactions (rate constant *k*_1b_) at a total pressure of 100 Torr of He. They interpreted these data in terms of hydroxyl radical reactions with toluene both through pressure-dependent OH addition to the aromatic ring and via pressure-independent hydrogen abstraction from the side-chain methyl group. Doyle et al.^15^ studied the reaction rate of selected aromatic compounds, including toluene. They found a rate constant of (4.15 ± 1.49) × 10^–12^ cm^3^ molecule^–1^ s^–1^ at 304 K. Hansen et al.^16^ determined the absolute rate constants for the reaction of OH radicals with a series of aromatic hydrocarbons at room temperature using a flash photolysis-resonance fluorescence technique. In the case of toluene, they found a value of (5.78 ± 0.58) × 10^–12^ cm^3^ molecule^–1^ s^–1^. Perry et al.^17^ determined *k*_1a_ = 1.0|_–0.3_^+0.5^ × 10^–12^ cm^3^ molecule^–1^ s^–1^ and *k*_1a_/(*k*_1a_ + *k*_1b_) = 0.16|_–0.5_^+0.7^, finding also that the stabilization energy of the OH-aromatic adduct would be 16.5 ± 5 kcal mol^–1^, with a heat of formation of 0.8 ± 3 kcal mol^–1^. Bandow et al.^18^ studied the photo-oxidation of toluene in air and in the presence of NO*_x_*. They reported the appearance of ring-cleavage products like methylglyoxal, glyoxal, and maleic aldehyde, observing also the formation of small molecules like formaldehyde and formic acid. Also, Ohta and Ohyama^19^ and Bourmada et al.^20^ studied the system at different temperatures and pressures, determining gas-phase rate constants of (6.37 ± 0.08) × 10^–12^ and *k*_∞_ = (6.0 ± 0.7) × 10^–12^ cm^3^ molecule^–1^ s^–1^ for the high-pressure limiting rate constant, respectively. Tully et al.^21^ investigated toluene and several deuterated isomers to explore the competition between OH addition and hydrogen abstraction. At low temperatures (250–298 K), they found an activation energy of 0.54 ± 0.44 kcal mol^–1^ and a rate constant of 6.36 ± 0.69 cm^3^ molecule^–1^ s^–1^. According to Davis et al.^14^ and Tully et al.,^21^ this rate constant shows a significant pressure dependence, with its value approximately doubling when the He pressure is varied between 3 and 100 Torr. Knispel et al.^22^ also studied the formation of the radical adduct between OH and toluene. Using single temperature evaluation data, they found rate constants of (0 ± 0.20) × 10^–12^ and (7.0 ± 2.1) × 10^–12^ cm^3^ molecule^–1^ s^–1^ at 299 K for the abstraction and addition reactions, respectively, somewhat outside the error bars of the values by Davis^14^ and Bourmada,^20^ although in those cases, no hydroxylated products had been found. A fitting of the data for the abstraction reaction using the Arrhenius equation yielded an activation energy of −1.99 ± 0.26 kcal mol^–1^.

With respect to product yields, older results gave an interval of [5–23%] for benzaldehyde. It was also reported that ring OH-addition product radicals could react further with oxygen to end up in quinones or open cycles, through bicyclic intermediate structures (bicyclic peroxide structures were also studied experimentally in gas phase by Bohn^23^ and theoretically by Suh et al.^24^). Similar results were found by Seuwen and Warneck.^25^ The mechanisms of reaction are further complicated by the presence of NO. This was demonstrated in the work by Atkinson et al.^26^ who found that under their experimental conditions, the hydroxy cyclohexadienyl radicals formed from OH radical addition to the aromatic ring react with NO rather than with O_2_.

Klotz et al.^27^ studied the photo-oxidation of toluene/NO*_x_*/air mixtures. The yield obtained for benzaldehyde, 5.8 ± 0.8%, is within the range of values reported in previous studies (see their Table 2)—from 5.3 to 12%—albeit somehow in the lower region. *o*-, *p*-, and *m*-cresol were obtained in 12.0 ± 1.4, 3.2 ± 0.6, and 2.7 ± 0.7% yields. Although the comparison could not be done for all of the isomers, the results for *o*-cresol are lower than all previous results, which range from 12.3 to 38.5%. The considerable discrepancy among the experimental values reflects different experimental conditions. In particular, the reaction with NO_3_ radicals has been shown to be a sink for cresols. Formation of these species, on the other side, seems to follow a very simple mechanism where the reaction with O_2_ does not involve any intermediate but takes place directly to give the cresol plus the HOO^•^ radical. These results, as well as the yield of the *o*-cresol, are in good agreement with the most recent papers by Atkinson and Aschmann^28^ and Smith et al.^29^ In the latter work, however, benzaldehyde was not found and the major components were glyoxal and methylglyoxal, resulting probably from ring-cleavage mechanisms (Volkamer et al.^30^ and Gómez-Álvarez et al.^31^). Baltaretu et al.,^32^ using the turbulent flow chemical ionization mass spectrometry technique at temperatures ranging from 228 to 298 K, concluded that at those temperatures the glyoxal/methylglyoxal products might be obtained from secondary reactions.

While the situation in the gas phase appears to be clear, the results in solution are quite different. Tomat and Rigo^33^ produced OH radicals through the Fenton reaction of Fe^2+^ with H_2_O_2_ and studied their reaction with toluene. They concluded that the observed results were consistent with a primary H atom abstraction from the methyl group, leading to benzaldehyde with a yield larger than 60%. They also observed that benzyl alcohol (BA) was produced in small quantities in some of the experiments, in particular, when the concentration of Fe^3+^ was small. Two important conclusions were reached in this paper. On the one side, aromatic ring hydroxylation products were not observed. On the other side, benzoic acid was not identified under those reaction conditions. Hatipoglu et al.^34^ performed a study of the photo-oxidative degradation of toluene in aqueous solution by the hydroxyl radical. This combined experimental/theoretical study was performed using ultraviolet excitation of nitrate as a source of OH radicals to generate the products. Benzaldehyde and cresols were observed as final products, with yields of 30.5 ± 6.0% for *o-*cresol (the dominant pathway according to density functional theory (DFT) calculations), 47.0 ± 10.1% for the combined *m-* and *p-*cresols, and 17.0 ± 3.3% for benzaldehyde. It is noteworthy that, while the yield of benzaldehyde is within the range of previous results, the combined yield of cresols (77.5%) is significantly higher than previous estimates. This fact may be a result of lack of reaction between cresols and NO*_x_* radicals in this experiment. The corresponding yields provided by rate constants obtained from computational simulation in aqueous solution were 58.6, 30.7, and 10.7%, respectively, in qualitative agreement with the experimental results in the gas phase but in disagreement with the results of Tomat and Rigo.^33^

Summarizing the available information, one can say that there are three different situations in which the toluene reaction with hydroxyl radicals can occur: at high temperature (combustion), in the gas phase (atmospheric), and in aqueous solutions. Water presence may also be important in atmospheric chemistry (see, for instance, the work by Li et al.^35^). One of the particularities of aqueous solutions is that they can actually affect the reactivity of the hydroxyl radical, possibly through a cage effect as suggested by Kopinke and Georgi.^36^ Leaving aside the high-temperature situation, the experimental and theoretical information available till now is quite ambiguous concerning the appearance of ring-cleavage products, the paths leading to benzyl alcohol and benzaldehyde, the more important pathways in the absence of NO*_x_* radicals, and the detailed mechanism of secondary reactions with the hydroxyl radical and oxygen. To complicate matters a little further, in a recent paper,^37^ we have presented theoretical evidence that formation of the benzyl radical seems to be thermochemically and kinetically more favorable than formation of the hydroxy cyclohexadienyl radicals. This information led us to conjecture that cresols must also be formed by somehow “hidden” routes not yet described, which could explain their prevalence over the benzaldehyde obtained from the more abundant benzyl radical.

On these grounds, we have performed a comprehensive theoretical analysis of the possible pathways for the oxidation of toluene in the absence of NO*_x_* radicals, with the aim of explaining the apparent discrepancies between the relevant abundances of the intermediate radicals and the final products.

## Methods

Equilibrium geometries and thermodynamic and kinetic properties were systematically obtained using DFT methods. We chose the M06 exchange-correlation functional,^38^ in view of its accurate description of main-group bond energies (mean unsigned error (MUE) = 1.8 kcal mol^–1^) and noncovalent interactions (MUE = 0.4 kcal mol^–1^). The M06 method depends on parameters that were optimized using different basis sets. Since this is a factor that may influence our own results, we tried a limited variation of the basis sets employed. We selected the 6-311++G(3df,2pd)^39^ basis set as the standard option but also performed calculations using both a smaller one (6-31+G(d,p)) and a more extended one, the cc-pVQZ Dunning basis set,^40^ to analyze basis set effects. Since during the preparation of this paper we became aware of a paper by Truhlar and co-workers showing the excellent performance of the M06-2X functional,^13^ we also included this DFT method in our study.

Furthermore, two composite models were used for the refinement of the energetic properties of the species involved, namely, the CBS-QB3 method of Peterson et al.^41−43^ and the G4 method of Curtiss et al.^43^ These methods account for basis-set extension and correlation energy effects by additive schemes on top of B3LYP equilibrium geometries and frequencies. Both of them are approximations, increasingly accurate, to CCSD(T)/CBS calculations, which are not feasible on molecules of this size. The estimated average errors of CBS-QB3 and G4 methods for a large series of molecules are below 2 kcal mol^–1^ and often around 1 kcal mol^–1^.

As usual, eigenvalues of the Hessian were checked for the critical points to assure the correct number of negative eigenvalues for transition states. Geometry optimizations were performed in all cases until Cartesian coordinates were accurate at least to 1 × 10^–4^ Å. An ultrafine grid was used for integration of the density in the density functional calculations (for generalities on the computational methods used, see Jensen^44^).

All calculations were performed with the G16 suite of computer codes.^45^

## Results and Discussion

As already mentioned, the purpose of the paper is to analyze the different reaction paths for the attack of toluene by the hydroxyl radical. Since Uc et al.^46^ found that ring hydrogen abstraction is significant only at high temperatures, we considered only H-abstraction from the methyl group and hydroxyl radical additions to the ring. Secondary reactions with OH and O_2_ were also included in this study.

**Scheme 1:**
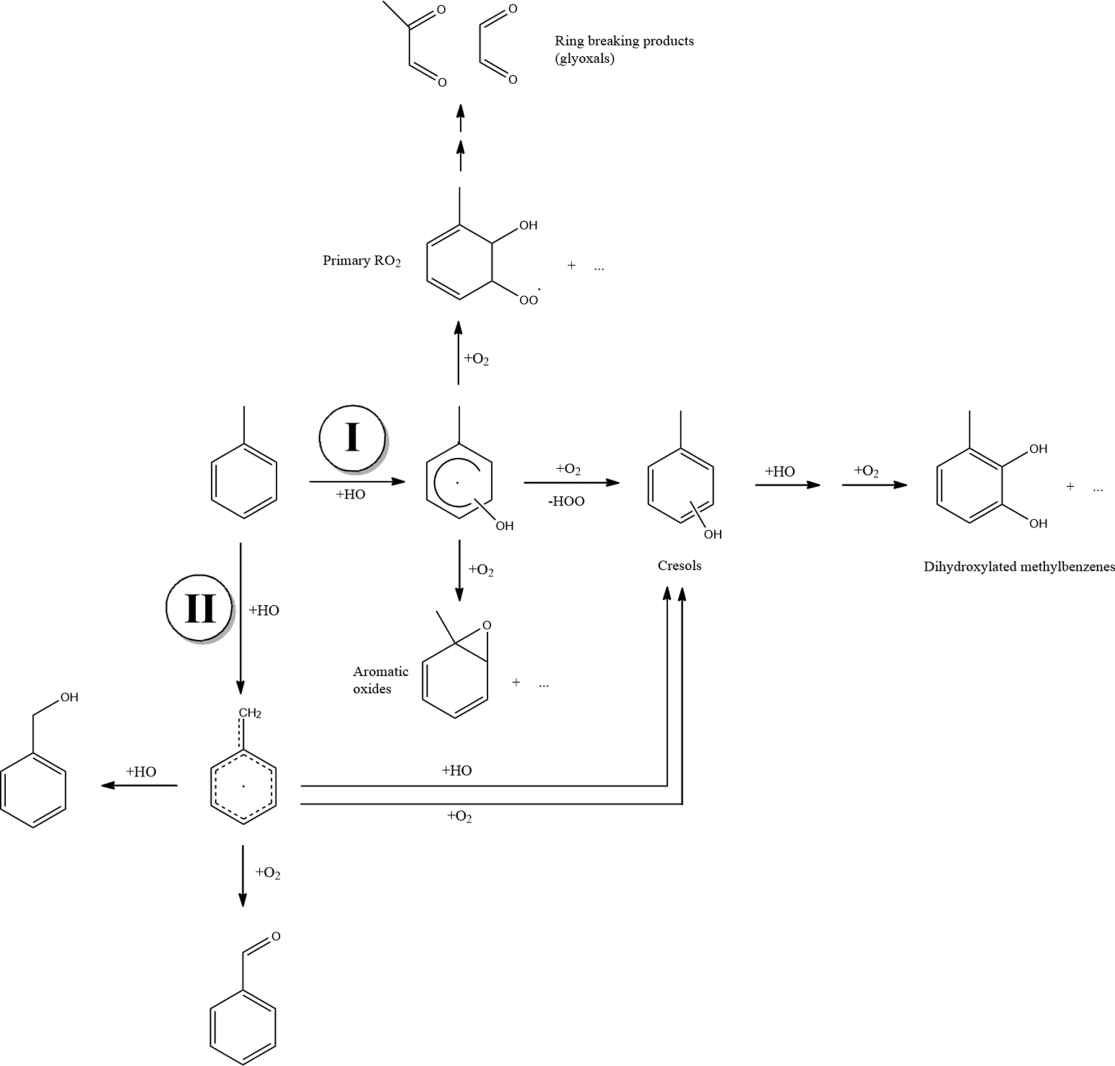
Possible Reaction Paths after Primary Attack of the Hydroxyl Radical on Toluene in the Absence of NO*_x_* Radicals

**Scheme 2:**
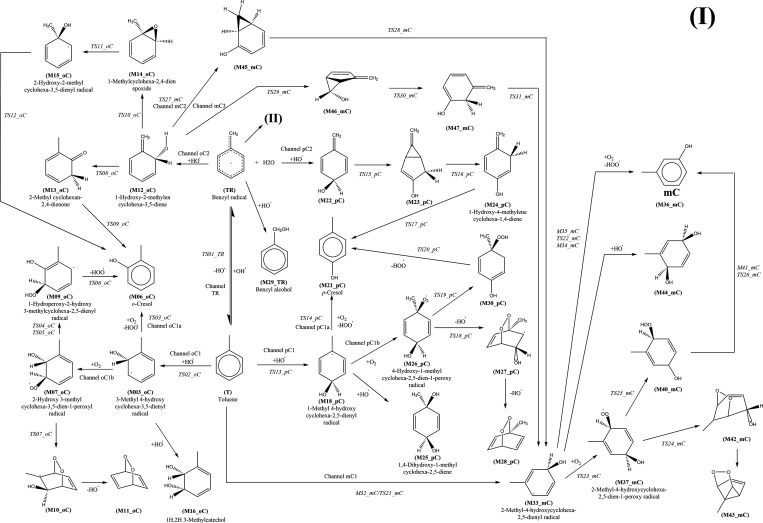
Structure of the Intermediates and Products Identified on the Potential Energy Surface (PES) of the Initial Reaction of Toluene with OH and Further Reactions with OH, H_2_O, and O_2_

**Scheme 3:**
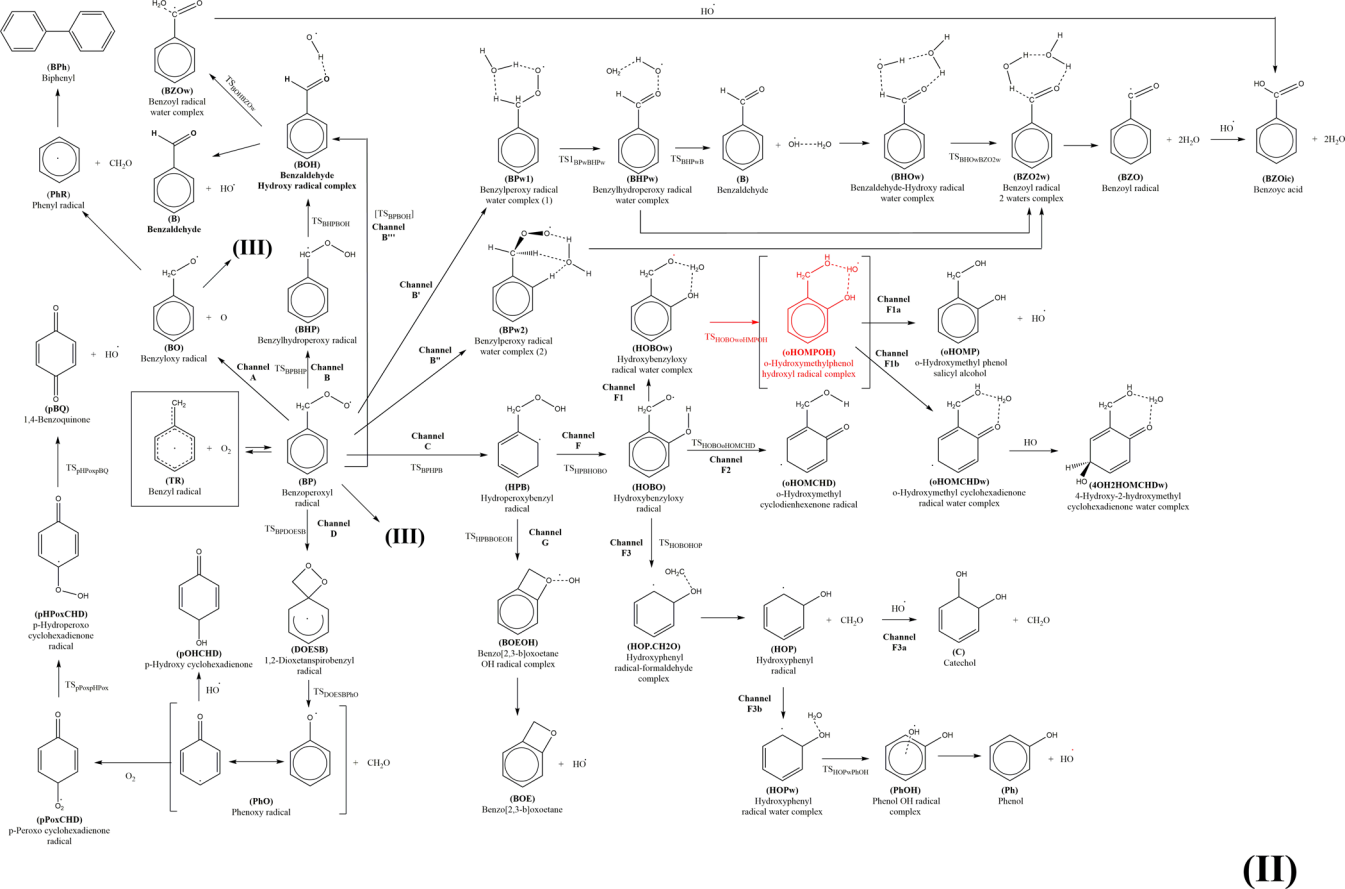
Reactions Identified on the Potential Energy Surface for the Reaction of the Benzyl Radical and the Oxygen Molecule

**Scheme 4:**
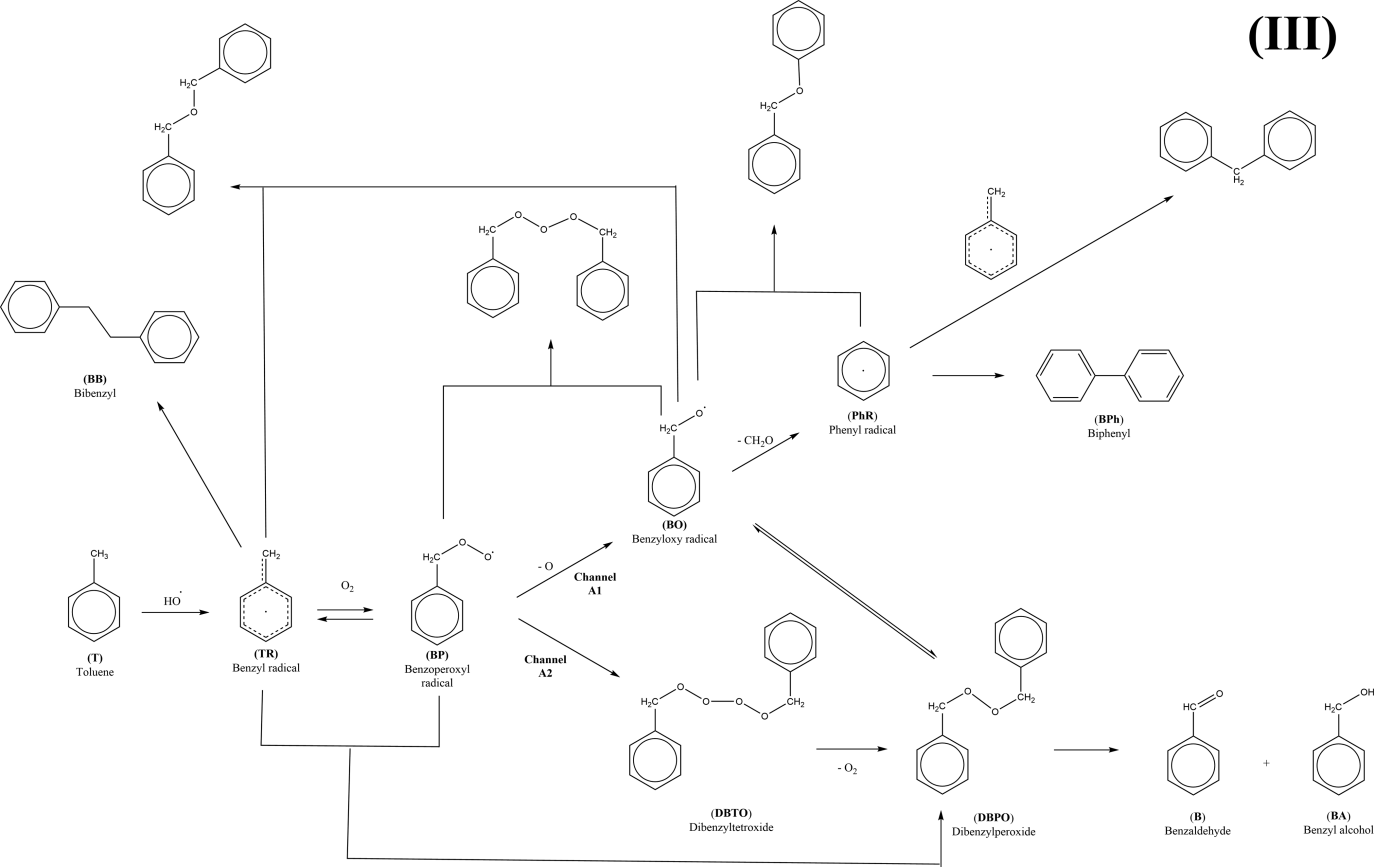
Dimerization of Benzyl, Benzoperoxyl, and Phenyl Radicals (PhR), Reaction of the Tetroxide to Peroxide, and Decomposition of the Peroxide into Benzaldehyde and Benzyl Alcohol

The whole degradation mechanism of toluene can be divided, in principle, in two sets, including ring-preserving reactions and ring-breaking reactions, respectively (see Scheme 1). The products of the first class of reaction paths are benzaldehyde, benzyl alcohol, cresols, and other ring-preserving derivatives, which will be described later (see Schemes 2 and 3). The second set of reactions would lead to glyoxal, methylglyoxal, and other dialdehydes, which have not been considered yet. Aromatic oxides are possible intermediates, which are then easily transformed. The presence of OH, H_2_O, and O_2_ in excess may lead to further reactions, eventually giving dihydroxylated products (Scheme 2). Additionally, the reaction of the benzyl radical with those species can lead to salicyl alcohol, benzoic acid, phenol, catechol, *p*-benzoquinone, and bicyclic products (Scheme 3). Finally, dimerization reactions are also possible (see Scheme 4), but they will not be considered in the present study. Our computational strategy was validated in ref (47) against experimental values of the enthalpies of formation of some of the species in Schemes 2 and 3.

From the initial calculations performed at the simple M06/6-31G(d,p) level, it was clear that the most important reactions are those leading to the benzyl radical and the *o*-hydroxy methylhexadienyl radical, and the least important reaction is that leading to the *m*-hydroxy methylhexadienyl radical. Structures and geometrical parameters of the reactants, intermediates, products, and transition states found on all of the paths studied are given in the Supporting Information (SI) section.

### Routes toward Cresols

**Figure 1:**
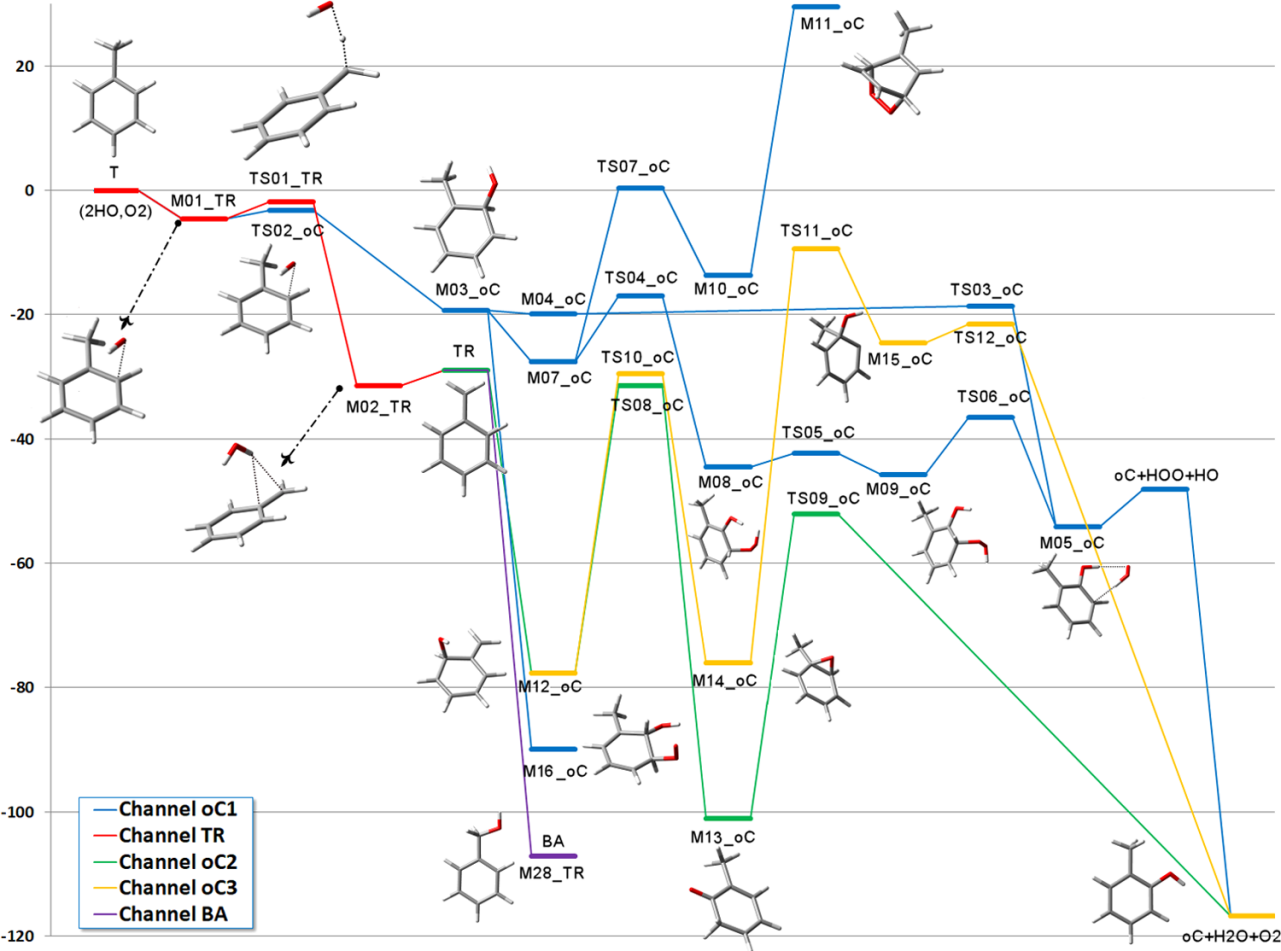
Reaction paths for the formation of *o*-cresol. G4 relative energies Δ(*E* + ZPE) in kcal mol^–1^ are calculated with respect to toluene plus two OH radicals and one O_2_ molecule.

The G4 energies (including zero point energy (ZPE)) of the intermediates and transition states in the TR and oC paths are collected in Figure 1. The reader may want to consult our previous study^37^ to have a global appraisal of the mechanism. As we showed there, the transition states TS01_TR and TS02_oC lead to M02_TR (the water-complexed benzyl radical) and M03_oC (the *o*-hydroxy methylcyclohexadienyl radical), respectively. We have also discussed^37^ the existence of a much less stable PRC where the OH interacts with the hydrogens of the CH_3_ group, directly on the opposite side of the ring. However, this complex is less stable and evolves toward the same transition state TS01_TR. Therefore, it was not considered further in this study. M03_oC has three open channels under different conditions. If OH is present in excess, then the route to the *o*,*m*-dihydroxy toluene (M16_oC) is favored. We had already shown the modifications that occur in the mechanism of oxidation of dimethyl sulfide (DMS) when excess OH is present;^48,49^ this is a similar case, but with another addition to the ring instead of abstraction. If that is not the case, then the reaction with O_2_ is favored, either by attack to the hydrogen ipso to the OH (M04_oC) (later passing through the transition state TS03_oC with a small barrier giving the cresol) or through bonding with the carbon bearing the lone electron in the ring (M07_oC).

As can be seen in Figure 1, the path leading to M07_oC is initially more favorable but the transition state for the hydrogen transfer from carbon to oxygen, TS04_oC, is above TS03_oC. Therefore, the route to cresol should proceed preferentially through hydrogen abstraction and not through O_2_ attachment to the ring. There is a secondary path, leading from M07_oC to the bicyclic peroxide M11_oC through O_2_ addition to M10_oC and then OH loss to M11_oC, but the transition state TS07_oC and the end product are much higher and the path is unlikely.

On the other side, if OH is present in excess, TR can give not only benzyl alcohol (M28_TR, BA), where the hydroxyl radical is added to the methylene carbon, but also M12_oC, where the hydroxyl radical is attached at the ortho position in the ring. This hydroxy methylenehexadiene species is less stable than BA but is also formed without a barrier and will be in equilibrium with it. In principle, two paths starting from M12_oC are possible, one involving a bicyclic epoxide (M14_oC) and the other a very stable ketone (M13_oC). While the former path requires overcoming transition states that are quite high in energy (TS11_oC and TS12_oC), the transition state for the shift of the H atom from C to O in the ketone (TS09_oC) is the lowest one present in the whole reaction path. Therefore, this is another feasible route for the formation of cresol. Notice that in this case, the rate-determining transition state is TS08_oC, which is submerged by about 30 kcal mol^–1^ with respect to the isolated reactants.

From the previous analysis then, one can conclude that if OH is present in excess, the route through the hydroxy cyclohexadienyl radical leads directly to the dihydroxy species, while the route through TR leads to benzyl alcohol and *o*-cresol. If, instead, OH is not in excess, the cresol is formed from the hydroxy cyclohexadienyl radical through a less favorable (i.e., slower) route.

A remark about benzyl alcohol is in order here. Although BA is not normally reported as a product in the oxidation of toluene in the atmosphere or reaction chambers, its presence together with cresols and benzaldehyde has been reported in high-temperature oxidation studies of the methyl side chain of toluene. Brezinsky et al.^50^ found a significant amount of benzyl alcohol (see their Figures 5 and 6) in both lean and rich toluene oxidation and suggested that it results from the association reaction of benzyl and hydroxyl radicals, as we propose here for BA. It should also be mentioned that, in another context (visible light irradiation of aqueous solutions containing toluene, uranyl ions, and oxygens), BA was found alongside benzaldehyde by Mao and Bakac.^51^ Seuwen and Warneck^25^ also obtained benzyl alcohol and benzaldehyde in the oxidation of toluene in air. When the reaction was initiated by removal of a methyl hydrogen by chlorine, only these two products were identified. When photolysis or hydroxyl radicals were used to obtain the benzyl radical, then more complex reactions and a number of other products were also found. Furthermore, direct hydroxylation of toluene in a micro-dielectric barrier discharge (DBD) plasma reactor was shown to produce BA.^52^

**Figure 2:**
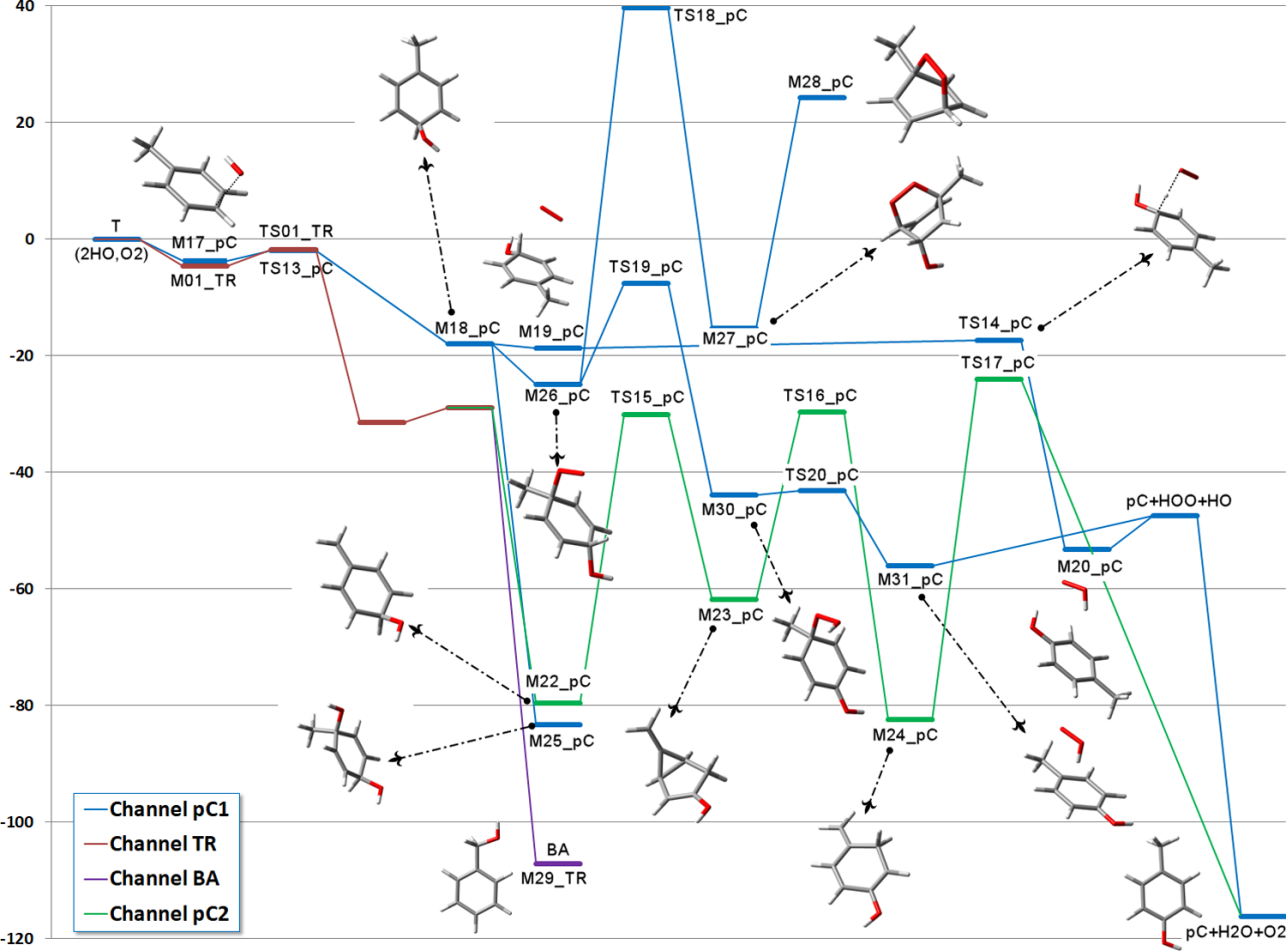
Reaction paths for the formation of *p*-cresol. G4 relative energies Δ(*E* + ZPE) in kcal mol^–1^ are calculated with respect to toluene plus two OH radicals and one O_2_ molecule.

Figure 2 is analogous to Figure 1 for the case of *p-*cresol. The reaction paths starting from the hydroxy methylcyclodienyl radical are similar to the case of *o*-cresol, but now there is only one pC2 path open starting from M22_pC (OH addition to the ring instead than to the methylene group). However, the heights of the barriers make this path unlikely, especially because of TS17_pC that, although being submerged, lies above the energy of M26_pC and M02_TR. Therefore, one can conclude that if OH is not present in excess, *p-*cresol will be formed by the ordinary route starting from the hydroxy methylhexadienyl radical. Instead, if OH is in excess, then only the dihydroxy species and benzyl alcohol would be obtained. This is an experimentally testable prediction. Since only the production of the *p-*cresol is affected, the ratio of concentrations *o-*cresol/*p-*cresol should increase with the increment of OH concentration. An experiment in a reaction chamber that measures the amounts of *o*-cresol in relation to *p*- and *m*-cresol (this last one is not shown in this paper, but it is similar to para) would confirm or disprove the mechanisms presented.

### Fate of the Benzyl Radical: The Benzaldehyde Pathway

Due to its unusual structure—a conjugatively stabilized radical without any easily breakable C–C bonds—the benzyl radical is relatively stable. It is well known that alkyl radicals can associate with molecular oxygen to give the peroxyl radical, both in combustion and in the atmosphere. Thus, one of the reaction paths we explored is the association of the benzyl radical with molecular oxygen, which has been studied experimentally by Fenter et al.,^53^ Nelson et al.,^54^ and Elmaimouni et al.^55^ and theoretically by several authors.^56−61^ Although the reaction of the benzyl radical with O_2_ is believed to occur without a barrier, Zhang et al. obtained a very small transition state at high levels of theory.^62^

It is known that benzaldehyde is one of the major products obtained from the reaction of benzyl radical with O_2_.^58^ The obvious reaction path seems to be the direct abstraction of a hydrogen atom from the CH_2_ group, as studied at the semiempirical level by Clothier et al.,^56^ at the CBS-QB3 level by Murakami et al.,^57^ and at several DFT and ab initio levels by Canneaux,^58,59^ Sander,^60^ and Pelucchi et al.^61^ In the present work, moreover, after reanalyzing the reaction mechanisms proposed by Murakami et al. (using the chemical models discussed above), we further explored the possibility of water participation, i.e., when the reactions occur in solution or in atmospheric conditions. We will show that, contrary to the case studied by Murakami et al., in those conditions the most stable product is not benzaldehyde per se but the benzoyl radical, which can proceed further to other products.

**Figure 3:**
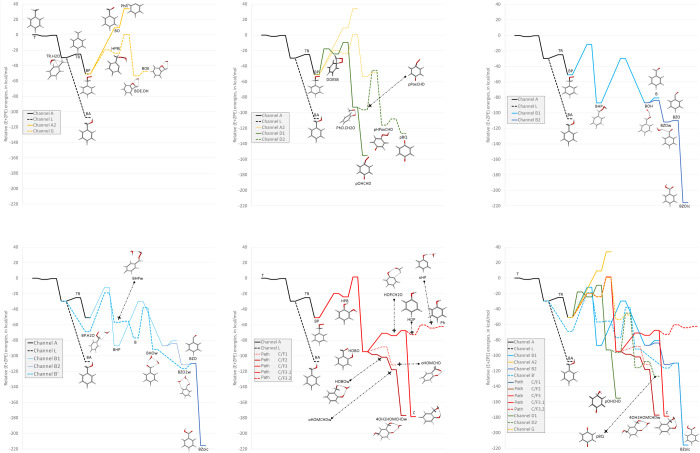
Scheme of the possible reactions of TR with O_2_, including secondary reactions with additional OH and O_2_ species; G4 relative energies Δ(*E* + ZPE) in kcal mol^–1^.

The general energy scheme for the reaction of the TR radical with oxygen is shown in Figure 3, split for convenience in several panels. The channels are labeled in the same way as in the work of Hatipoglu et al.,^34^ and the structures, included as small images in the reaction channels, are fully displayed in the Supporting Information section (images as well as Cartesian coordinates of the species). By way of comparison, the energy of benzyl alcohol has also been included.

The initial step of the reaction is the formation of the benzylperoxyl radical, which has been studied in the literature several times. Nelson and McDonald^54^ determined that the second-order reaction is independent of temperature and pressure. Later, Elmaimouni et al.^55^ studied again this reaction and determined both the rate coefficient and the reaction enthalpy, Δ*H*_r,298_° = −(20 ± 1) kcal mol^–1^. Almost simultaneously, Fenter et al.^53^ studied the thermochemistry and kinetics of this reaction. They determined the rate constant and also derived the enthalpy of reaction, Δ*H*_r,298_° = −(21.5 ± 1.5) kcal mol^–1^, claiming that this new value is more precise. As can be seen in the SI, the value we calculated at the G4 level, −22.7 kcal mol^–1^, is well within the error margins of the experimental derivation. They derived also the enthalpy of formation of the benzoyl peroxide (BP) radical as Δ*H*_f,298_° = 27.9 ± 1.5 kcal mol^–1^. Our theoretical result, published together with others of the intermediates and products in this PES,^47^ is also in agreement with the experimental value. The radical and the isomerization reaction to benzylhydroperoxy radical (BHP), which will be discussed in the following section, were studied extensively by Canneaux et al.^58,59^ The most recent papers on BP were produced by Sander et al.,^60^ who used matrix-isolation IR and electron paramagnetic resonance (EPR) spectroscopy and theoretical methods to follow the photolysis of BP to benzaldehyde and the benzoyl radical; Zhang et al.,^62^ who used CASPT2 to study the recombination reaction of several aromatic radicals with molecular oxygen; and Pelucchi et al.,^61^ who used DFT and high-level ab initio calculations.

Several reaction channels originate from BP: channel A, leading to the phenyl radical (PhR) and formaldehyde through the benzyloxyl (BO) radical, is clearly unfavorable and would probably be of marginal significance in combustion processes where, eventually, dimerization as in Scheme 4 may preferably occur. Dimerization of the BO and PhR radicals would lead to channel A1, which is not further investigated in the present paper (only the biphenyl structure is included in the SI). Notice that there is an alternative path ruled by lower energy barriers, path C/G, which leads to a bicyclic benzooxetane (BOE) product through release of a hydroxyl radical. This reaction path starts from BP and through a six-center transition state (TS_BP_HPB) proceeds toward a hydroxyperoxybenzyl (HPB) radical intermediate. The energy barrier governing this path amounts to 31.2 kcal mol^–1^ at the G4 level, but the TS remains submerged by 19 kcal mol^–1^ with respect to the reactants. Comparison with the TR intermediate and the O_2_ addition shows that HPB is just 5.7 kcal mol^–1^ less stable. The relative heights of the forward and backward barriers suggest that detection of BHP might be quite difficult, but this species could actually isomerize, through ejection of the OH radical, leading to the formation of the bicyclic benzooxetane (BOE) product. When complexed with OH (BOE·OH), this product is actually more stable than BP. The problem with this path is that it is also unfavorable due to a high-energy transition state (TS_HPB_BOEOH), which requires an activation energy of 25 kcal mol^–1^, thus being not submerged with respect to toluene (the TS lies at 0.7 kcal mol^–1^ with respect to reactants). What looks intriguing in this reaction path is that if the BOE·OH complex would be formed at all, the reverse path to BHP is less favorable, i.e., the OH radical would not be added easily to BOE. Finally, notice that the reaction channel G leading to HPB will compete with the other channel studied by Canneaux,^58,59,^ Sander,^60^ and Pelucchi^61^ where the peroxyl moiety abstracts a hydrogen from the CH_2_ group through a four-center TS. This reaction, which we identified as reaction path B in this paper, will be discussed later.

HPB can also follow another reaction path, referred to in the following as the F channel. The central transformation in this case is the transfer of the terminal OH radical from the hydroperoxybenzyl radical to the ortho carbon atom in the cycle. This occurs through a transition state (TS_HPB_HOBO) that lies at only 25.5 kcal mol^–1^ over HPB (better than for the G path) and also over the energy of the initial reactants by 1.5 kcal mol^–1^. This is a five-center transition state (see the SI), showing some conformational flexibility and ruling reaction channels that, to the best of our knowledge, were never analyzed before. Assuming for the time being that this could be a feasible reaction, the HOBO intermediate formed is very stable (−94.6 kcal mol^–1^ below TR + O_2_) due to the very strong hydrogen bond between the −CH_2_O radical group and the OH group on the ring. Three interesting paths open from this stable intermediate.

The most straightforward channel is F2, involving a hydrogen transfer between the −CH_2_O and −OH groups, which generates the ketone shifting the unpaired electron from the side chain to the inside of the group. In the same way as PhO in channel D, the para position is the most favorable one for accumulation of the electronic density and the *o*-hydroxymethyl cyclodienhexenone radical oHOMCHD would be obtained. This requires a small barrier (6.0 kcal mol^–1^) to overcome the TS_HOBO_oHOMCHD and would be an easy transformation with an enthalpy gain of 17.2 kcal mol^–1^ below HOBO. Now (at least) two different possibilities open for oHOMCHD. On the one side, it may react in a similar way to PhO in channel D, since the difference is only the −CH_2_OH lateral chain. Thus, an *o*-hydroxymethyl-substituted *p*-hydroxy cyclohexadione or 1,4-benzoquinone would be produced. We have not studied this channel further, but it would be interesting to compare the relative energies of the products with respect to their nonsubstituted counterparts. The second channel involves the interaction with a water molecule. Remember that water molecules were produced in the initial reaction of toluene with OH, so that they are ready to be used, in addition to any water molecule possibly present in the environment. The water molecule may disrupt the intramolecular hydrogen bond, replacing it with a pair of intermolecular hydrogen bonds and producing the oHOMCHDw water complex, which lies 6.2 kcal mol^–1^ below oHOMCHD. This species may then follow the same paths as before, reaction with OH or O_2_, according to what is available. In the reaction scheme and energy diagrams, we have included only the 2-hydroxymethyl cyclohexadienone water complex to avoid unnecessary complication.

Complexation with water in the F2 channel may seem a trivial endeavor, but it has a connection with channel F1. It starts with the replacement of the intramolecular hydrogen bond in HOBO, before hydrogen transfer, with a pair of intermolecular hydrogen bonds when a water molecule is introduced. This implies a smaller stabilization than in the case of oHOMCHD (3.8 vs 6.2 kcal mol^–1^) but a lower energy nonetheless. If this complex is formed (which cannot be taken for granted in view of the opposite trends of enthalpic and entropic contributions), then water can act as a bridge for the hydrogen transfer, making it essentially effortless to obtain oHOMCHDw. Regretfully, we were not able to locate either the intermediate oHOMPOH or the transition states that connect it to HOBOw and oHOMCHDw, admitted that they exist at all. Moreover, notice that if this intermediate is present in general, it would be the product of the attack of an OH radical to salicyl alcohol (oHOMP). The separated “products” oHOMP and OH are located over HOBOw by 17.5 kcal mol^–1^, suggesting that the enthalpic contribution disadvantages this route.

A third possible path starting from HOBO is the ejection of formaldehyde to form the hydroxyphenyl radical HOP. Overcoming the TS_HOBO_HOP transition state requires an energy similar to those ruling the alternative paths described till now, but the reverse barrier from the HOP·CH_2_O complex to HOBO is much smaller than the direct one (3.0 vs 23.6 kcal mol^–1^), making the formation of the product quite unlikely. In the case this path is followed, two alternative channels are open. On the one side, channel F3a would lead to catechol C by a direct reaction with an additional OH radical. On the other side, going through channel F3b, it may acquire a hydrogen atom from water, thus producing phenol PhOH and releasing an OH radical. This has been characterized as a multistep possibility in the detailed study by Bounaceur et al.,^63^ but they attributed the formation of phenol to a reaction of the benzyl radical, which we showed to be much higher in energy; Metcalfe et al.^64^ did the same in their comprehensive detailed chemical kinetic modeling study of toluene oxidation. Again, we see a delicate equilibrium between the OH-consuming and OH-producing routes starting from the same intermediate.

Besides the already discussed H-abstraction paths (C/G/F), two other reaction channels arise from BP: path B, where the H-abstraction occurs from the −CH_2_ moiety instead than from the ring (the path studied also by Murakami,^57^ Canneaux,^58,59^ Sander,^60^ and Pelucchi^61^), and a ring-closure channel, channel D, where the spiro bicycle DOESB is formed.

Let us start by analyzing path D. The barrier to overcome TS_BP_DOESB is 33 kcal mol^–1^, slightly higher than that needed to overcome TS_BP_HPB in channel C (31 kcal mol^–1^). The relative stability of DOESB is comparable to that of HPB (−24.2 vs −24.0 kcal mol^–1^), and both channels would then be accessible. The main difference between these paths lies in the secondary reactions. While channel G requires a non-negligible energy to eject the OH radical, DOESB can decompose to the phenoxy radical PhO and formaldehyde through a lower TS (TS_DOESB_PHO), which lies at −9.4 kcal mol^–1^ and requires an activation energy of 14.8 kcal mol^–1^. Thus, both kinetically and thermodynamically, the channel leading to the phenoxy radical plus formaldehyde is more favorable. Of course, now PhO has several open paths, depending on the relative abundance of OH and O_2_ in the environment (see panel 2 of Figure 3). If none of them is present in excess, reactions would not proceed further and the radical would dimerize. However, if there is OH in excess, then the *p*-hydroxy cyclohexadienone pOHCHD would be formed as the end product. This is a barrierless process, and the final enthalpy (−155.1 kcal mol^–1^) is much lower than that of other possible products like BA (−107.6 kcal mol^–1^) or *o*-cresol (−121.8 kcal mol^–1^).

If OH is scarce but O_2_ is abundant, then pPoxCHD can be formed, once again through a barrierless process. Notice that ortho substitution is also possible, but this possibility has not been analyzed further. The only reasonable possibility of further reaction to this radical would be either cyclization, through an attack of the terminal oxygen in the O_2_ moiety to the meta carbons, or abstraction of the hydrogen attached to the para carbon. The features of the most stable structure of pPoxCHD (see the SI) suggest that the more favorable abstraction channel goes through the four-center transition state (TS_pPoxCHD_pHPoxCHD) to move the radical center inside the ring. This path is ruled by a quite high energy barrier (51.1 kcal mol^–1^), which is nonetheless lower than the corresponding barrier governing the path leading to formation and decomposition of DOESB. As can be seen in panel 2 of Figure 3, the energy of this transition state is similar to the energy of BOE in the G channel, both of them lying below that of TR + O_2_. The process is thus feasible, and if pHPoxCHD is formed, then ejection of the OH radical requires to overcome a much lower barrier (only 8.0 kcal mol^–1^) to form the 1,4-dibenzoquinone pBQ. Formation of the quinone was experimentally found by Buth et al.^65^ but interpreted as the product of the reaction of PhO with oxygen atoms and ejection of a hydrogen.

It is important to notice that both in this path D and in channel G, an OH species is produced, which could be then available for further reactions. For instance, taking into account only the D path, channel D1 would give a more stable product than channel D2, but only channel D2 would produce the OH radical needed to open channel D1. Although this is, of course, an oversimplification, it illustrates well the role of complex equilibria in these reaction systems, especially under variable conditions of OH and O_2_ concentrations.

Notice that our D path is analogous to paths D/J/I in the paper by Murakami et al.^57^ They chose to stop the reaction there and did not explore the route leading to the cyclohexadienone or the quinone. Their CBS-QB3 computations placed the PhO + CH_2_O product at −65.4 kcal mol^–1^ with respect to TR + O_2_, while we got −63.0 kcal mol^–1^ at the G4 level and −65.0 kcal mol^–1^ at the M06/cc-pVQZ level. Pelucchi et al.^61^ also studied this path, and their W4 structure is the one we identified here as DOESB. They found W4 to lie 6.9 kcal mol^–1^ over TR + O_2_, while we got 5.5 kcal mol^–1^ at the G4 level and 10.0 kcal mol^–1^ at the M06/cc-pVQZ level, thus bracketing their value. Likewise, their TS2 transition state corresponds to our TS_BP_DOESB, which lies 11.9 kcal mol^–1^ (G4) or 16.3 kcal mol^–1^ (M06/cc-pVQZ) over TR + O_2_. By using their most accurate calculations (CCSD(T)-F12/VTZ-F12), Pelucchi et al. found a value of 12.5 kcal mol^–1^. The corresponding barriers were 34.6 kcal mol^–1^ (G4), 34.3 kcal mol^–1^ (M06/cc-pVQZ), 33.3 kcal mol^–1^ (Pelucchi et al.), and 32.4 kcal mol^–1^ (Murakami et al.). Finally, Pelucchi et al. found the PhO plus formaldehyde product at −64.6 kcal mol^–1^, again in agreement with our calculations. All methods are thus in agreement concerning this path.

The most studied path is path B (same name as in Murakami et al.^57^), namely, the abstraction of one hydrogen atom belonging to the −CH_2_O_2_ group by the terminal oxygen atom, giving rise to the benzyl hydroperoxyl radical BHP.^56−61^ This is a four-center transition state ruling the 1,3 H-shift that has been characterized at several theoretical levels. Our own results give a quite high barrier of 40.5 kcal mol^–1^ at the G4 level and 39.4 kcal mol^–1^ at the M06/cc-pVQZ level. The most sophisticated calculations by Sander et al.^60^ at the CCSD(T)/6-311++G(2d,2p)//M06-2X-6-311++G(2d,2p) level provided a value of 45.2 kcal mol^–1^. The presumably most accurate method employed by Canneaux et al.^58^ (CCSD(T)/cc-pVTZ//MPW1K/cc-pVZ) afforded a value of 40.2 kcal mol^–1^. The same authors also performed CASPT2 calculations, aimed to provide an estimate of the possible multireference character of this TS, but they used different basis sets for the calculation, as well as different methods for the geometry optimization. Their best result at the CASPT2/ANO-L-VDZP//MPW1K/cc-pVTZ level is 34.2 kcal mol^–1^, but it is difficult to say whether this low value is due to a true multireference character or, rather, to a too small basis set. Murakami et al.^57^ using the less demanding CBS-QB3 method obtained a barrier of 38.7 kcal mol^–1^, which is intermediate between the higher and smaller values. The most recent calculations, at the CCSD(T)-F12/VTZ-F12 level, performed by Pelucchi et al.^61^ led to a value of 38.3 kcal mol^–1^, in good agreement with the estimates of ref (57) and of the present study. Irrespective of its precise value (in the range between 34 and 45 kcal mol^–1^), the energy barrier is quite high. This would imply that this reaction is only feasible photochemically, at least in a waterless environment (vide infra). It is noteworthy that Sander et al.^60^ succeeded in producing this reaction experimentally.

The reaction of formation of BHP is almost thermoneutral according to the calculations by Sander et al.^60^ (3.0 kcal mol^–1^ in favor of BP). Murakami et al.,^57^ on the other side, did not find the BHP species at the CBS-QB3 level, in agreement with our results both at the CBS-QB3 and G4 levels. M06/cc-pVQZ computations predicted, instead, the existence of BHP and an enthalpy difference of 1.0 kcal mol^–1^ in favor of this species with respect to BP. Clearly, this is an effect of the underlying optimization method in CBS-QB3 and G4 (the B3LYP DFT method), which might have led to inaccurate structures of these species. Canneaux et al.^59^ reported results ranging between 2.8 and 8.5 kcal mol^–1^ in favor of BP. The situation in this case is clear; both isomers do exist if an accurate enough geometry optimization method is used. Moreover, the endothermicity or exothermicity of the reaction depends to a large extent on the quality of the computational method employed. It is safe however to say that the reaction is almost thermoneutral and that the species are separated by a high barrier. BHP in turn can easily dissociate to a complex of benzaldehyde and hydroxyl radical, which is just what is obtained directly from BP at the CBS-QB3 and G4 levels. Our M06/cc-pVQZ calculations place the TS_BHP_BOH transition state at only 0.8 kcal mol^–1^ over BHP. Sander et al.^60^ pointed out that they found no barrier at the B3LYP-D level, which is consistent with the nonexistence of BHP and TS_BHP_BOH at the CBS-QB3 or G4 level. The complex, however, is quite stable and easy to find at the different theoretical levels. They calculated a stabilization energy of −56.8 kcal mol^–1^ for a cyclic complex where the hydroxyl moiety is hydrogen-bound to benzaldehyde both through the hydrogen (to the carbonyl oxygen) and through the oxygen (to the ortho hydrogen in the cycle), like in our equivalent channel B1. At the M06/cc-pVQZ level, we found a singly hydrogen-bonded complex instead, with the oxygen pointing toward the H in the aldehyde group instead than to the ring. The stabilization enthalpy at 0 K was −57.3 and −54.4 kcal mol^–1^ at the G4 and M06/cc-pVQZ levels, thus bracketing the results of ref (60). In fact, inspection of the energy profile shown in Figure 4 of ref (60) shows that our complex is completely analogous to their B2 structure, which they include in the profile, instead of our B1, which they do not include. The complex BOH itself was not described by Murakami et al.,^57^ who only report the end product benzaldehyde (B).

BOH can of course release the hydroxyl radical to give B and free OH, which would again roam in the media. This requires 6.4 kcal mol^–1^ according to G4 calculations, with B + OH lying at −50.9 kcal mol^–1^ below TR + O_2_. Murakami et al.^57^ reported a value of −51.4 kcal mol^–1^ using the CBS-QB3 method, in good agreement with our calculations. On the other side, Sander et al.^60^ found both experimentally and theoretically that BOH can proceed toward a complex of benzoyl radical and water, our BZOw species in the mechanism shown in Scheme 3. The transition state we found for this transformation has a barrier of only 3.0 kcal mol^–1^ at the G4 level and the resulting BZOw complex is 24.7 kcal mol^–1^ more stable than BOH and 31.1 kcal mol^–1^ more stable than benzaldehyde plus the OH radical. This means that benzaldehyde detected in toluene oxidation cannot arise from this reaction channel. Notice that this is not a methodological artifact. Sander et al.^60^ found a barrier of 4.5 kcal mol^–1^ (a little higher than our value of 3.0 kcal mol^–1^) and a stabilization energy of 26.4 kcal mol^–1^ (in fair agreement with our value of 24.7 kcal mol^–1^). Notice also that the transition state is then below the energy necessary to decompose BOH but at the same time reasonably close to it. It may be hypothesized that if BOH is formed, then an equilibrium will exist between dissociation (and reassociation) along the B + H channel and the transformation to BZOw overcoming TS_BOH_BZOw. This is important because it contributes to the idea that even if all initial OH would have been consumed by its initial reaction with toluene, there is the possibility that it is regenerated via multiple channels after the reaction of TR with O_2_. In the case of the benzoyl radical, the presence of OH would lead immediately to benzoic acid, a species that can also be derived from other B channels, as will be described in the following section.

Benzoyl itself is a well-known radical that has been obtained isolated in Ar matrices^66^ by the thermal reaction of the Ph radical with CO. It is highly reactive and can either decompose back to the phenyl radical and carbon monoxide or react with oxygen to give the benzoylperoxy radical. Sebbar et al.^67^ studied this reaction and calculated several intermediates and transition states at the DFT level.

Since water is present in the reaction, being produced in the H-abstraction process by OH from toluene, it is reasonable to assume that species like BP could produce water complexes. We found two different complexes with one water molecule, BPw1 and BPw2 as sketched in channels B′ and B″. BPw1 is 39.0 kcal mol^–1^ more stable than isolated BP and water, thus suggesting that, provided this channel is open, it will end with the formation of this complex rather than isolated BP. Even if the entropic factor rules against the complex, its free energy is 7.4 kcal mol^–1^ lower than the isolated species. BPw1 is a complex where the water-peroxide group is exposed to the environment, while BPw2 has an extra interaction of the water with a hydrogen of the ring. It is 12 kcal mol^–1^ less stable than BPw1, but the structure resembles more that of the isolated BP. Thus, it is conceivable that BP arises initially by the reaction between TR and O_2_, then it complexes with water to form BPw2 that lately rearranges to BPw1. The computed relative energies of the involved species firmly suggest that the reaction is strongly displaced toward BPw1. At any rate, both BPw1 and BPw2 will evolve toward benzoic acid in the end.

As can be seen in panel 5 of Figure 3, water serves to lower the energy of the initial BPw1 complex with respect to BP + H_2_O and also to lower the energies of the transition states needed to arrive to the complex of benzaldehyde with the hydroxyl radical and one water molecule BHOw. This complex swiftly rearranges to the complex of the benzoyl radical and two water molecules BZO2w through a TS that lies only 0.3 kcal mol^–1^ above BHOw. BZO2w itself lies 86.4 kcal mol^–1^ below the isolated BP and H_2_O, being thus very stable. Of this energy, 6.3 kcal mol^–1^ is due to the hydrogen bonding of the two waters, which may have to be released so that OH can easily attack the carbon of the benzoyl radical and form benzoic acid.

Panel 6 in Figure 3 shows all of the mechanisms we have discussed with a common reference, T + 2OH + O_2_. Only the most stable products have been highlighted, since all of the species were already shown in the previous panels. When put on the same scale, it is easy to appreciate that once TR is formed, the initial reaction with O_2_ in the presence of water is the most favorable path leading to the benzoyl radical through a series of intermediates. We have shown that OH is released to the environment in several of the channels, so that even if the initial OH would be completely consumed in the generation of the cresols or TR, it would be nonetheless available later through some of the reactions. In this way, it is obvious that benzoic acid would be the most probable product, given its very large stabilization energy. If OH is present in excess, then TR would react preferentially with this radical instead than with oxygen, and benzyl alcohol would be readily obtained. Since all of the transition states (except those in channels A2, C, F3, and G) are submerged, all reactions could eventually occur. Thus, under different reaction conditions, the cyclic ketones (cyclohexadienone and quinone) and polyhydroxylated products (like catechol) might be obtained besides benzoic acid. What is not clear from this study is whether benzaldehyde would be obtained at all from the reaction of TR with oxygen, a conclusion that is largely taken for granted, at least after Clothier’s work.^56^ We do not have yet a plausible explanation for this result of the calculations. It is clear from the energy difference between the dissociation of BOH in B + OH and the height of the transition state TS_BOH_BZO2w that a complex equilibrium would be established where OH would be produced and also consumed to finally produce benzoic acid. Most of the theoretical work we are aware of disregards the conversion of the BOH complex to BZOw through a very low lying transition state (except Sander et al.^60^). Formation of the benzoyl radical (BZO) is a very favorable reaction path, especially if catalyzed by water molecules, as can be seen in channels B and B′. Even if no extra OH is available for converting it to benzoic acid, the dimerization of this radical would give rise to dibenzo glyoxal that should be observable together with benzaldehyde.

## Conclusions

An extensive investigation of several reaction channels on the potential energy surface of the oxidation of toluene by OH in the presence of oxygen has been performed using CBS-QB3, G4, and M06/cc-pVQZ levels of theory. More than 120 different species were found, including reactants, intermediates, transition states, and products, and several novel reaction paths were explored. The reliability of the chosen level of theory is supported by previous research on the formation enthalpies of several of the radical and closed-shell species on these reaction paths^47^ as well as on an in-depth study of the initial attack of the hydroxyl radical to toluene.^37^

The results lead to some interesting conclusions on both reaction channels that are well studied for this reaction: addition of OH to the ring, to give the hydroxy cyclohexadienyl radical, which later on leads to cresols, and the abstraction path leading to the benzyl radical plus water. We were able to show that if OH is present in excess, the route through the hydroxy cyclohexadienyl radical leads directly to the dihydroxy species, while the route through TR leads to benzyl alcohol and *o*-cresol. This path, which has never been considered before, explains the larger amount of *o*-cresol with respect to benzaldehyde, since we showed in our previous work^37^ that the abstraction reaction would be slightly more favorable than addition even at low temperatures. If, instead, OH is not in excess, the cresol arises from the hydroxy cyclohexadienyl radical through a less favorable (i.e., slower) route involving the usual reaction with oxygen.

We were also able to show that the channel leading to *o*-cresol from TR in the presence of a secondary hydroxyl radical species is close in the case of *p*-cresol (also *m*-cresol, but these calculations were not shown here). This explains, in our opinion, the larger amount of *o*-cresol with respect to *p*- and *m*-cresol. This is an experimentally testable prediction. Since only the production of the *p-*cresol is affected, the ratio of concentrations *o-*cresol/*p-*cresol should increase with the increment of OH concentration. An experiment in a reaction chamber that measures the amounts of *o*-cresol in relation to *p*- and *m*-cresol (this last one is not shown in this paper, but it is similar to para) would confirm or disprove the suggested mechanisms.

We explored finally the details of the “benzaldehyde” pathway of the benzyl radical. In this case, we found a large number of open channels that lead essentially to cyclic ketones, polyhydroxylated species, and foremost to benzoic acid. The most important finding in this mechanism was that the benzaldehyde–OH complex formed, BOH, after internal 1,3-hydrogen shift and OO bond breaking, has less propensity to dissociate to B + OH than to react to the benzoyl–H_2_O complex (BZOw) or in the presence of an ancillary water molecule to BZO2w because of the very low transition states involved. This would mean that not benzaldehyde but benzoic acid would be produced in the process. Our results are admittedly sketchy in many senses and more a proof of concept than a full-fledged complete mechanism that could be used for kinetic assessment. We believe, however, that we have provided evidence of some more complicated reaction paths than hitherto assumed playing a role in the oxidation of toluene. We look forward to more extensive theoretical studies of the individual reaction paths, although the information available to compare with shows that the methods used are in agreement with more sophisticated ones.

From a different point of view, we note that our proposed mechanisms are amenable to experimental testing, since basically the proposal is that different mechanisms come into play depending on the [OH]/[O_2_] concentration ratio. It would be interesting to see some of these experiments being performed in the near future.

## Supporting Information Available

Structure of the minima and transition state species studied (Figure S1); M06/cc-pVQZ XYZ coordinates (Table S1); absolute *E* + ZPE energies (Tables S2–S5); absolute and relative energies of the isomers of BP (Table S6) and reaction coordinate energy profile for the addition (Figure S2); full citation of Gaussian 16, ref^45^, 64 p (PDF)
