# Supplementary material for: Reinvestigation of the Deceptively Simple Reaction of Toluene with OH and the Fate of the Benzyl Radical: The “Hidden” Routes to Cresols and Benzaldehyde
Source: J Phys Chem A. 2020 Jun 16;124(28):5917–30. doi: 10.1021/acs.jpca.0c03727 (PMC8008427; doi:10.1021/acs.jpca.0c03727)
Supplement: Supplementary file 1 — jp0c03727_si_001.pdf [file jp0c03727_si_001.pdf]

A Reinvestigation of the Deceptively Simple Reaction of Toluene with OH, and the Fate of the Benzyl Radical. The "Hidden" Routes to Cresols and Benzaldehyde.

Zoi Salta[1*], Agnie M. Kosmas[2], Marc E. Segovia[3], Martina Kieninger[3], Nicola Tasinato[1], Vincenzo Barone[1*] and Oscar N. Ventura[3*]

[1]*Scuola Normale Superiore, piazza dei Cavalieri 7, 56126 Pisa, Italy*
[2]*Physical Chemistry Sector, Department of Chemistry, University of Ioannina, 45110 Ioannina, Greece*
[3]*Computational Chemistry and Biology Group, CCBG, DETEMA, Facultad de Química, Universidad de la República, 11400 Montevideo, Uruguay*

SUPPORTING INFORMATION

Structure of the minima and transition state species studied in this work (Fig. S1)

M06/cc-pVQZ XYZ coordinates of the species studied in this work (Table S1)

Absolute E+ZPE energies of the species studied in this work (Tables S2-S5)

Table S2. Species included in Fig. 1 in the main text. Δ (E+ZPE) in kcal mol^{-1}.

Table S3. Species included in Fig. 2 in the main text (some of the species are already included in Table S2). Δ(E+ZPE) in kcal mol^{-1}.

Table S4. Species included in Fig. 3 in the main text (some of the species are already included in Tables S2 or S3). Δ(E+ZPE) in kcal mol^{-1}.

Table S5. Some species corresponding to the *meta* addition are included in Scheme 2 in the main text. Δ(E+ZPE) in kcal mol^{-1}.

Absolute and relative energies of the isomers of BP and reaction coordinate energy profile for the addition (Table S6)

Energy profile for the addition of O_2 to TR. No transition state was found at any of the theoretical levels used in this work. Scan coordinate is the C-O distance in Å (Fig S2)

Full reference of Gaussian 16 (ref. (45) in the paper)

Fig. S1. Structure of the minima and transition state species studied in this work

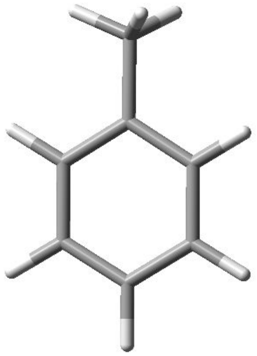

Toluene (T)

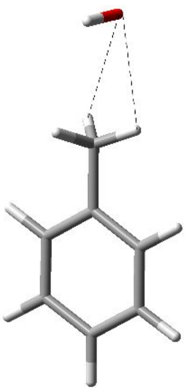

Pre-reactive Complex (PRC_CH3)

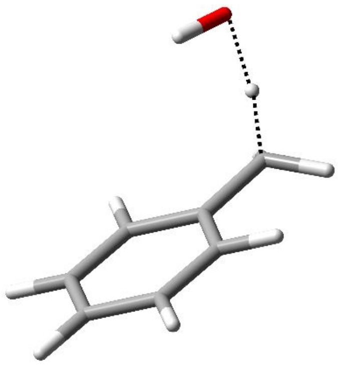

TS Abstraction (TS01_TR)

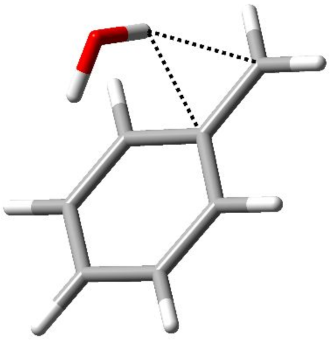

Post-reactive complex (HPrw)

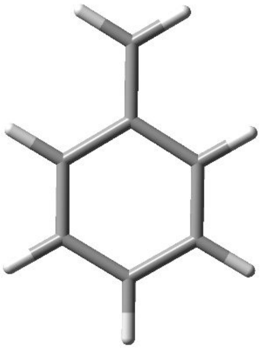

Benzyl Radical (TR)

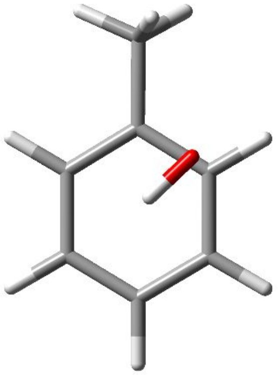

Pre-reactant o-Addition (M01_TR)

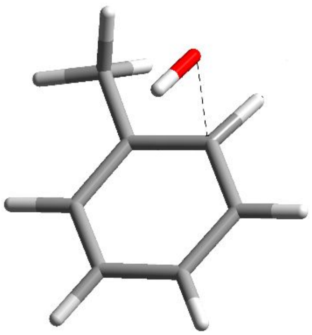

TS o-Addition (TS02_oC)

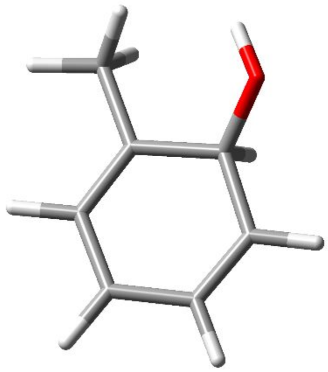

o-Addition (M03_oC)

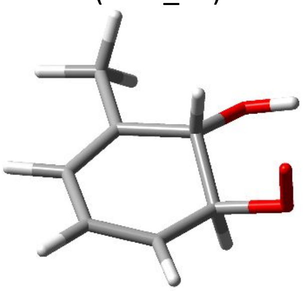

O2-p-Addition (M07_oC)

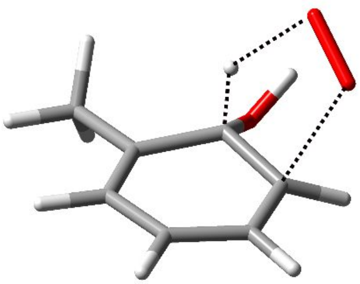

H-transfer (TS04_oC)

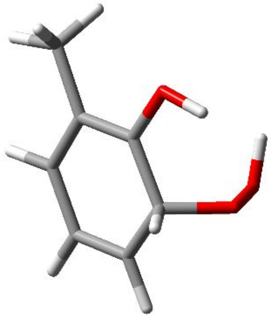

H-transferred (M08_oC)

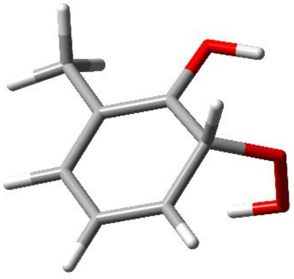

TS Rotation (TS95_oC)

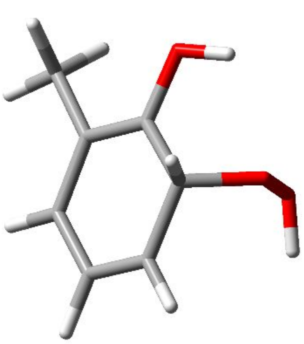

M09_oC

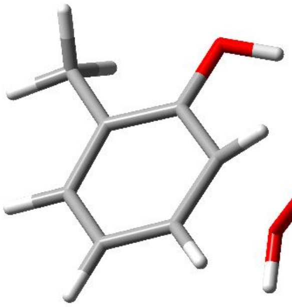

OOH release (TS06_oC)

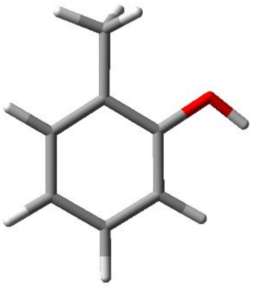

o-Cresol

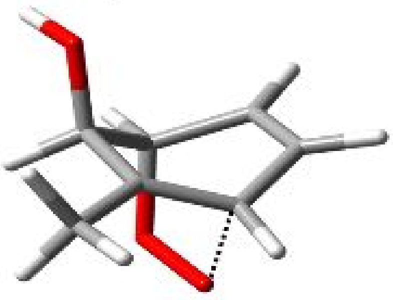

Cyclization (TS07_oC)

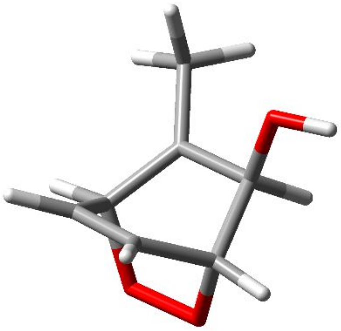

Hydroxylated Bicycle (M10_oC)

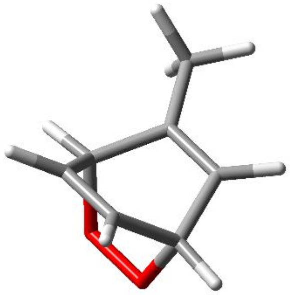

Bicyclic Peroxide (M11_oC)

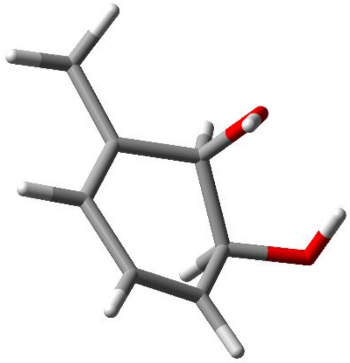
Methylcathecol (M16_oC)

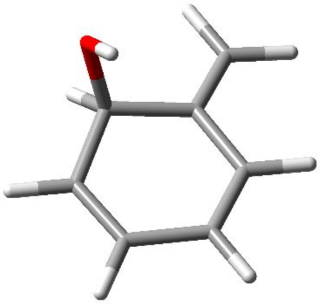
OH-Addition (M12_oC)

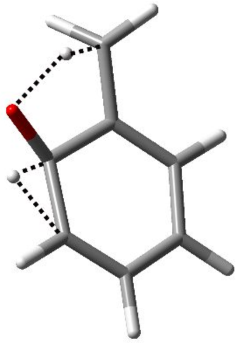
H-transfer (TS08_oC)

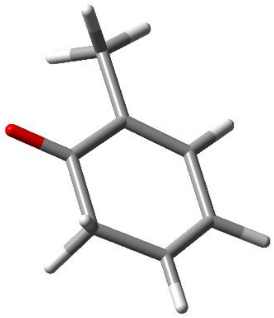
Dienone (M13_oC)

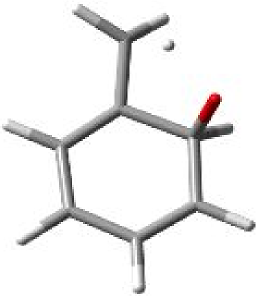
TS10_oC

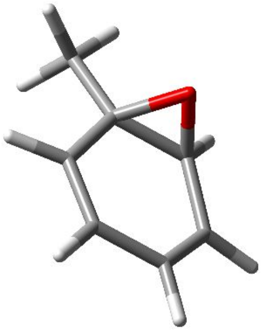
M14_OC

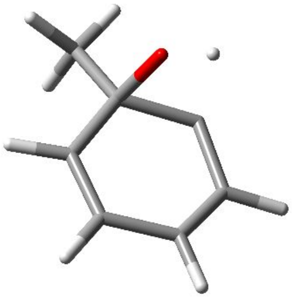
TS11_OC

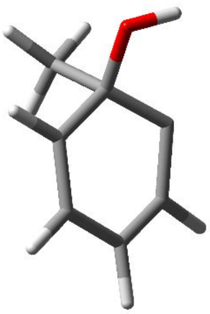
M15_OC

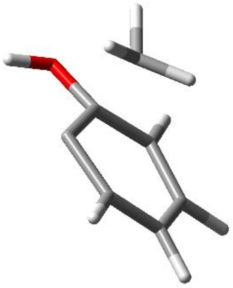
TS12_oC

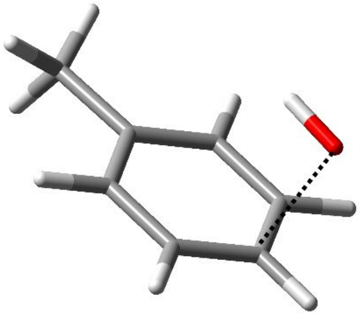

M17-pC

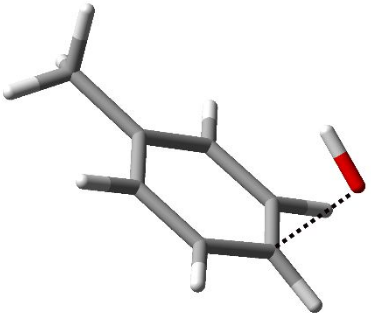

TS13_pC

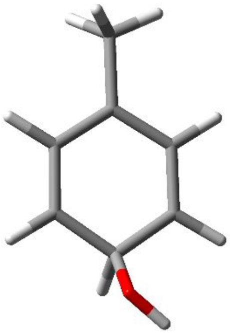

M18_pC

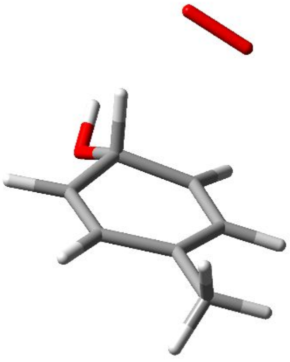

Pre-reactant (M19_pC)

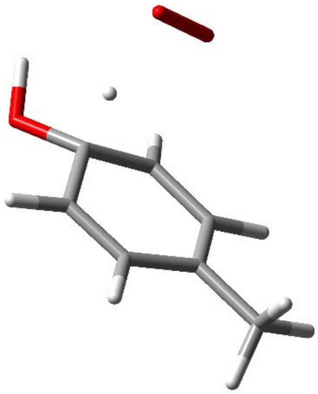

TS14_pC

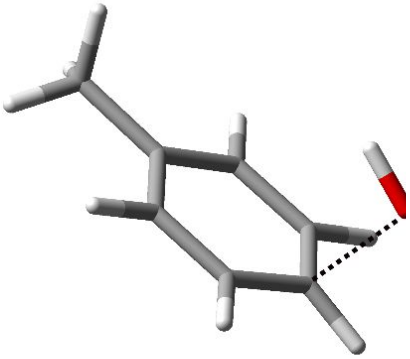

M20_pC

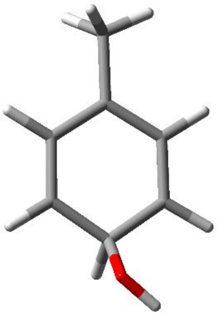

M21_pC

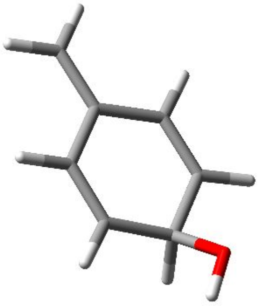

M22_pC

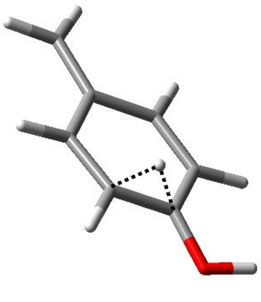

TS15_pC

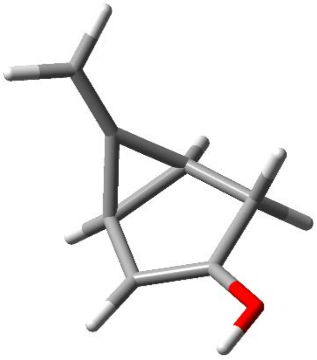

M23_pC

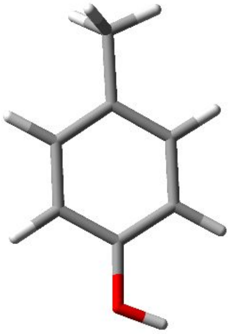

TS16_pC

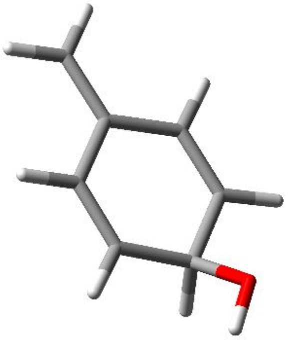

M24_pC

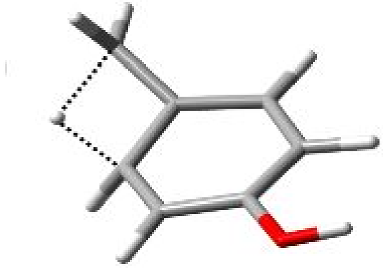

TS17_pC

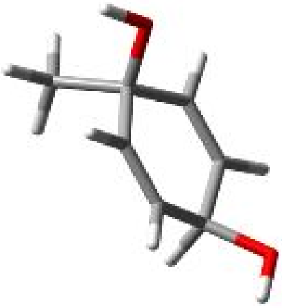

M25_pC

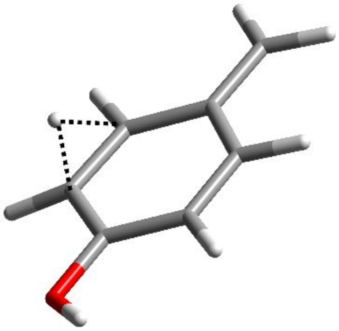

M26_pC

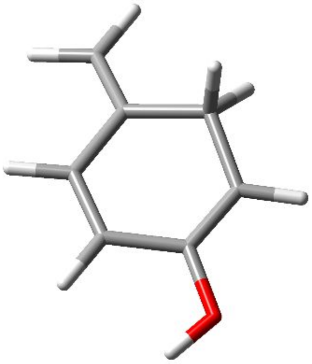

TS18_pC

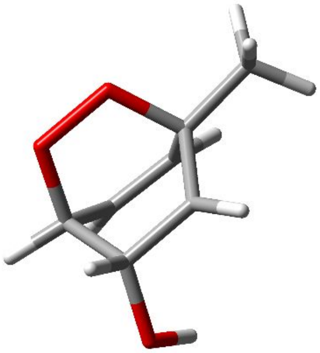

M27_pC

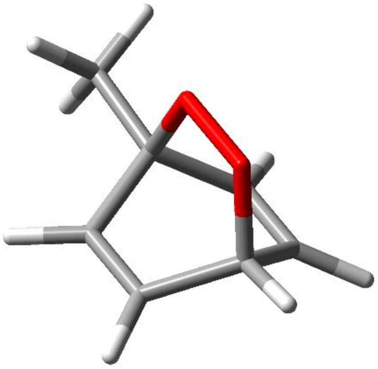

M28_pC

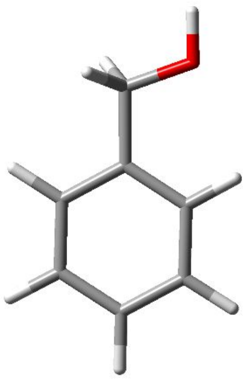

M29_TR

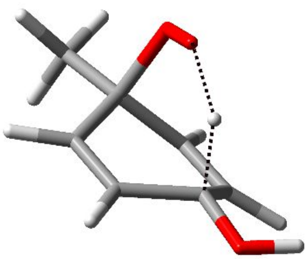

TS19_pC

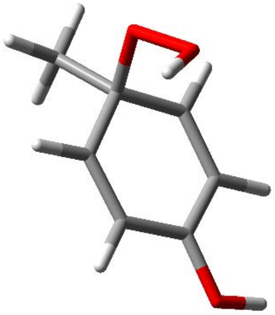

M30_pC

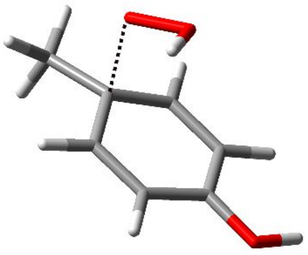

TS20_pC

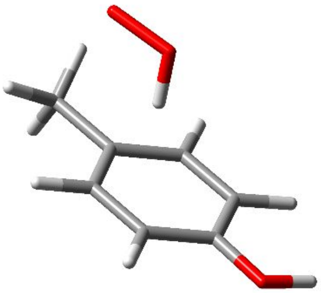

M31_pC

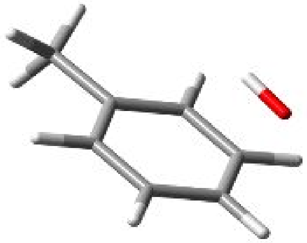

M32_mC

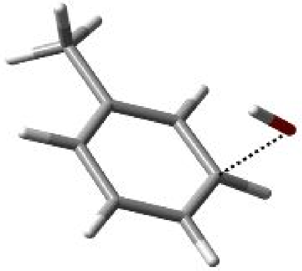

TS21_mC

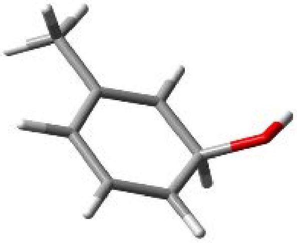

M33_mC

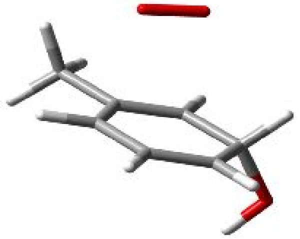

M34_mC

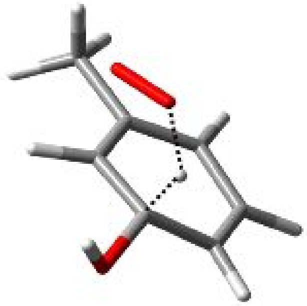

TS22_mC

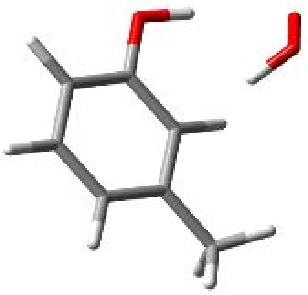

M35_mC

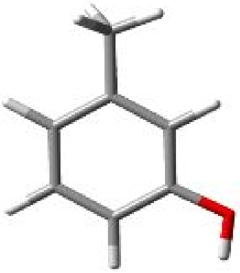

M36_mC (*m*-Cresol)

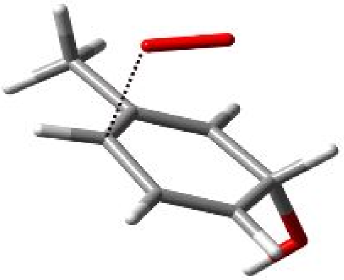

TS23_mC

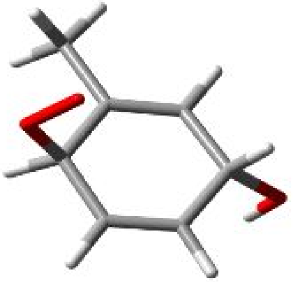

M37_mC

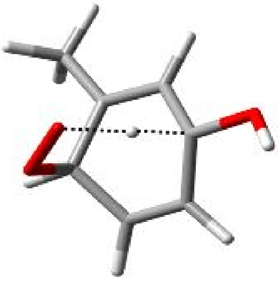

TS24_mC

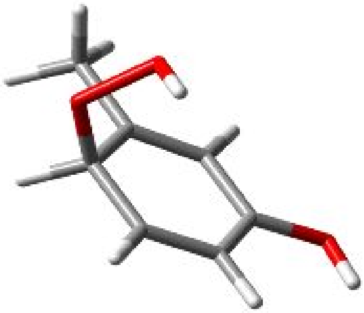

M40_mC

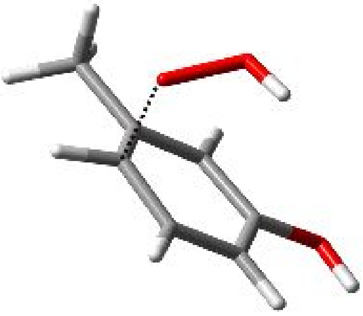

TS26_mC

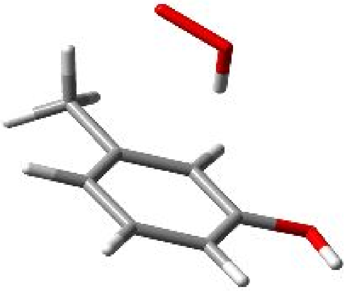

M41_mC

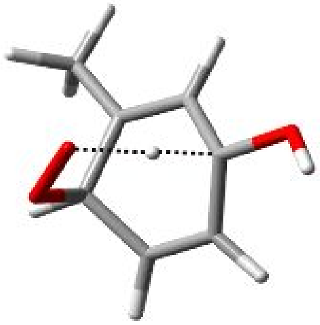

M42_mc

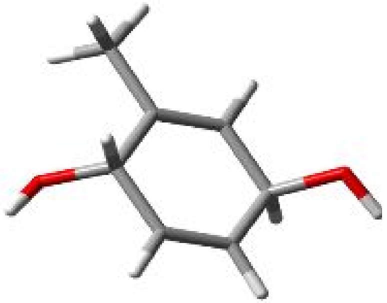

M43_mC

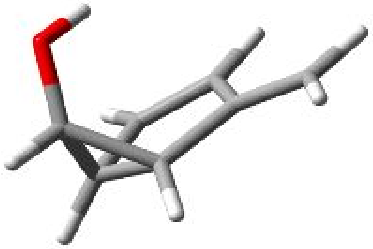

M46_mC

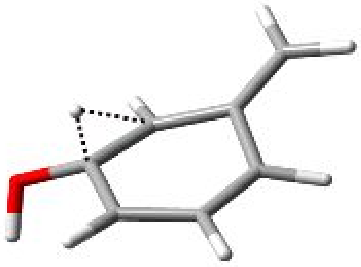

TS30_mC

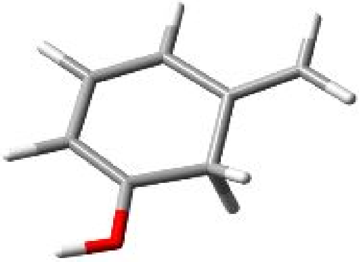

M47_mC

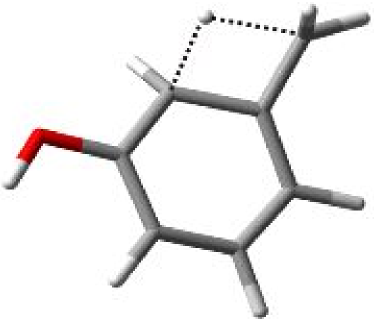

TS31_mC

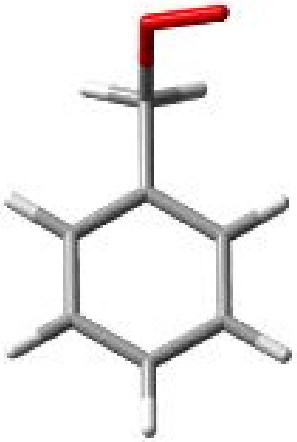

BP

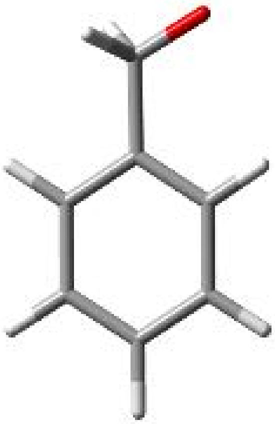

BO

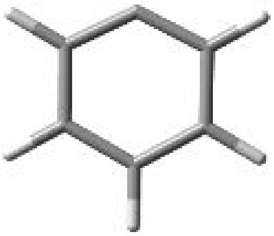

PhR

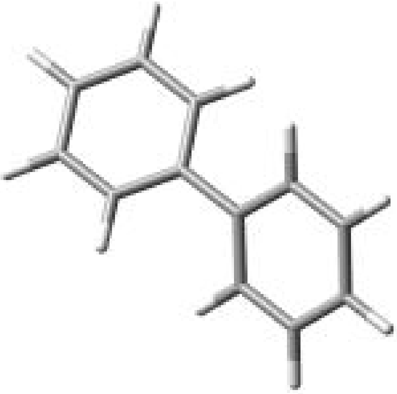

BPh

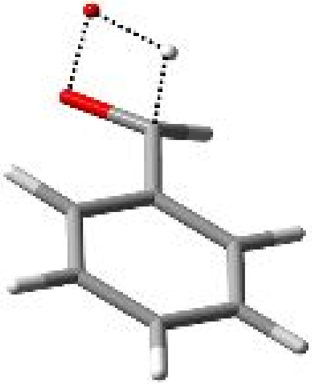

TS_BP_BHP

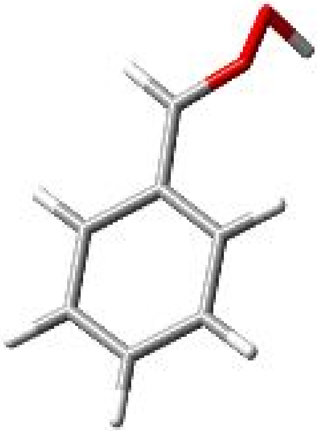

HPB

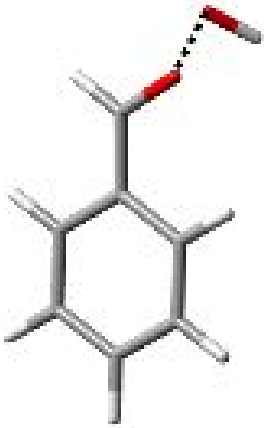

TS_BHP_BOH (1)

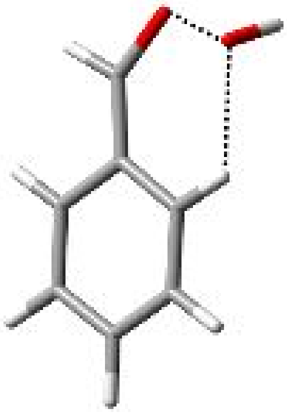

TS_BHP_BOH (2)

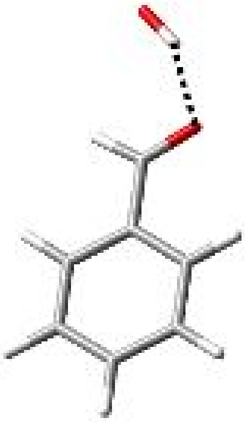

BOH

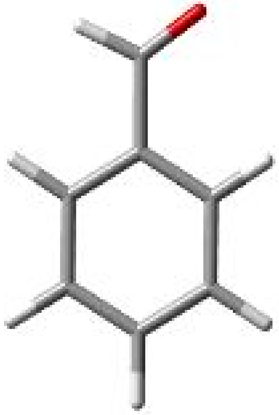

B

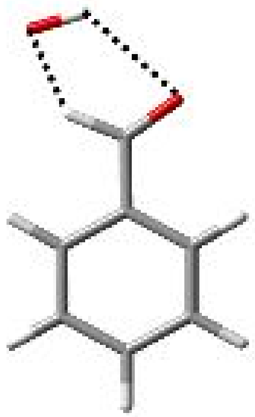

TS_BOH_BZOw

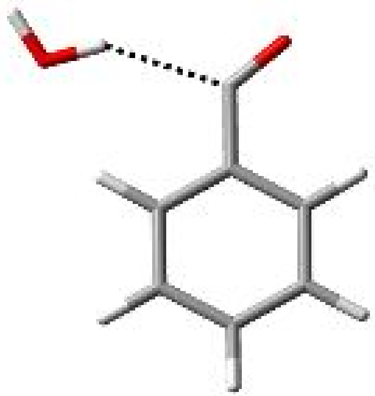

BZOw

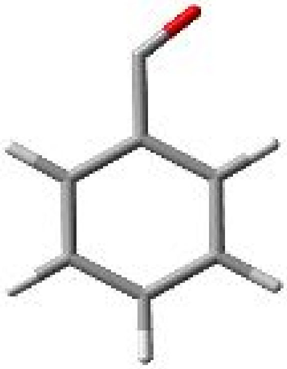

BZO

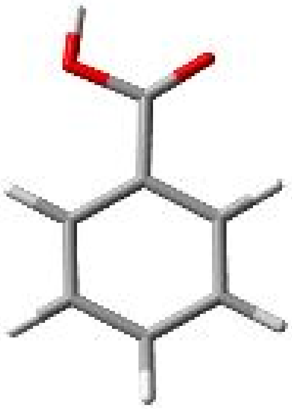

BZoic

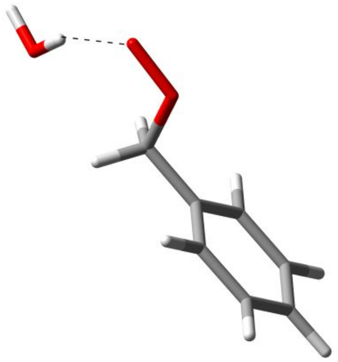

BPw1

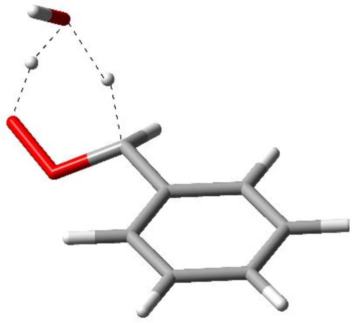

TS_BPw1_BHPw1

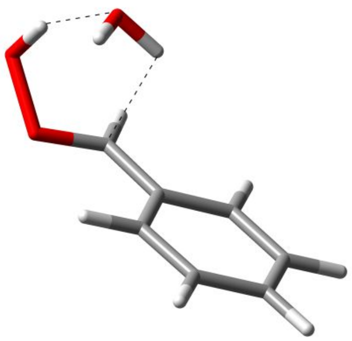

BHPw

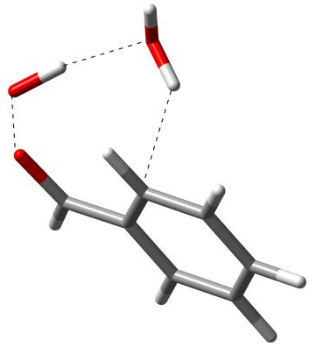

TSBHPwB

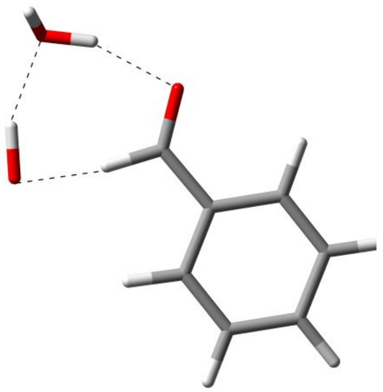

BOHw

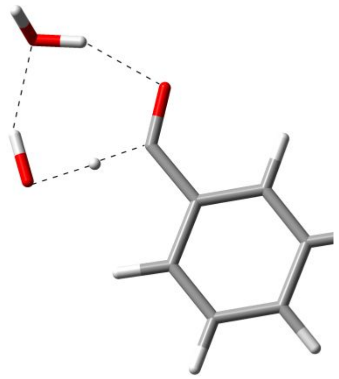

TS_BOHw_BZO2w

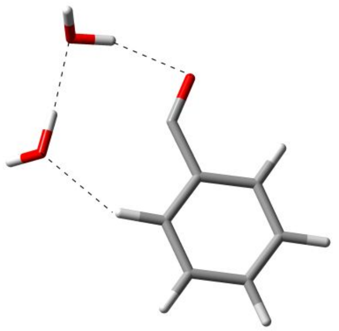

BZO2W

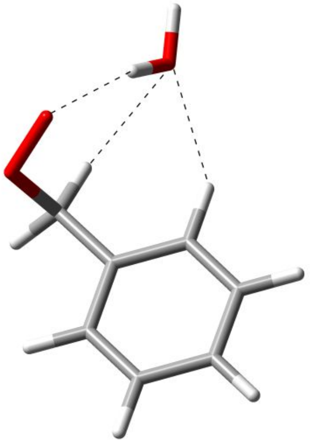

BPw2

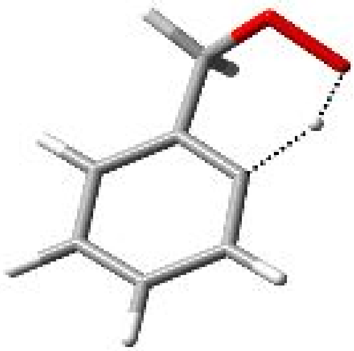

TS_BP_HPB

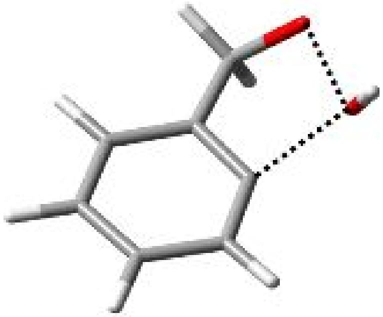

TS_HPB_HOBO

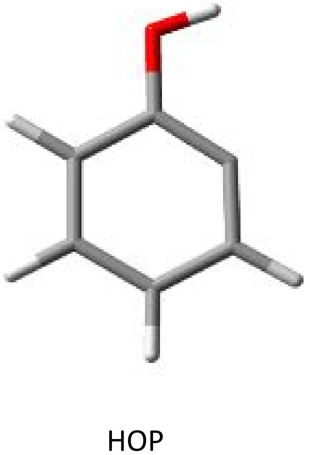

HOP

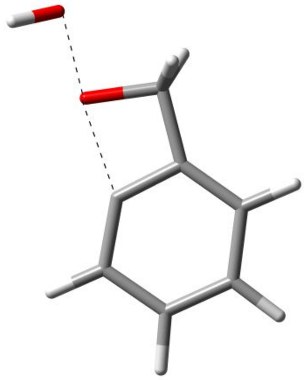

TS_HPB_BOEOH

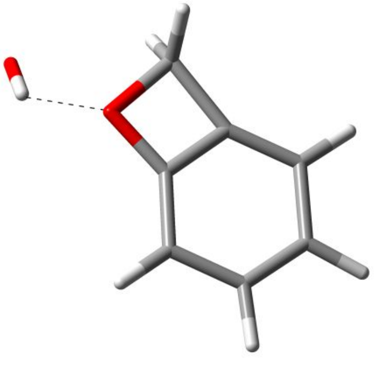

BOEOH

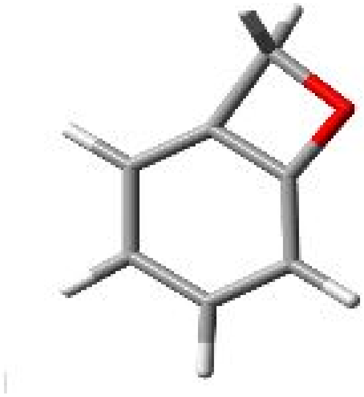

BOE

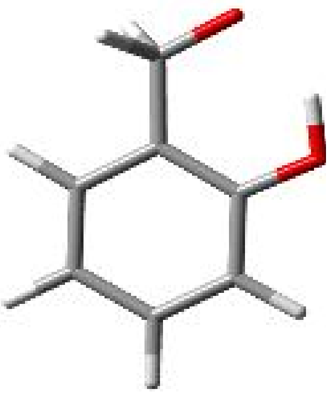

HOBO

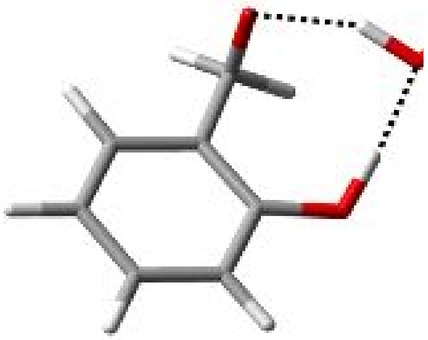

HOBOw

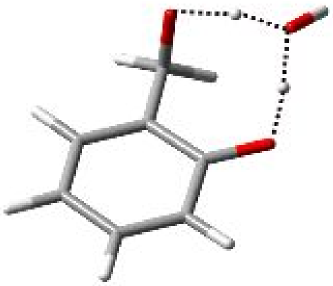

TS_HOBOw_oHOMCHDw

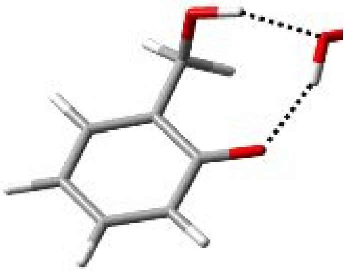

oHOMCHDw

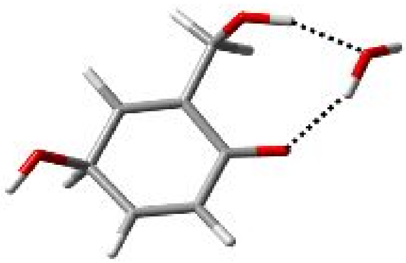

4OH2HOMCHDw

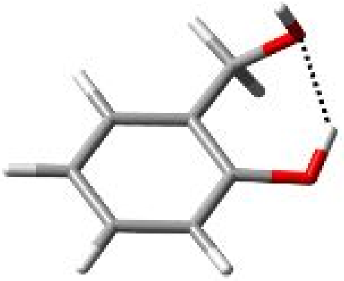

HOMP

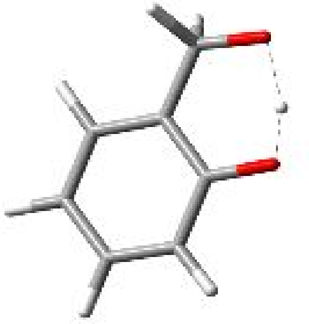

TSHOBOoHOMCHD

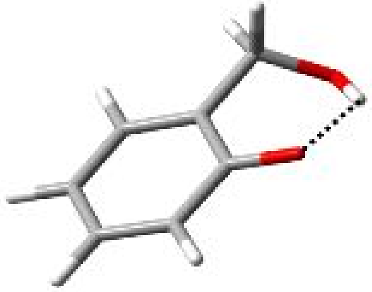

oHOMCHD

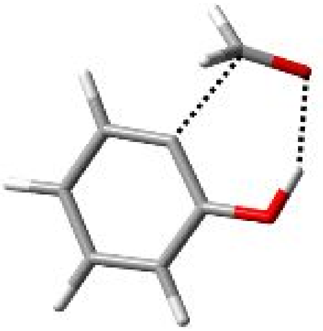

TS_HOBO_HOP

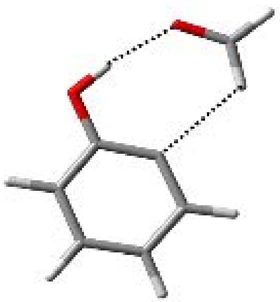

HOP.CH2O complex

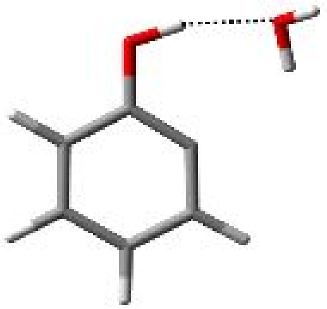

HOP.water complex

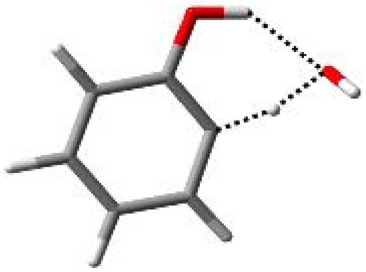

TS_HOPw_PhOH

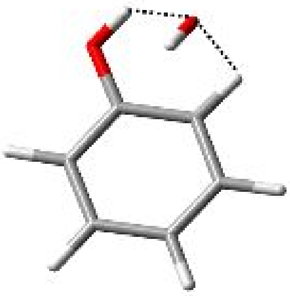

PhOH

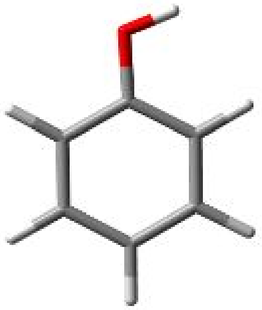

Ph

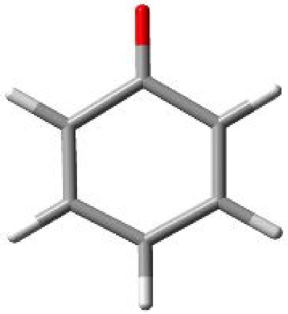

Phenoxy

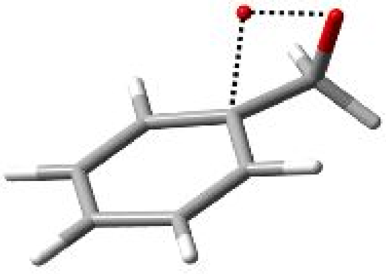

TS_BP_DOESB

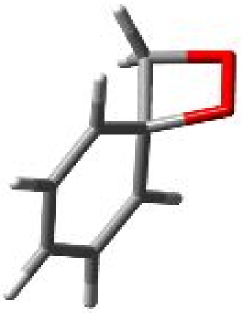

DOESB

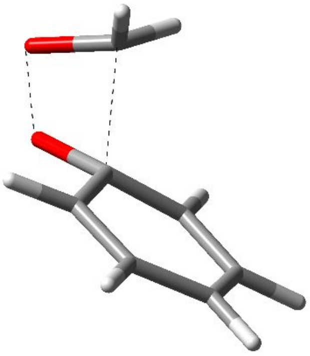

TS_DOESB_PhO

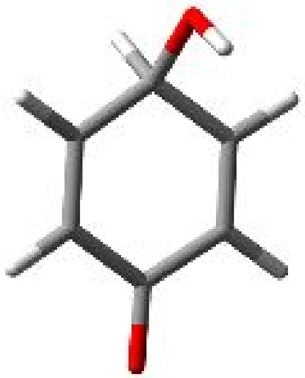

pOHCHD

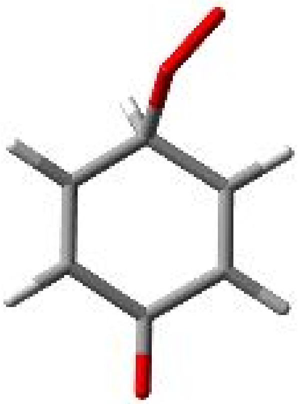

pPoxCHD

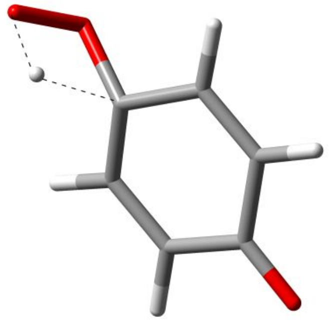

TS_pPoxCHD_pHPoxCHD

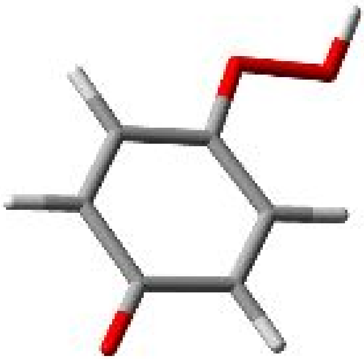

pHPoxCHD

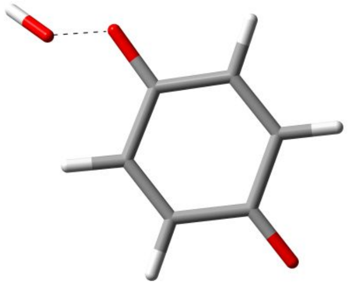

TS_pHPoxCHD_pBQ

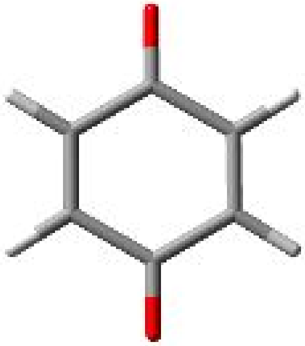

pBQ

Table S1. M06/cc-pVQZ XYZ coordinates of the species studied in this work

Geometries of all the species reported in Fig.1

- **M01_TR**

C	-1.99568700	0.07388600	-0.10792400
C	-1.12753300	0.90930600	-0.78599300
C	-1.50509700	-1.07363900	0.49632900
C	0.22648300	0.60464500	-0.84829300
C	0.72953600	-0.55323900	-0.24774600
C	-0.15595700	-1.38241100	0.42383200
H	-3.04864700	0.31070000	-0.05207600
H	-1.49352600	1.80905700	-1.25985200
H	-2.17807300	-1.73396800	1.02590400
H	0.89972900	1.24823400	-1.39789400
H	0.21714000	-2.28050400	0.89916200
O	0.93838200	1.92942000	1.05727400
H	0.19839600	1.55812200	1.55719500
C	2.19082300	-0.84699100	-0.30734500
H	2.55720100	-0.82130000	-1.33351500
H	2.74321700	-0.09164200	0.25270100
H	2.42209700	-1.82340200	0.11301800

- **M02_TR**

C	-1.99024300	-0.44550200	0.02522700
C	-1.41240200	0.06427000	1.18473000
C	-1.33612900	-0.29548800	-1.19469600
C	-0.20079700	0.70981200	1.13285100
C	0.48865800	0.86940700	-0.09405300
C	-0.12379500	0.34774100	-1.25991700
H	-2.94309400	-0.95308000	0.07142800
H	-1.91944900	-0.05045300	2.13264800
H	-1.78433200	-0.68903200	-2.09643200
H	0.25073300	1.09958700	2.03587700
C	1.73721100	1.48775700	-0.14798800
H	0.38762000	0.45701600	-2.20745100
H	2.26081700	1.60248900	-1.08625200
H	2.20196300	1.88152900	0.74449900
H	2.10530500	-1.12416500	0.19676000
O	1.93539300	-2.06548600	0.26630400
H	0.98227000	-2.12799100	0.20157500

- **M03_oC**

C	2.01482000	0.29457600	0.01499300
C	1.04286800	1.20000300	0.26841100
C	1.70046500	-1.06443000	-0.17766700
C	-0.39664800	0.82854000	0.35535900

C	-0.66360000	-0.62110700	0.10211800
C	0.36074000	-1.48360700	-0.13076100
H	3.04682500	0.61376100	-0.04362200
H	1.28614000	2.24563800	0.41827300
H	2.48502400	-1.78047400	-0.37504400
H	-0.76314600	1.05523900	1.37488900
H	0.13129300	-2.52969700	-0.29472200
O	-1.19416500	1.57912700	-0.56488200
H	-1.06401300	2.51132000	-0.38750200
C	-2.08679700	-1.04248100	0.13336000
H	-2.56590200	-0.72609400	1.06383000
H	-2.64846300	-0.56947500	-0.67338400
H	-2.18553100	-2.12218700	0.04146700

- **M04_oC**

C	-0.36234000	2.00321200	0.41684900
C	0.40364000	0.92465400	0.72078100
C	-1.59082000	1.84012300	-0.23938800
C	0.02663300	-0.45073200	0.30582300
C	-1.35880400	-0.56872100	-0.23401700
C	-2.07135500	0.55098000	-0.52740600
H	-0.04293900	2.99329500	0.71219900
H	1.31816900	1.02946200	1.28889500
H	-2.19567500	2.70355300	-0.47699900
H	0.69597800	-0.74202000	-0.53519400
H	-3.05071100	0.44306900	-0.97713000
O	0.22380900	-1.40317000	1.34249200
H	1.15846500	-1.60779100	1.38758300
C	-1.85884300	-1.94043000	-0.49737900
H	-1.14111200	-2.50734100	-1.09605200
H	-1.97770100	-2.49494800	0.43402200
H	-2.81282600	-1.92172800	-1.01992200
O	2.89316600	-0.64753300	-0.69969600
O	2.99798600	0.54444500	-0.56641900

- **M05_oC**

C	0.56453900	1.97332700	-0.38934000
C	-0.26612900	0.91318500	-0.72709700
C	1.79170400	1.72815600	0.19166700
C	0.13068700	-0.39472000	-0.47103600
C	1.37602900	-0.66016300	0.10607000
C	2.18365400	0.41732200	0.42460800
H	0.24823200	2.98582700	-0.59750200
H	-1.20463500	1.09005700	-1.24026000
H	2.44617200	2.54573900	0.45622600
H	-1.80451100	0.66161000	0.83298200
H	3.14851700	0.22045200	0.87517500
O	-0.65153800	-1.45383200	-0.76736200
H	-1.55589000	-1.15280500	-0.92216000

C	1.79137500	-2.07104700	0.35857800
H	1.07905100	-2.58150400	1.00677700
H	1.82642600	-2.64394400	-0.56819800
H	2.77355600	-2.10881100	0.82427000
O	-2.73203500	0.40770700	1.04719800
O	-3.16493600	-0.26049800	0.01666200

- **M07_oC**

C	1.51138100	0.03119500	0.09388200
C	1.51834600	1.36519700	0.05584300
C	0.18508500	-0.67918100	0.15713300
C	-0.88631500	0.09436400	-0.58020600
C	0.28166100	2.13165900	0.01426100
C	-0.88141200	1.54400900	-0.24810700
C	2.73405500	-0.80237900	0.21093900
H	2.45962500	1.89766100	0.09649100
H	-0.11471000	-0.70922800	1.21947900
O	-2.17958600	-0.52972300	-0.32867100
H	0.32600100	3.19831000	0.18650600
H	-1.81417900	2.08632600	-0.30717800
H	2.81336000	-1.49972800	-0.62263200
H	3.62627000	-0.18124000	0.24338900
H	2.69857100	-1.41574800	1.11373500
O	0.31525200	-1.98085200	-0.35446000
H	-0.75963800	-0.06744000	-1.65742200
H	-0.50615900	-2.45096500	-0.20496000
O	-2.57391000	-0.36031600	0.89689700

- **M08_oC**

C	-1.42380200	0.02330300	0.05249300
C	-1.36969600	1.41539000	0.21390200
C	-0.29927900	-0.61790700	-0.38309800
C	-0.18565600	2.13881700	-0.03988700
C	0.93787500	1.52840900	-0.47714500
C	0.97225300	0.07149500	-0.72836400
C	-2.66308000	-0.75528500	0.36005500
O	-0.29423400	-1.96351200	-0.51472000
O	2.08471300	-0.60383600	-0.09565800
O	2.01471400	-0.38846400	1.30459200
H	-2.25136100	1.93420200	0.56519300
H	-0.18135000	3.20742600	0.12653000
H	1.85170400	2.07599600	-0.65981500
H	1.22669600	-0.14118700	-1.78144100
H	-3.00896700	-1.31084500	-0.51140900
H	-2.48605400	-1.48926400	1.14636300
H	-3.46375500	-0.09450700	0.68441200
H	0.60441300	-2.24461700	-0.71502700
H	1.45544100	-1.11603500	1.60374900

- **M09_oC**

C	-1.42891400	0.01706600	0.04289600
C	-1.38306400	1.40739700	0.20632700
C	-0.29530000	-0.62435500	-0.37427600
C	-0.19908000	2.13625900	-0.03009500
C	0.93065300	1.53150400	-0.46523600
C	0.96790400	0.07541500	-0.73088200
C	-2.66138200	-0.77015100	0.35326200
O	-0.27877800	-1.96195800	-0.49571900
O	2.09457600	-0.58996500	-0.11383500
O	1.92556100	-0.55742000	1.29493000
H	-2.26901400	1.92254500	0.55200400
H	-0.20288500	3.20547400	0.13456600
H	1.83267600	2.09176600	-0.67343400
H	1.21006000	-0.12692000	-1.78811400
H	-3.00082600	-1.33604900	-0.51397600
H	-2.47411600	-1.49552000	1.14492100
H	-3.46901300	-0.11491500	0.67183900
H	0.64095400	-2.24643700	-0.54031100
H	2.21638100	0.33599600	1.51751800

- **M10_oC**

C	-0.90467100	-0.61971000	-0.14808200
C	0.37313300	-1.25096700	0.27947400
C	-0.70834200	0.80160800	-0.52374400
C	0.90833100	-0.48097900	1.45047800
C	1.13320800	0.79004700	1.14625400
C	0.77936000	1.09419200	-0.27247600
C	-2.23965300	-1.23287200	-0.03348500
O	-1.57032700	1.62876800	0.23334700
O	1.33798000	-1.14549100	-0.78979000
O	1.51799900	0.23883200	-1.15189400
H	0.28938700	-2.32305200	0.44756000
H	-0.89473600	0.93662800	-1.59989500
H	1.11321700	-0.94480900	2.40379000
H	1.55653900	1.52659500	1.81148000
H	1.05347200	2.09789000	-0.59487800
H	-2.74714800	-0.93024100	0.88905500
H	-2.88898800	-0.91261400	-0.85121600
H	-2.18426200	-2.32111300	-0.04067400
H	-1.63089200	2.48593200	-0.18904100

- **M11_oC**

C	1.16572400	0.15127000	-0.18698500
C	0.26494400	-0.99708400	0.19747100
C	0.45353500	1.24001500	-0.43668600
C	-0.78813500	-1.24847500	-0.84274200
C	-1.48986400	-0.15536000	-1.09112500

C	-1.01752800	1.00331500	-0.25710900
C	2.63917800	0.01154300	-0.12867600
O	-0.43334700	-0.57478500	1.39920400
O	-1.20874000	0.59578600	1.12760800
H	0.80603300	-1.88527300	0.51799500
H	-0.98922500	-2.23403700	-1.23339900
H	-2.36330900	-0.09307500	-1.72183400
H	-1.62973000	1.89651500	-0.34266600
H	2.98875300	-0.77122500	-0.80503700
H	3.13741600	0.94188100	-0.39374600
H	2.95798300	-0.27430000	0.87567800
H	0.86165500	2.22016800	-0.63637900

- **M12_oC**

C	1.91223200	-0.15182900	-0.17249400
C	1.03143800	-1.09928000	-0.49250700
C	1.48436800	1.16525300	0.25801000
C	-0.44052200	-0.89241400	-0.33449600
C	-0.83193100	0.55940200	-0.15233600
C	0.18727100	1.49713700	0.26685400
H	2.97341600	-0.35501500	-0.22837100
H	1.35131400	-2.08724100	-0.79451600
H	2.23496300	1.88408100	0.55551200
H	-0.97482900	-1.29702500	-1.19593100
C	-2.10004300	0.93035800	-0.32742800
H	-0.13183300	2.48792200	0.56490300
H	-2.85189400	0.22532700	-0.65534300
H	-2.42111600	1.94563800	-0.13687200
O	-0.90344400	-1.68038300	0.75983300
H	-0.40935600	-1.41238200	1.53834100

- **M13_oC**

C	-2.04341200	0.15112300	0.00000000
C	-1.05624600	1.25648700	0.00000000
C	-1.65097300	-1.12112400	0.00000000
C	0.40748500	0.87271900	-0.00000100
C	0.74477400	-0.55962500	0.00000000
C	-0.24667600	-1.46733400	0.00000000
H	-3.09439700	0.40803800	0.00000100
H	-1.21301000	1.91311100	0.86119900
H	-2.37709200	-1.92198800	0.00000000
H	-1.21301100	1.91311300	-0.86119600
C	2.18933200	-0.91029800	0.00000000
H	0.01181600	-2.52049800	0.00000000
H	2.68997200	-0.48423900	-0.86967600
H	2.33426600	-1.98842700	0.00000000
O	1.26322200	1.72918100	0.00000000
H	2.68997100	-0.48423900	0.86967700

- **M14_oC**

C	-1.99008100	0.03419000	-0.10790900
C	-1.29339900	1.27779200	0.09093400
C	-1.32498100	-1.10635200	-0.34030200
C	0.04451000	1.34433000	0.05662000
C	0.86801100	0.13493900	0.00667100
C	0.12468300	-1.15813600	-0.19567100
H	-3.06914800	0.05469100	-0.17627200
H	-1.87584900	2.18615700	0.16351700
H	-1.84522500	-1.99844800	-0.66065100
H	0.55259400	2.30049600	0.03683200
C	2.29219600	0.27348400	-0.43166700
H	0.65728500	-1.97823300	-0.66885800
H	2.84865800	0.88284600	0.28104000
H	2.77031900	-0.70210800	-0.49012400
H	2.35290400	0.75367600	-1.40746700
O	0.66035400	-0.78757000	1.05624200

- **M15_oC**

C	2.00419200	-0.06272900	-0.03891800
C	1.34588700	1.20631900	0.01647600
C	1.31173100	-1.24486000	-0.05389800
C	0.00948000	1.25816300	0.00307500
C	-0.77614800	-0.00111500	-0.12221600
C	-0.04532300	-1.22616200	-0.40611200
H	3.08752700	-0.07348500	-0.08734200
H	1.94145500	2.10206300	0.12267900
H	1.86267400	-2.17856600	-0.04126900
H	-0.53899700	2.17453900	0.18532600
C	-1.34511200	-0.27389800	1.32128800
H	-1.99610500	-0.64480700	-1.42836500
H	-2.01463700	0.55524800	1.53962700
H	-1.91965400	-1.19517000	1.31696900
H	-0.55814300	-0.32918300	2.07068200
O	-1.86154400	0.20688200	-0.99956000

- **M16_oC**

C	-1.28130800	0.01214700	-0.19502600
C	-1.16161000	1.31814400	0.05969200
C	-0.04095200	-0.84176800	-0.17086200
C	0.13403700	1.93916000	0.30391300
C	1.26028500	1.28096800	0.04052700
C	1.20110500	-0.07262200	-0.59784900
C	-2.57725200	-0.69601100	-0.35963000
O	0.13802500	-1.40225400	1.12738800
O	2.35715800	-0.83611200	-0.38000600
H	-2.05100700	1.93269500	0.13165300

H	0.15752200	2.94384400	0.70405600
H	2.23970900	1.70547800	0.21370100
H	-2.64978600	-1.15565900	-1.34757800
H	-2.65862200	-1.50805500	0.36643700
H	-3.42561000	-0.02717800	-0.22947900
H	0.03432300	-0.69219800	1.76642000
H	2.21730500	-1.31018500	0.44558200
H	-0.15032100	-1.70407700	-0.83144000
H	1.11918600	0.08215600	-1.68299400

- **TS01_TR**

C	2.30136100	0.00003800	0.44980500
C	1.65183800	-1.19841900	0.20050800
C	1.65180300	1.19845800	0.20042100
C	0.36207800	-1.19773200	-0.29459200
C	-0.30222000	-0.00003700	-0.55530100
C	0.36204300	1.19769700	-0.29467900
H	3.31107800	0.00006700	0.83545100
H	2.15487200	-2.13606900	0.39148200
H	2.15480800	2.13613700	0.39132900
H	-0.14536900	-2.13411900	-0.48908100
C	-1.68960800	-0.00007500	-1.04197500
H	-0.14543300	2.13405400	-0.48923400
H	-1.96809400	0.89878600	-1.58692900
H	-1.96808000	-0.89900200	-1.58682600
H	-2.42938400	-0.00002500	-0.11625800
O	-3.08800700	0.00006000	1.12555500
H	-2.28411000	0.00010900	1.66050300

- **TS02_oC**

C	-1.98965700	0.04586400	-0.11930100
C	-1.12356300	0.97251600	-0.65282600
C	-1.49430600	-1.15812000	0.37031700
C	0.26201600	0.72878700	-0.65172700
C	0.75773900	-0.52242800	-0.21236900
C	-0.13340400	-1.43489600	0.31580400
H	-3.05104800	0.24685600	-0.08936200
H	-1.48905200	1.91197700	-1.04146100
H	-2.17299000	-1.88972700	0.78659100
H	0.91144500	1.36682100	-1.23351000
H	0.23717500	-2.38183500	0.68667400
O	0.82015600	1.83379400	0.94301900
H	0.18238000	1.47021000	1.56852300
C	2.22178500	-0.78071600	-0.27451200
H	2.59833600	-0.66848300	-1.29199200
H	2.74791700	-0.05183500	0.34359700
H	2.47093400	-1.78038800	0.07447000

- **TS03_oC**

C	0.28663000	-0.56493200	0.00000200
C	0.03167800	0.83195200	-0.00000200
C	-0.78423800	-1.45111100	0.00000300
C	-1.29469300	1.29564700	-0.00000400
C	-2.32389400	0.39465900	-0.00000300
C	-2.07712800	-0.98718600	0.00000000
C	1.69828300	-1.02931000	0.00000400
O	2.65674100	-0.09733000	0.00000300
O	1.03184100	1.66731000	-0.00000300
H	-0.58294900	-2.51609600	0.00000500
H	-1.46420000	2.36256900	-0.00000700
H	-3.34495600	0.74992900	-0.00000500
H	1.83046700	-1.73532100	0.85360600
H	1.83046900	-1.73532500	-0.85359400
H	1.90851700	1.05614200	-0.00000100
H	-2.90583100	-1.68005900	0.00000100

- **TS04_oC**

C	-1.40055900	-0.14265500	-0.17971800
C	-1.58147000	1.16755100	-0.44430900
C	-0.07905400	-0.59719100	0.31428900
C	0.81470600	0.44884200	0.83246500
C	-0.59869300	2.13790700	-0.10056800
C	0.54318200	1.79663900	0.56132500
C	-2.41408000	-1.18608300	-0.46927800
H	-2.50826700	1.49649600	-0.89572000
H	0.44286600	-0.91645800	-0.64237600
O	2.48128200	-0.01482400	-0.38403100
H	-0.80411700	3.17956800	-0.30685600
H	1.21653800	2.56010200	0.92353100
H	-2.72505200	-1.68668900	0.44768200
H	-3.28870900	-0.76013500	-0.95533300
H	-1.99704300	-1.96467500	-1.11154800
O	-0.16681600	-1.73747500	1.13813500
H	1.47056100	0.17834300	1.64654200
H	0.57499400	-2.30965100	0.93911700
O	2.17478900	-0.93857200	-1.14538900

- **TS05_oC**

C	-1.43850300	-0.00803400	0.04494400
C	-1.42334500	1.38668900	0.18518400
C	-0.28617800	-0.63109800	-0.35202300
C	-0.24422200	2.13309600	-0.02785500
C	0.90571100	1.54677400	-0.43273700
C	0.96529300	0.09417300	-0.72329600
C	-2.66150500	-0.81541200	0.34110500
O	-0.23531200	-1.96833100	-0.46129000
O	2.09965500	-0.57302200	-0.15200500

O	2.09659400	-0.45747000	1.27479500
H	-2.32519300	1.88931400	0.50701000
H	-0.26500500	3.20059400	0.14505000
H	1.81345400	2.11239300	-0.58929100
H	1.18026000	-0.08545500	-1.79061300
H	-2.98798300	-1.37415400	-0.53577600
H	-2.46931700	-1.54969100	1.12306100
H	-3.47951600	-0.17543400	0.66365900
H	0.69482400	-2.22268000	-0.51070200
H	1.24747300	-0.04143200	1.48365500

- **TS06_oC**

C	-1.34939000	0.13981000	0.07204300
C	-1.11823100	1.48804800	0.31019400
C	-0.35292600	-0.58119900	-0.56201700
C	0.06775300	2.11867200	-0.09197600
C	1.03443900	1.41300000	-0.74906700
C	0.88489400	0.01303300	-0.94038300
C	-2.60497500	-0.54195700	0.50443000
O	-0.53024800	-1.90057900	-0.75083700
O	2.04888800	-0.82411500	0.22297300
O	1.64926900	-0.61334200	1.52145100
H	-1.88014700	2.06224100	0.82143500
H	0.19616700	3.17603500	0.09224600
H	1.93553200	1.89192800	-1.10424600
H	1.42762000	-0.45632000	-1.75424000
H	-3.12798500	-0.98641400	-0.34205800
H	-2.38646600	-1.35506700	1.19663800
H	-3.27788700	0.15850100	0.99385200
H	0.33335400	-2.30851900	-0.86464700
H	2.06714700	0.22546300	1.75298000

- **TS07_oC**

C	-1.03577000	-0.38102300	0.25977100
C	-0.00578300	-1.05193500	0.90294100
C	-0.62807900	0.87901400	-0.43109900
C	1.11472700	-0.29543000	1.45513400
C	1.57292200	0.70522600	0.71120600
C	0.89968000	0.82563200	-0.60047500
C	-2.35438000	-0.97566800	-0.04279300
O	-1.08471700	2.00392500	0.28577100
O	0.92940700	-1.51680500	-0.72092700
O	1.12284700	-0.34469900	-1.42753100
H	-0.20340800	-2.03949200	1.30107500
H	-1.05632500	0.85791300	-1.44278800
H	1.54891700	-0.58929500	2.39973200
H	2.41458900	1.32261600	0.98380800
H	1.25931500	1.65416200	-1.20945900
H	-3.14870400	-0.24828300	0.13838200
H	-2.41814800	-1.25559200	-1.09900000

H	-2.55136600	-1.86204700	0.55642900
H	-0.96507200	2.78574700	-0.25480100

- **TS08_oC**

C	-1.93866600	-0.56797700	-0.09393600
C	-0.84527000	-1.35469500	-0.02511100
C	-1.80163000	0.84494000	-0.04349900
C	0.49702100	-0.78641300	0.25497500
C	0.57649900	0.69975100	0.13505400
C	-0.58804200	1.46969600	0.04096100
H	-2.92629900	-0.99413600	-0.20086800
H	-0.90770600	-2.43407100	-0.06878000
H	-2.69855000	1.44862800	-0.09967200
H	0.41216600	-0.86988000	1.39475900
C	1.91511700	1.07613900	-0.09913400
H	-0.52058500	2.54771700	-0.01946600
H	2.63839700	0.78679600	0.66136500
H	2.09099600	2.07229200	-0.48714400
O	1.61320200	-1.34685500	-0.19443100
H	2.11579400	-0.07115400	-0.64060400

- **TS09_oC**

C	2.02296500	0.10454900	0.01380800
C	1.06013700	1.13724200	0.20199000
C	1.58271900	-1.17211600	-0.14180600
C	-0.33188300	0.80238100	0.06753000
C	-0.78937500	-0.53731200	0.09860600
C	0.19620900	-1.47363700	-0.05947000
H	3.08061400	0.32570800	0.05593500
H	0.35561300	2.14909500	-0.43101000
H	2.28289100	-1.98218600	-0.28413200
H	1.26282400	1.87128100	0.98223900
C	-2.24792500	-0.83884000	0.10389000
H	-0.10322900	-2.51068000	-0.16462200
H	-2.73974100	-0.38471600	0.96414400
H	-2.42628100	-1.91183200	0.13065100
O	-0.99215900	1.84200700	-0.27235300
H	-2.73250900	-0.42633000	-0.78167200

- **TS10_oC**

C	1.94129400	-0.24407000	-0.04142400
C	1.54167300	1.10265900	-0.14972900
C	1.03644900	-1.19552700	0.29163000
C	0.23252500	1.51315100	-0.02292700
C	-0.74342900	0.58500100	0.29321900
C	-0.39242300	-0.87403500	0.43907100
H	2.97350700	-0.50157200	-0.23102300
H	2.29466200	1.84118400	-0.39505500
H	1.31199100	-2.23569200	0.38799200

H	-0.03940000	2.54210500	-0.21656300
C	-2.13283600	0.67364400	0.06650100
H	-0.83374500	-1.29844800	1.35893400
H	-2.53873200	1.58737700	-0.34805900
H	-1.93537900	-0.32618900	-0.71046000
H	-2.77975800	0.17782200	0.78567400
O	-0.91908200	-1.39394000	-0.73618600

- **TS11_oC**

C	-2.00144600	0.02233200	-0.10016500
C	-1.30357600	1.25701700	0.11554400
C	-1.34131900	-1.11705800	-0.39635100
C	0.03302200	1.29762200	0.08239800
C	0.84069600	0.06703900	-0.03175200
C	0.09053200	-1.19594300	-0.39331700
H	-3.08382100	0.02947900	-0.07143000
H	-1.87771100	2.16544900	0.23326300
H	-1.89139800	-2.01442800	-0.64818800
H	0.55878600	2.24444000	0.10875800
C	2.22565700	0.27139500	-0.56979200
H	0.79672900	-1.68397400	0.64375200
H	2.78712700	0.93668700	0.08727200
H	2.74441000	-0.68199000	-0.63554500
H	2.19074000	0.71854100	-1.56333600
O	0.81421700	-0.66607700	1.20075800

- **TS12_oC**

C	1.95132400	-0.08606400	-0.11452700
C	1.36259600	1.18045800	0.18590700
C	1.18491400	-1.16068000	-0.42375800
C	0.02632900	1.32227700	0.15473800
C	-0.78109200	0.16928000	-0.20671800
C	-0.24387400	-1.06919300	-0.61487200
H	3.03081700	-0.17190700	-0.09417200
H	1.99811800	2.01672600	0.44155900
H	1.67301200	-2.10544700	-0.63566200
H	-0.46838000	2.25601300	0.38590000
C	-0.91598100	-0.84134600	1.24042200
H	-2.36081600	-0.25427700	-1.11271600
H	-1.84447200	-0.37889900	1.55773400
H	-1.07873000	-1.90767700	1.14242500
H	-0.08102700	-0.61367800	1.89182800
O	-2.04672700	0.50884400	-0.61300600

- **TR**

C	0.00000300	-1.82349400	0.00000000
C	1.20277100	-1.12361900	0.00000000

C	-1.20276900	-1.12362000	0.00000000
C	1.20916300	0.25012600	0.00000000
C	0.00000000	0.98649500	0.00000000
C	-1.20916500	0.25012200	0.00000000
H	0.00000000	-2.90419700	0.00000000
H	2.13888500	-1.66494600	0.00000000
H	-2.13888100	-1.66495000	0.00000000
H	2.14588200	0.79260500	0.00000000
C	-0.00000300	2.37995400	0.00000000
H	-2.14588200	0.79260500	0.00000000
H	-0.92600600	2.93654200	0.00000000
H	0.92599500	2.93655100	0.00000000

- **BA**

C	2.28908900	0.31861600	0.00000100
C	1.35695000	1.33947600	-0.00000100
C	1.85969400	-0.99957100	0.00000100
C	0.00041000	1.05280500	-0.00000200
C	-0.43238900	-0.26236600	0.00000000
C	0.50836200	-1.28486600	0.00000100
H	3.34597000	0.54569400	0.00000200
H	1.68476000	2.36998400	-0.00000200
H	2.58097500	-1.80506000	0.00000200
H	-0.73003100	1.84812000	-0.00000300
C	-1.88714100	-0.62134700	-0.00000400
H	0.17331800	-2.31590200	0.00000200
H	-2.09649700	-1.24258900	-0.88052200
H	-2.09649800	-1.24260300	0.88050400
O	-2.67900300	0.53768900	0.00000500
H	-3.59983300	0.28436400	-0.00000100

- **oC**

C	1.97476800	0.49033600	0.00000000
C	0.85642200	1.30599400	0.00000000
C	1.82476400	-0.88296100	0.00000000
C	-0.41085100	0.74802500	0.00000000
C	-0.58595400	-0.63572400	0.00000000
C	0.55019100	-1.42757200	0.00000000
H	2.96046300	0.93412700	0.00000000
H	0.96337500	2.38473500	0.00000000
H	2.69113200	-1.52842800	0.00000000
H	0.42698900	-2.50351300	0.00000000
O	-1.53400800	1.51131300	0.00000000
H	-1.28863300	2.43712600	0.00000000
C	-1.96035000	-1.21795400	0.00000000
H	-2.52828600	-0.89523200	0.87293000
H	-2.52828700	-0.89523000	-0.87292800
H	-1.91863800	-2.30494400	-0.00000100

Geometries of all the species reported in Fig.2

- **M17_pC**

C	-0.60984600	-1.19568400	-0.18015000
C	-1.28362100	0.00000000	0.05010000
C	0.69556300	-1.19977300	-0.63198600
C	-0.60984200	1.19567800	-0.18017500
C	0.69556700	1.19975300	-0.63201400
C	1.36081400	-0.00001400	-0.85641000
H	-1.12017100	-2.13324200	0.00199200
H	1.21020700	-2.13611500	-0.79434400
H	-1.12016500	2.13324100	0.00194400
H	2.37655600	-0.00002000	-1.22120800
O	2.48335300	0.00000500	1.34978400
H	1.59577300	0.00017200	1.73460800
C	-2.70804400	0.00000800	0.50275500
H	-2.93862100	0.88285300	1.09658400
H	-3.38553900	-0.00000300	-0.35277900
H	-2.93862200	-0.88282300	1.09660700
H	1.21021300	2.13608900	-0.79439500

- **M18_pC**

C	1.50939200	-0.01473800	-0.36283500
C	0.68147700	-1.24335100	-0.24075900
C	0.70209300	1.23222400	-0.24309900
C	-0.65606100	-1.20253800	-0.07109100
C	-1.37175500	0.01762800	0.01808600
C	-0.63989500	1.21988400	-0.06150200
H	2.00300400	-0.02551200	-1.35091200
H	1.21331200	-2.18451000	-0.27771900
H	1.23962700	2.17157400	-0.29977000
H	-1.21153300	-2.12967200	0.01323200
H	-1.17575700	2.15736800	0.03008000
O	2.53182800	-0.09015300	0.63369800
H	3.13391200	0.64414700	0.50609400
C	-2.84703700	0.00777300	0.21319400
H	-3.12156400	-0.50545400	1.13762800
H	-3.25397000	1.01600800	0.25675700
H	-3.35093300	-0.52401900	-0.59693800

- **M19_pC**

C	-0.62681000	0.88862100	0.04359600
C	0.55209000	1.50134200	-0.61254900
C	-0.28459500	-0.32820000	0.82498900
C	1.74708700	0.87883800	-0.66461700
C	1.97726600	-0.36340000	-0.01852900
C	0.93898000	-0.91540600	0.74877200
H	-1.32043200	0.57968900	-0.76813900
H	0.39426100	2.46431100	-1.07884400

H	-1.04950700	-0.72324100	1.47974600
H	2.57198400	1.34400100	-1.19138100
H	1.13783900	-1.81416200	1.31983700
O	-1.30080300	1.87006600	0.81970500
H	-2.21894600	1.60790600	0.89997700
C	3.30918200	-1.01827800	-0.12002700
H	4.11136000	-0.32713200	0.14532600
H	3.38090300	-1.88911800	0.52809300
H	3.50887600	-1.34612100	-1.14297200
O	-3.13132500	-0.66931600	-0.69618200
O	-2.59231400	-1.67040600	-0.29870600

- **M20_pC**

C	-0.62681000	0.88862100	0.04359600
C	0.55209000	1.50134200	-0.61254900
C	-0.28459500	-0.32820000	0.82498900
C	1.74708700	0.87883800	-0.66461700
C	1.97726600	-0.36340000	-0.01852900
C	0.93898000	-0.91540600	0.74877200
H	-1.32043200	0.57968900	-0.76813900
H	0.39426100	2.46431100	-1.07884400
H	-1.04950700	-0.72324100	1.47974600
H	2.57198400	1.34400100	-1.19138100
H	1.13783900	-1.81416200	1.31983700
O	-1.30080300	1.87006600	0.81970500
H	-2.21894600	1.60790600	0.89997700
C	3.30918200	-1.01827800	-0.12002700
H	4.11136000	-0.32713200	0.14532600
H	3.38090300	-1.88911800	0.52809300
H	3.50887600	-1.34612100	-1.14297200
O	-3.13132500	-0.66931600	-0.69618200
O	-2.59231400	-1.67040600	-0.29870600

- **M22_pC**

C	0.61127100	-1.24780600	0.20143300
C	1.42551500	-0.01213800	0.36817400
C	0.62130600	1.23576900	0.20454600
C	-0.70588900	-1.23234800	0.03246700
C	-1.46653300	0.00564700	-0.03854100
C	-0.69497200	1.23710100	0.02645600
C	-2.79579800	0.01287700	-0.17612100
O	2.49364400	-0.08084500	-0.56840700
H	1.17090800	2.16962800	0.24360400
H	-1.25615900	-2.15948100	-0.07011300
H	-1.23584300	2.16924400	-0.08217800
H	-3.36014400	-0.90746800	-0.23103900
H	-3.34960400	0.93919600	-0.23788400
H	3.09915900	0.64105100	-0.39778300
H	1.16203100	-2.17882500	0.22685500

H	1.85110600	-0.02120000	1.38531500

- **M23_pC**

C	-1.03891400	0.58414200	-0.81270400
C	-1.29817200	-0.75049700	-0.16784600
C	-0.39974000	1.58900300	0.06067100
C	-0.01356300	-0.54061800	-0.91657000
C	1.15991100	-0.09529200	-0.12116000
C	0.80919900	1.19138100	0.47161400
H	-1.65778000	0.89125500	-1.64326200
H	-2.02404300	-1.37042300	-0.67515400
H	-0.88947400	2.50600300	0.35312800
H	0.14160000	-1.09611600	-1.82971600
C	2.30659800	-0.75106000	0.03479400
H	1.45494900	1.72087000	1.15713100
H	2.48730000	-1.69914900	-0.45124800
H	3.09866900	-0.35082200	0.65223200
O	-1.37371400	-0.95243500	1.18924800
H	-0.77342700	-0.34449400	1.63010600

- **M24_pC**

C	0.72377100	1.21626500	-0.01938100
C	1.37740000	0.05685500	-0.02307700
C	0.66728300	-1.20329000	0.11828700
C	-0.75381800	1.26776600	0.20013100
C	-1.46849000	-0.04604400	0.01191200
C	-0.66804200	-1.24986700	0.12992300
C	-2.77704200	-0.11705800	-0.23408600
O	2.73092500	0.02211500	-0.15439300
H	1.24847000	-2.11683700	0.18026200
H	-1.18559700	-2.19933300	0.17816100
H	-3.37950000	0.77114500	-0.36713000
H	-3.28121000	-1.07084200	-0.30823400
H	3.02374800	-0.88579900	-0.22775600
H	1.27392100	2.14162700	-0.11385400
H	-1.21202700	2.02724100	-0.43585600
H	-0.94157700	1.60811600	1.22729800

- **M25_pC**

C	-1.23851500	-0.00419300	-0.07682800
C	-0.39193300	-1.24205700	-0.12078200
C	-0.39174500	1.23868100	-0.12402400
C	0.92112700	-1.24370000	0.09338600
C	0.92267700	1.24179300	0.08465200
C	1.70220900	-0.00343600	0.38805500
C	-2.10152900	-0.00326400	1.18478400
O	-2.08350700	-0.07104100	-1.22874800
O	2.90934700	-0.07626300	-0.36338000

H	-0.93337400	-2.16416300	-0.33005800
H	-0.92564700	2.16952300	-0.32669700
H	1.49787300	-2.16624800	0.06401400
H	1.48690900	2.17483000	0.05295200
H	1.95675100	-0.00427100	1.46736600
H	-1.47600300	0.00585700	2.08482400
H	-2.74564000	0.88760800	1.21017000
H	-2.73877000	-0.89557100	1.20262100
H	-2.75171300	0.62172800	-1.16451200
H	3.48915500	0.64619100	-0.09910800

- **M26_pC**

C	1.05516700	-0.39795300	0.01619200
C	0.23594400	-0.29265100	1.25264300
C	0.24184800	-0.38151900	-1.22827800
C	2.02625200	-1.55331000	0.05941000
C	-1.04362500	0.03527900	1.23358300
C	-1.03992400	-0.06225600	-1.24346300
C	-1.79433400	0.33071500	-0.02263600
O	1.98550500	0.79125300	-0.02102800
O	1.35997200	1.91658700	-0.06451000
H	0.75230000	-0.47965600	2.18654000
H	0.76356800	-0.63318600	-2.14365300
H	1.47657800	-2.49187100	0.09097800
H	2.66163000	-1.54913300	-0.82497100
H	2.65841000	-1.48698300	0.94365300
H	-1.60513400	0.11272700	2.15783300
H	-1.60843000	-0.06190900	-2.16425400
O	-3.04870400	-0.32591900	-0.06336000
H	-3.61528400	0.04380800	0.61366700
H	-1.94579200	1.42100300	-0.07331900

- **M27_pC**

C	-1.07056800	-0.30838800	0.04251800
C	0.00377200	-1.15603000	-0.54362700
C	-0.60511900	0.13523000	1.40241800
C	-2.44790900	-0.90918300	0.00929400
C	1.31875200	-0.47542300	-0.60889700
C	0.53858400	0.80734000	1.36545600
C	1.07332200	0.92254700	-0.02494800
O	-1.17206400	0.89419700	-0.76841000
O	0.10083100	1.56026100	-0.86032100
H	-0.17472400	-2.14279900	-0.94754000
H	-1.20396700	-0.04352400	2.28544500
H	-2.46171000	-1.81575200	0.61298400
H	-3.17725300	-0.20842200	0.41100400
H	-2.72703900	-1.16181200	-1.01132200
H	1.65477000	-0.35177400	-1.64195300
H	1.01716600	1.27016000	2.21526400

O	2.35281200	-1.16801000	0.05917700
H	1.99800000	-1.48409900	0.89328600
H	1.95711400	1.54989100	-0.11401600

- **M28_pC**

C	-0.87596500	0.00000000	-0.09780100
C	-0.28094800	-1.23769700	-0.71526400
C	-0.28094900	1.23769800	-0.71526100
C	1.04010600	-1.24018200	-0.67258400
C	1.04010600	1.24018100	-0.67258700
C	1.57032200	0.00000000	-0.01158100
O	-0.39947400	-0.00000100	1.28849900
O	1.02631000	0.00000100	1.33771400
H	-0.90867800	-2.05345000	-1.04534400
H	-0.90867900	2.05345200	-1.04533800
H	1.68265800	-2.05699800	-0.96280400
H	1.68265800	2.05699600	-0.96280900
C	-2.37341500	0.00000000	0.01158800
H	2.64450600	0.00000000	0.14971500
H	-2.80710000	0.00000100	-0.98778000
H	-2.71779800	0.88515400	0.54279800
H	-2.71779800	-0.88515600	0.54279600

- **M30_pC**

C	-1.04803500	-0.37706000	0.00770400
C	-0.21202400	-0.37833700	-1.22528000
C	-0.23081600	-0.27629400	1.25150100
C	-1.98712200	-1.57259800	0.05008400
C	1.13015000	-0.21514800	-1.19830400
C	1.11099500	-0.10712700	1.23834800
C	1.81609200	-0.06550400	0.02099900
O	-1.99663200	0.72710900	-0.04162800
O	-1.29565200	1.94831300	-0.17269200
H	-0.74290400	-0.47743500	-2.16332000
H	-0.77525100	-0.32743500	2.18692400
H	-1.40919000	-2.49293700	0.09777600
H	-2.63049200	-1.51276700	0.92725200
H	-2.61067000	-1.58942200	-0.84286600
H	1.69536400	-0.19527900	-2.12388300
H	1.67587200	-0.01539900	2.15640300
O	3.15395000	0.10829200	0.08309000
H	3.51852800	0.12692900	-0.80324600
H	-1.09001600	2.16644600	0.74448700

- **M31_pC**

C	-0.68976600	-1.12389900	0.15313700
C	0.13213000	-1.24510200	-0.96051100

C	-0.21561200	-0.37066700	1.22219700
C	-2.03207000	-1.78158200	0.19774300
C	1.37821600	-0.64682100	-1.00958400
C	1.02851200	0.23803900	1.18910200
C	1.83542500	0.09593600	0.06948200
O	-2.52896100	1.62660900	-0.20390900
O	-1.33715000	2.03536000	-0.54395200
H	-0.21386400	-1.81522100	-1.81350900
H	-0.84117300	-0.24608500	2.09712300
H	-1.94347800	-2.84821900	0.40927200
H	-2.66086200	-1.33826100	0.96695300
H	-2.55205200	-1.67885200	-0.75373600
H	1.99932100	-0.75226000	-1.89120300
H	1.38908400	0.82359900	2.02287100
O	3.04387200	0.70380700	0.08185000
H	3.49744600	0.53910400	-0.74573900
H	-0.71751100	1.39456400	-0.13334400

- **TS13_pC**

C	-0.60804800	-1.20053300	-0.17676700
C	-1.29701100	0.00000400	0.00990900
C	0.72258900	-1.20951300	-0.52002800
C	-0.60804300	1.20053700	-0.17676800
C	0.72259500	1.20951000	-0.52003000
C	1.43513300	-0.00000300	-0.64046200
H	-1.13986000	-2.13596400	-0.05667100
H	1.24636700	-2.14334500	-0.66385100
H	-1.13985100	2.13597000	-0.05667200
H	2.41082900	-0.00000600	-1.09947400
O	2.41060500	-0.00000100	1.11736400
H	1.62966600	0.00000200	1.68309900
C	-2.73414300	0.00000100	0.41292100
H	-2.83357700	-0.00012700	1.50018800
H	-3.25158500	0.88356400	0.04293900
H	-3.25163700	-0.88344600	0.04273200
H	1.24637700	2.14333900	-0.66385400

- **TS14_pC**

C	-0.83108100	0.81744900	0.15870900
C	0.22026400	1.57158900	-0.51744600
C	-0.37939400	-0.27884800	1.00652600
C	1.47219800	1.05292000	-0.63452500
C	1.83315800	-0.15728900	0.02835200
C	0.91100900	-0.77751800	0.86395300
H	-1.31041700	0.20704900	-0.73215900
H	-0.06724700	2.50678300	-0.99183500
H	-1.03784000	-0.63655700	1.79118800
H	2.23097100	1.57806400	-1.21290100
H	1.22304300	-1.63615400	1.45573900
O	-1.87100100	1.61669100	0.66983400

H	-2.71099800	1.17252700	0.50148300
C	3.21523000	-0.69602000	-0.14204500
H	3.96864100	0.08202600	0.03380700
H	3.41407700	-1.53077700	0.53665900
H	3.36681800	-1.05499200	-1.16865000
O	-2.23665300	-1.04641700	-1.09763400
O	-1.85801500	-1.80548300	-0.17151000

- **TS15_pC**

C	-0.65451800	1.21584900	0.04710900
C	-1.39340000	-0.02389800	0.00581800
C	-0.64928900	-1.22141100	0.02824100
C	0.75139400	1.23134700	-0.07060300
C	1.52030800	0.01958000	-0.02533400
C	0.70613200	-1.18319100	-0.00867200
C	2.87340300	-0.06947200	-0.00471400
O	-2.72614100	0.03434700	-0.11951300
H	-1.17512600	-2.16872400	0.04104500
H	1.25326900	2.18725500	-0.07562400
H	1.24056600	-2.12635300	-0.00615700
H	3.49390700	0.81434100	-0.01424500
H	3.36664100	-1.02997600	0.00673500
H	-3.11957200	-0.79534500	0.16091500
H	-1.22789000	2.11403600	-0.15257300
H	-0.94685000	0.91716200	1.16493900

- **TS16_pC**

C	-0.69007200	-1.19810700	0.01851700
C	-1.41022200	0.01057900	-0.01655500
C	-0.67581000	1.20076200	0.01572100
C	0.77151800	-1.18982600	-0.07017200
C	1.52826900	0.02102300	-0.01630000
C	0.68769200	1.20760000	0.01614800
C	2.88653800	0.06220100	-0.01956400
O	-2.76302300	-0.05125900	-0.05864100
H	-1.21339600	-2.11803700	-0.21072900
H	-1.20835500	2.14566200	0.03237300
H	1.26571900	-2.15073500	-0.06350200
H	1.19542100	2.16354400	0.04655500
H	3.47252200	-0.84428400	-0.04586100
H	3.41375300	1.00413100	-0.02143400
H	-3.12703900	0.82660400	0.06339700
H	-0.28191600	-1.30219300	1.10155800

- **TS17_pC**

C	0.61997300	-1.22894200	-0.05912700
C	1.34879900	-0.08165800	0.05166000
C	0.74703300	1.19439300	-0.10591800
C	-0.78461600	-1.10495200	-0.29766400

C	-1.39074300	0.18665300	-0.12722300
C	-0.60864900	1.34365800	-0.21957600
C	-2.69627600	-0.04851900	0.36023800
O	2.69072400	-0.17971600	0.24171000
H	1.38305400	2.07135300	-0.05447800
H	-1.05798000	2.32785200	-0.21848100
H	-2.74737600	-0.46162400	1.35969500
H	-3.54945900	0.58028500	0.11252400
H	3.07896100	0.69513500	0.25762600
H	1.12759100	-2.18284700	-0.06905800
H	-2.01335800	-1.49267400	0.21740400
H	-1.16034200	-1.66355400	-1.15326300

- **TS18_pC**

C	-1.15736300	-0.39413700	0.08087700
C	-0.34309600	-0.80780600	-1.09262700
C	-0.40769400	-0.38899500	1.34561500
C	-2.55851100	-0.91536400	0.12207400
C	0.86928600	-0.24983100	-1.17742900
C	0.80290400	0.15865400	1.27947900
C	1.25333100	0.56842000	-0.04572500
O	-1.30831500	1.09210700	-0.38339600
O	-0.23601600	1.80907300	-0.14560300
H	-0.81719100	-1.34693100	-1.90010900
H	-0.88775200	-0.68419600	2.26740200
H	-2.54199200	-1.99479000	0.26678800
H	-3.11592300	-0.46202600	0.93905700
H	-3.07133100	-0.70191700	-0.81447600
H	1.49640100	-0.28033300	-2.05288200
H	1.43447200	0.35048100	2.13253900
O	3.05403200	-0.70557600	0.04637000
H	2.64116600	-1.57674700	0.09430000
H	2.03141800	1.30598300	-0.14516900

- **TS19_pC**

C	1.19491000	0.17862400	0.15155900
C	0.46260800	1.32237200	-0.48892700
C	0.45491400	-0.33111100	1.34021700
C	2.65529400	0.44330400	0.38809500
C	-0.86603200	1.23776600	-0.57276600
C	-0.87335600	-0.37725100	1.28025200
C	-1.51817000	0.08430000	0.04520100
O	1.17334000	-0.79893700	-0.98187400
O	0.09862800	-1.61368800	-0.97634500
H	1.03589200	2.10372900	-0.96779600
H	1.01336600	-0.68337100	2.19540700
H	2.77235800	1.26867100	1.08937200
H	3.14124800	-0.43970700	0.79817100
H	3.14692600	0.70945400	-0.54638300
H	-1.46413700	1.94704300	-1.13177400

H	-1.49848300	-0.80034000	2.05376900
O	-2.89547500	0.04176700	0.09083900
H	-3.25385600	0.35897000	-0.73839700
H	-0.96626400	-0.84560200	-0.67511700

- **TS20_pC**

C	-1.04998100	-0.56395400	0.00999900
C	-0.28369100	-0.60745500	-1.19485000
C	-0.30196900	-0.52382400	1.22949200
C	-2.40565900	-1.19922700	0.02284100
C	1.06206000	-0.37884400	-1.18798600
C	1.04239800	-0.29147600	1.23409500
C	1.73717600	-0.19438700	0.02210400
O	-1.72451800	1.15031900	-0.05186300
O	-0.72169300	2.08131600	-0.14766700
H	-0.80987800	-0.75224000	-2.12878300
H	-0.84407700	-0.61886400	2.16155900
H	-2.32088300	-2.28398700	0.07846600
H	-2.97781600	-0.85378700	0.88214100
H	-2.95427300	-0.94027700	-0.88068100
H	1.61917100	-0.34913300	-2.11710900
H	1.60014000	-0.20545800	2.15624100
O	3.06198100	0.04645700	0.08298000
H	3.42214400	0.09977500	-0.80391700
H	-0.46270500	2.23423100	0.77031000

- **pC**

C	1.43001900	-0.02030300	-0.00000300
C	0.71815000	-1.21161200	0.00000200
C	0.74689200	1.18350200	0.00000200
C	-0.66071800	-1.18370600	0.00001000
C	-1.36855000	0.01463300	0.00001100
C	-0.63877800	1.19212800	0.00001000
H	1.26280800	-2.14513100	0.00000300
H	1.29850600	2.11644500	0.00000300
H	-1.20777800	-2.11885900	0.00001600
H	-1.16073400	2.14082700	0.00001600
O	2.78400400	-0.09526200	-0.00000800
H	3.15189700	0.78898500	-0.00001200
C	-2.86518100	0.01589400	-0.00001300
H	-3.26316800	-0.49622800	-0.87674000
H	-3.26319700	-0.49678800	0.87637100
H	-3.26136200	1.02961800	0.00029900

Geometries of all the species reported in Fig.3 / CHANNEL A

- **BP (Benzoperoxyl radical)**

C	0.10014000	-0.23033800	0.43121400
C	-0.41153200	1.05956800	0.46746900
C	-1.72199100	1.30108000	0.09640500
C	-2.53067600	0.25426400	-0.31250000
C	-2.02752600	-1.03524100	-0.35048100
C	-0.71699600	-1.27481100	0.01994500
C	1.52190500	-0.48837500	0.78916900
H	0.22632500	1.87444100	0.78278100
H	-2.11394800	2.30789300	0.12768000
H	-3.55566200	0.44304000	-0.59949100
H	-2.65766400	-1.85429200	-0.66722600
H	-0.31989000	-2.28167700	-0.00946100
O	2.38970100	-0.40888300	-0.38151200
H	1.90635100	0.22867100	1.51264500
H	1.67464800	-1.50312900	1.15190100
O	2.55528600	0.81740500	-0.76175800

- **BO (Benzyloxy radical)**

C	0.00000000	0.53236300	0.00000000
C	1.33508300	0.15270900	0.00000000
C	-0.98501800	-0.44042500	0.00000000
C	1.68388800	-1.18524200	0.00000000
C	0.69565800	-2.15599600	0.00000000
C	-0.63692500	-1.78049600	0.00000000
C	-0.34694700	1.99548800	0.00000000
O	-1.64086300	2.33858800	0.00000000
H	2.10714400	0.91398400	0.00000000
H	-2.02242900	-0.13598600	0.00000000
H	2.72618100	-1.47225600	0.00000000
H	0.96459800	-3.20295900	0.00000000
H	-1.41027600	-2.53610200	0.00000000
H	0.14362500	2.50710600	0.85360500
H	0.14362500	2.50710600	-0.85360500

- **PhR (Phenyl radical)**

C	0.00000000	0.00000000	1.38631300
C	0.00000000	1.21576100	0.76520100
C	0.00000000	-1.21576100	0.76520100
C	0.00000000	1.20443000	-0.62660300
C	0.00000000	0.00000000	-1.31210500
C	0.00000000	-1.20443000	-0.62660300
H	0.00000000	2.14765200	1.31412000
H	0.00000000	-2.14765200	1.31412000
H	0.00000000	2.13889900	-1.17180700
H	0.00000000	0.00000000	-2.39304600
H	0.00000000	-2.13889900	-1.17180700

- **BPh (Biphenyl)**

C	-2.81505200	0.55330500	-0.48131600
C	-2.87159300	1.84939100	0.00168000
C	-1.71962700	2.44786200	0.48243900
C	-1.61731300	-0.13733000	-0.48347800
C	-0.45063300	0.45135200	-0.00196800
C	-0.52287000	1.75570900	0.48080300
C	0.82596300	-0.28589200	-0.00166300
C	1.72842300	-0.15137600	1.05046400
C	1.16258000	-1.13564200	-1.05246900
C	2.92583100	-0.84247000	1.05276400
C	3.24734900	-1.68315500	0.00096600
C	2.36025900	-1.82642100	-1.05206000
H	-3.71067700	0.07370600	-0.85126900
H	-3.80770700	2.38976300	0.00336500
H	-1.75111200	3.46280200	0.85384900
H	-1.58841900	-1.15812100	-0.84089900
H	0.37642900	2.24039300	0.83701600
H	1.47567400	0.48467600	1.88836800
H	0.48662700	-1.23609800	-1.89126100
H	3.60768000	-0.73025700	1.88432700
H	4.18352200	-2.22342500	0.00190300
H	2.60566700	-2.47377100	-1.88256400

<u>Geometries of all the species reported in Fig.3 / CHANNEL B</u>

- **TS_{BPBHP}**

C	-0.05071600	0.30135500	-0.11243000
C	0.33555100	-1.03811700	-0.09702700
C	1.67134600	-1.36862600	0.00699500
C	2.63221200	-0.37359500	0.10654800
C	2.25338500	0.96062700	0.10135100
C	0.92003500	1.29760300	-0.00503000
C	-1.45504000	0.67428100	-0.19633300
H	-0.42640200	-1.80110800	-0.16965400
H	1.96876000	-2.40773200	0.01312900
H	3.67709700	-0.63767400	0.18891600
H	3.00221900	1.73635300	0.17588400
H	0.61807400	2.33735900	-0.01573100
O	-2.35406200	-0.28325300	-0.61047300
H	-2.03783100	0.62542200	0.95077700
H	-1.69870800	1.68367100	-0.53391500
O	-3.01391900	-0.24892900	0.68124200

- **BHP (Benzylhydroperoxy radical)**

C	0.00898500	0.25565800	-0.06384800
C	0.47757400	-1.07723800	-0.04292500
C	1.82579300	-1.33867900	0.03245800
C	2.75163000	-0.30279200	0.08548200
C	2.30590700	1.01540700	0.06155500
C	0.96371700	1.29634900	-0.01441700
C	-1.34857500	0.57845700	-0.13985600
H	-0.23502700	-1.88822700	-0.08620300
H	2.16797900	-2.36441100	0.04966200
H	3.80863300	-0.51924400	0.14329600
H	3.02023700	1.82610800	0.10212200
H	0.62047300	2.32271800	-0.03614500
O	-2.25632000	-0.40349300	-0.31776700
H	-3.45993600	-0.37667700	1.11892800
H	-1.72937900	1.59083600	-0.14844200
O	-3.50657500	0.00923500	0.23602800

- **TS_{BHPBOH}**

C	0.00076200	0.28799100	-0.13275000
C	0.42280500	-1.05421800	-0.13414100
C	1.75592700	-1.36250700	0.01066000
C	2.70345600	-0.35522000	0.15118800
C	2.30177900	0.97462800	0.14737400
C	0.97120800	1.29617600	0.00681200
C	-1.35731800	0.65282300	-0.29228700
H	-0.31419700	-1.83563300	-0.25341400
H	2.06867600	-2.39747600	0.01230900
H	3.74885300	-0.60587400	0.26158700
H	3.03615500	1.76023700	0.25752200
H	0.65542000	2.33155500	0.00417600
O	-2.28149000	-0.24516200	-0.52292700
H	-3.04687100	-0.76472100	1.17370100
H	-1.66932300	1.69237600	-0.22191400
O	-3.37731500	-0.10714900	0.55104000

- **BOH (Benzaldehyde Hydroxy radical)**

C	0.16348800	0.07049800	-0.00000400
C	0.83667000	-1.14786800	-0.00000800
C	2.21488100	-1.16691600	-0.00000700
C	2.92465000	0.02695900	0.00000100
C	2.25983100	1.24141700	0.00000600
C	0.87766200	1.26199600	0.00000100
C	-1.30047900	0.11507600	-0.00000300
H	0.25865200	-2.06159900	-0.00001100
H	2.74505900	-2.10856800	-0.00001200
H	4.00566500	0.00746800	0.00000400
H	2.81852800	2.16619300	0.00001200
H	0.34031000	2.20226400	0.00000000

O	-2.01695600	-0.85617000	0.00002300
H	-3.79246600	-0.21285900	0.00001200
H	-1.73690100	1.13465100	-0.00002200
O	-4.54542700	0.41435300	-0.00001000

- **B (Benzaldehyde)**

C	0.52822300	0.20536300	0.00000000
C	0.03931600	-1.09675100	0.00000000
C	-1.32187800	-1.31767600	0.00000000
C	-2.19922900	-0.24128700	0.00000000
C	-1.71765700	1.05659900	0.00000000
C	-0.35249600	1.27796800	0.00000000
C	1.97916100	0.45923200	0.00000100
H	0.74636500	-1.91502800	0.00000100
H	-1.70825400	-2.32709800	0.00000000
H	-3.26587800	-0.41822800	0.00000000
H	-2.40507600	1.89043200	0.00000000
H	0.04202500	2.28691400	0.00000000
O	2.82631600	-0.38955200	0.00000000
H	2.24765200	1.53873700	-0.00000300

Geometries of all the species reported in Fig.3 / CHANNEL B’

- **BPw1 (Benzylperoxy radical water 1)**

C	1.27784900	-0.97762800	-0.64059700
C	0.51797200	0.07925000	-0.15534800
C	1.15717700	1.17306100	0.40865700
C	2.53865800	1.21577700	0.48062000
C	3.28846200	0.15828300	-0.00318100
C	2.65685600	-0.94101700	-0.56244800
C	-0.96505000	0.01779400	-0.22060400
O	-1.41144900	-0.94078200	0.79374100
O	-2.69738600	-1.06783200	0.80818200
H	0.56676500	1.99663000	0.78872100
H	3.02990200	2.07431300	0.91616900
H	4.36749000	0.19026800	0.05341700
H	3.24168900	-1.76717700	-0.94136900
H	0.77865400	-1.83242700	-1.07942200
H	-1.44678900	0.96976400	-0.00270100
H	-1.33566300	-0.35335400	-1.17633300
O	-3.84014800	1.09817900	-0.79044800
H	-3.69233900	0.30752600	-0.25851800
H	-4.74939700	1.34481500	-0.63436900

- **$TS1_{BPwBHPw}$**

C	1.29511600	1.19765800	-0.44339500
C	0.42305800	0.13654900	-0.20355400
C	0.93798300	-1.06867400	0.27187900
C	2.29548800	-1.20869300	0.48212700
C	3.15551000	-0.14899200	0.24000600
C	2.64995700	1.05610300	-0.22068800
C	-1.00825600	0.33368100	-0.41326000
O	-1.70122100	-0.82053500	-0.65817900
O	-3.05037100	-0.61081500	-0.61571200
H	0.26728400	-1.89299000	0.46900800
H	2.68695700	-2.14950700	0.84279000
H	4.21649700	-0.26158200	0.41217600
H	3.31596600	1.88549600	-0.41235700
H	0.90015500	2.13583700	-0.81198000
H	-1.65837800	0.77752400	0.61143500
H	-1.29293600	1.13760800	-1.09983300
O	-2.86283900	0.92765600	1.19454900
H	-3.15126800	0.17736100	0.32736000
H	-2.86196700	0.43400700	2.01744600

- **BHPw (Benzylhydroperoxy radical water)**

C	-1.33582800	-1.16306000	0.65731300
C	-0.34266700	-0.51481700	-0.11204000
C	-0.71733300	0.63452700	-0.84659300
C	-2.01009200	1.10299500	-0.79422400
C	-2.97221300	0.45681900	-0.02629300
C	-2.62093400	-0.67968000	0.69661300
C	0.96455200	-1.01765500	-0.12024200
O	1.88357500	-0.47273300	-0.93253000
O	3.15785200	-0.51271000	-0.28348900
H	0.02422400	1.13388500	-1.45437900
H	-2.28024200	1.98323100	-1.36152900
H	-3.98490900	0.83178200	0.00689100
H	-3.36479200	-1.18808900	1.29433100
H	-1.06720300	-2.04920000	1.21792700
H	1.26968200	1.12907300	1.40591700
H	1.28381000	-1.86640000	0.47192900
O	2.12401800	1.55517400	1.29478000
H	3.05735200	0.24394000	0.32943500
H	1.94562100	2.30915000	0.73219400

- **TS_{BHPwB}**

C	1.31308400	-1.21337100	-0.64748200
C	0.29601800	-0.62602200	0.12179200
C	0.59944800	0.52936800	0.86523500
C	1.86333400	1.07656700	0.81533000
C	2.85674600	0.48880900	0.04155600

C	2.57396300	-0.66033200	-0.68492400
C	-0.99908200	-1.21715600	0.14410800
O	-1.95522700	-0.72959800	0.86639200
O	-3.15881400	-0.37215200	-0.09767800
H	-0.17159700	0.96722200	1.48474300
H	2.08589800	1.96478900	1.39048700
H	3.84631800	0.92163400	0.00929000
H	3.34499900	-1.12097800	-1.28645000
H	1.08992500	-2.10671900	-1.21644100
H	-0.80197200	1.63035900	-0.86291000
H	-1.21636400	-2.08090200	-0.48350300
O	-1.70916100	1.90518700	-1.02283200
H	-2.77835600	0.45077100	-0.46146100
H	-1.83430200	2.67915700	-0.47449700

- **BHOw (Benzaldehyde-Hydroxy radical water)**

C	-1.06467500	1.11990500	0.09936000
C	-0.58798700	-0.18466600	0.06160300
C	-1.47170400	-1.25601500	-0.03611900
C	-2.82765500	-1.01554400	-0.09221900
C	-3.30191400	0.28961600	-0.05403300
C	-2.42518200	1.35713100	0.04018500
C	0.85720200	-0.40053000	0.12036900
O	1.41254600	-1.46875600	0.05415500
O	2.60238400	1.92900400	-0.10613200
H	-1.07373500	-2.26091300	-0.06617800
H	-3.52284900	-1.83956800	-0.16691900
H	-4.36656600	0.47284700	-0.09941600
H	-2.80295000	2.36898400	0.06641400
H	-0.35735400	1.93749100	0.16790300
H	4.57160200	-0.71281500	0.64264000
H	1.44609300	0.54502200	0.23273600
O	4.04509600	-0.48085600	-0.12007700
H	3.29945300	1.24010500	-0.16308800
H	3.25757700	-1.04565900	-0.07254500

- **TSBHOwBZO2w**

C	-1.05694300	1.11587600	0.01251700
C	-0.55791700	-0.18095100	0.01684200
C	-1.42080900	-1.27453600	0.00298400
C	-2.78235200	-1.06243000	-0.01401300
C	-3.28089800	0.23402400	-0.01829800
C	-2.42371400	1.32128100	-0.00556200
C	0.89218300	-0.37741000	0.03472100
O	1.47893900	-1.42059700	0.02283100
O	2.26739400	1.81890600	0.01473300
H	-1.00470800	-2.27236800	0.00568100
H	-3.46235900	-1.90226900	-0.02466000
H	-4.35002700	0.39483800	-0.03218600
H	-2.82149600	2.32569700	-0.01016900
H	-0.36408600	1.94717300	0.02045100

H	4.65230500	-0.53759400	0.66552800
H	3.10060400	1.31967100	-0.07342800
O	4.14720500	-0.35125400	-0.12396100
H	1.48623000	0.65415000	0.05978900
H	3.39792700	-0.96085900	-0.09496900

- **BZO2w (Benzoyl radical 2 waters)**

C	-0.81387600	1.01315200	0.06294500
C	-0.55723600	-0.35178100	0.01161700
C	-1.60089700	-1.27572600	-0.03704400
C	-2.90326700	-0.82421200	-0.03352300
C	-3.16111000	0.53992200	0.01561900
C	-2.12378600	1.45617200	0.06246800
C	0.83241600	-0.79530100	0.01895200
O	1.30489300	-1.87913700	-0.06056500
O	2.34790700	2.05157200	-0.03559800
H	-1.37127800	-2.33180900	-0.07608500
H	-3.72293300	-1.52796300	-0.06964200
H	-4.18474500	0.88873200	0.01756100
H	-2.33819900	2.51453700	0.10017300
H	0.03164100	1.69060000	0.09564200
H	4.34987200	-0.56844700	0.83328200
H	2.74885500	2.53076100	-0.75810000
O	3.89503700	-0.40837400	0.00801100
H	2.84243100	1.21933700	0.00754300
H	3.22820000	-1.10158400	-0.05136900

- **BZO (Benzoyl radical)**

C	0.33873900	-1.29393400	0.00000000
C	-0.55661600	-0.23421300	0.00000000
C	-0.09405200	1.07975500	0.00000000
C	1.26377500	1.32327100	0.00000000
C	2.15724500	0.26102400	0.00000000
C	1.69862500	-1.04569300	0.00000000
C	-2.00376300	-0.51017000	-0.00000100
O	-2.90217000	0.25068800	0.00000100
H	-0.81136200	1.88905000	-0.00000100
H	1.63290900	2.33928100	0.00000000
H	3.22071300	0.45617400	0.00000100
H	2.40107500	-1.86671200	0.00000100
H	-0.04969300	-2.30353400	0.00000000

- **BZOic (Benzoic Acid)**

C	0.00000000	0.21934800	0.00000000
C	-1.12545700	-0.59548200	0.00000000
C	1.26765900	-0.34948800	0.00000000
C	-0.97995600	-1.97009600	0.00000000
C	0.28529000	-2.53329300	0.00000000
C	1.40938600	-1.72278000	0.00000000
C	-0.09629800	1.69561700	0.00000000

O	0.84043300	2.44466700	0.00000000
O	-1.36622800	2.13761500	0.00000000
H	-2.10817400	-0.14824500	0.00000000
H	2.12771700	0.30498100	0.00000000
H	-1.85484900	-2.60455200	0.00000000
H	0.39626400	-3.60879300	0.00000000
H	2.39580000	-2.16424200	0.00000000
H	-1.31413900	3.09963300	0.00000000

Geometries of all the species reported in Fig.3 / CHANNEL B”

- **BPw2 (Benzylperoxy radical water 2)**

C	1.43308000	-1.17912500	-0.27523600
C	0.34360100	-0.33542700	-0.45543200
C	0.48632200	1.02617600	-0.22840000
C	1.70991000	1.53567700	0.17312600
C	2.79059100	0.69073700	0.35309800
C	2.65178500	-0.67000000	0.12975300
C	-0.97237300	-0.90380500	-0.85978300
O	-1.62363100	-1.57042400	0.27246500
O	-2.14625800	-0.73161300	1.10513600
H	-0.36409400	1.68187100	-0.36637500
H	1.81834700	2.59721200	0.34572600
H	3.74470400	1.09165100	0.66572400
H	3.49545500	-1.33142900	0.26786200
H	1.31863900	-2.24146000	-0.45231600
H	-1.66169700	-0.14675800	-1.23008700
H	-0.86710800	-1.71013200	-1.58336600
O	-2.72309400	1.78008200	-0.25150100
H	-2.62521100	1.07054400	0.39526000
H	-3.57267700	2.17873800	-0.07398700

Geometries of all the species reported in Fig.3 / CHANNEL B’’’

- **TS_{BPBHP}**

C	-0.05071600	0.30135500	-0.11243000
C	0.33555100	-1.03811700	-0.09702700
C	1.67134600	-1.36862600	0.00699500
C	2.63221200	-0.37359500	0.10654800
C	2.25338500	0.96062700	0.10135100
C	0.92003500	1.29760300	-0.00503000
C	-1.45504000	0.67428100	-0.19633300
H	-0.42640200	-1.80110800	-0.16965400
H	1.96876000	-2.40773200	0.01312900
H	3.67709700	-0.63767400	0.18891600
H	3.00221900	1.73635300	0.17588400
H	0.61807400	2.33735900	-0.01573100
O	-2.35406200	-0.28325300	-0.61047300
H	-2.03783100	0.62542200	0.95077700

H	-1.69870800	1.68367100	-0.53391500
O	-3.01391900	-0.24892900	0.68124200

Geometries of all the species reported in Fig.3 / CHANNEL C

- **TS_{BPhPB}**

C	-0.05071600	0.30135500	-0.11243000
C	0.33555100	-1.03811700	-0.09702700
C	1.67134600	-1.36862600	0.00699500
C	2.63221200	-0.37359500	0.10654800
C	2.25338500	0.96062700	0.10135100
C	0.92003500	1.29760300	-0.00503000
C	-1.45504000	0.67428100	-0.19633300
H	-0.42640200	-1.80110800	-0.16965400
H	1.96876000	-2.40773200	0.01312900
H	3.67709700	-0.63767400	0.18891600
H	3.00221900	1.73635300	0.17588400
H	0.61807400	2.33735900	-0.01573100
O	-2.35406200	-0.28325300	-0.61047300
H	-2.03783100	0.62542200	0.95077700
H	-1.69870800	1.68367100	-0.53391500
O	-3.01391900	-0.24892900	0.68124200

- **HPB (Hydroperoxybenzyl radical)**

C	0.05963200	-0.46381900	-0.02594900
C	-0.21797700	0.86945400	-0.13805800
C	-1.04859000	-1.29891700	0.10700200
C	-1.45444300	1.44764900	-0.13310600
C	-2.54093700	0.58947100	-0.00114800
C	-2.32976700	-0.77537400	0.11873300
C	1.45475400	-1.01974000	-0.03580000
O	2.42259300	-0.11863800	-0.50464000
O	2.63120000	0.85731600	0.50162000
H	-0.89862800	-2.36892600	0.19672800
H	-1.59524500	2.51577200	-0.22608300
H	-3.54585800	0.98873700	0.00660700
H	1.73146700	-1.37717900	0.96108200
H	1.51600200	-1.86595300	-0.72349400
H	2.00185300	1.54618300	0.24091500
H	-3.17596900	-1.44041000	0.21836000

Geometries of all the species reported in Fig.3 / CHANNEL F

- **$TS_{HPBHOBO}$**

C	0.00076200	0.28799100	-0.13275000
C	0.42280500	-1.05421800	-0.13414100
C	1.75592700	-1.36250700	0.01066000

C	2.70345600	-0.35522000	0.15118800
C	2.30177900	0.97462800	0.14737400
C	0.97120800	1.29617600	0.00681200
C	-1.35731800	0.65282300	-0.29228700
H	-0.31419700	-1.83563300	-0.25341400
H	2.06867600	-2.39747600	0.01230900
H	3.74885300	-0.60587400	0.26158700
H	3.03615500	1.76023700	0.25752200
H	0.65542000	2.33155500	0.00417600
O	-2.28149000	-0.24516200	-0.52292700
H	-3.04687100	-0.76472100	1.17370100
H	-1.66932300	1.69237600	-0.22191400
O	-3.37731500	-0.10714900	0.55104000

- **HOBO (Hydroxybenzyloxy radical)**

C	0.30756000	-0.51782200	0.00000100
C	-0.00327800	0.84746100	0.00000100
C	-0.73099200	-1.44431000	0.00000000
C	-1.33640400	1.24910200	-0.00000100
C	-2.34459300	0.31184300	-0.00000100
C	-2.04917000	-1.04742100	-0.00000100
C	1.71598700	-1.02013700	0.00000200
O	2.73286700	-0.15586800	-0.00000600
O	0.93731100	1.79935200	0.00000200
H	-0.48253200	-2.49998300	0.00000000
H	-1.54820300	2.30884900	-0.00000100
H	-3.37493600	0.63926300	-0.00000200
H	1.86080600	-1.72800300	0.84757400
H	1.86080300	-1.72802600	-0.84755000
H	1.81009100	1.36833900	0.00000200
H	-2.84211600	-1.78059700	-0.00000100

Geometries of all the species reported in Fig.3 / CHANNEL F1

- **HOBOw (Hydroxybenzyloxy radical)**

C	0.20729700	0.56840900	0.32026900
C	1.32918800	1.32953000	-0.00876400
C	0.27049700	-0.83403300	0.24909200
C	2.52902900	0.73167700	-0.30603600
C	2.60955300	-0.65933000	-0.29993900
C	1.50270800	-1.42802600	-0.02563900
C	-1.05727500	1.29089300	0.73432200
O	-1.59123700	1.52205600	-0.49723800
O	-0.75245100	-1.64865300	0.49801900
H	1.23731800	2.40767400	0.00536900
H	3.39906600	1.33036400	-0.53135800
H	3.54843700	-1.14448500	-0.52863900
H	1.54656900	-2.50758600	-0.03976500
H	-0.80194200	2.21070100	1.27197000
H	-1.67671100	0.64724300	1.37295400

H	-1.62343500	-1.29212800	0.22479400
H	-2.74313300	0.13672600	-0.78022300
O	-3.06978100	-0.76295900	-0.59962700
H	-3.92439400	-0.66679100	-0.18416200

- **$TS_{HOBOwoHMPOH}$**

C	0.28663000	-0.56493200	0.00000200
C	0.03167800	0.83195200	-0.00000200
C	-0.78423800	-1.45111100	0.00000300
C	-1.29469300	1.29564700	-0.00000400
C	-2.32389400	0.39465900	-0.00000300
C	-2.07712800	-0.98718600	0.00000000
C	1.69828300	-1.02931000	0.00000400
O	2.65674100	-0.09733000	0.00000300
O	1.03184100	1.66731000	-0.00000300
H	-0.58294900	-2.51609600	0.00000500
H	-1.46420000	2.36256900	-0.00000700
H	-3.34495600	0.74992900	-0.00000500
H	1.83046700	-1.73532100	0.85360600
H	1.83046900	-1.73532500	-0.85359400
H	1.90851700	1.05614200	-0.00000100
H	-2.90583100	-1.68005900	0.00000100

Geometries of all the species reported in Fig.3 / CHANNEL F1a

- **oHOMP (o-Hydroxymethylphenol salicyl alcohol)**

C	-0.28081300	-0.50711200	-0.15497000
C	0.73079400	-1.44979100	-0.04934900
C	0.05326800	0.84954400	-0.09348500
C	2.05415300	-1.07310700	0.09050700
C	2.37078900	0.27496500	0.13166400
C	1.37893200	1.23276600	0.04598700
C	-1.70288800	-0.91301900	-0.38113400
O	-2.61629400	-0.24098900	0.47930400
O	-0.88140700	1.81747600	-0.18366700
H	0.46796900	-2.50011800	-0.08932800
H	2.82929500	-1.82142900	0.16743600
H	3.40072900	0.58524900	0.24101500
H	1.60760700	2.28836300	0.08617600
H	-2.02345600	-0.64232600	-1.38957500
H	-1.80470600	-1.99772400	-0.29006600
H	-2.37194100	-0.43579700	1.38581600
H	-1.74930100	1.42642000	-0.01189200

Geometries of all the species reported in Fig.3 / CHANNEL F1b

- **oHOMCHDw (o-Hydroxymethyl cyclohexadienone water)**

C	0.28480000	0.63265000	0.27612000
C	1.45752800	1.32064000	0.11213000
C	0.29999200	-0.82197100	0.17823900
C	2.66049300	0.66347000	-0.15246700
C	2.70574400	-0.73448000	-0.25651100
C	1.56783900	-1.46257300	-0.09588000
C	-1.01335000	1.34307100	0.51475300
O	-1.76477100	1.42873200	-0.66317200
O	-0.72084600	-1.50650300	0.33655800
H	1.44796600	2.40074400	0.18636200
H	3.56741100	1.23882000	-0.27786200
H	3.64660800	-1.22489400	-0.46337900
H	1.55624300	-2.54136500	-0.16538900
H	-0.79855200	2.35653800	0.86317900
H	-1.56942500	0.83445400	1.30834500
H	-2.52799700	-1.24260200	0.03833700
H	-2.43043900	0.72298800	-0.65302500
O	-3.35216100	-0.83293800	-0.26760500
H	-3.96786900	-0.90385000	0.45888900

- **4OH2HOMCHDw (4-Hydroxy-2-Hydroxymethyl cyclohexadienone water)**

C	0.03791000	-0.51827600	0.35685200
C	-1.19666900	-1.01563800	0.38593100
C	0.23398900	0.93156700	0.15683900
C	-2.17707400	1.21618700	-0.11303700
C	-0.95764700	1.73681800	-0.14289600
C	1.25333800	-1.38805100	0.49133200
O	1.93589900	-1.51301800	-0.72176800
O	1.32739100	1.46322800	0.23069100
H	-1.35849700	-2.07964100	0.51718100
H	-3.04891500	1.81876200	-0.34310000
H	-0.77314900	2.77356900	-0.38901800
H	0.93179100	-2.37988700	0.82152500
H	1.90362900	-0.98215500	1.27373200
H	3.10376400	0.97406000	-0.01366200
H	2.72031700	-0.94524500	-0.69405000
O	3.87747800	0.45668000	-0.28451400
H	4.46666600	0.45082500	0.46694100
C	-2.43204000	-0.19742700	0.28213400
H	-2.86257500	-0.16389200	1.29987300
O	-3.32474300	-0.85963300	-0.58680400
H	-4.18206900	-0.43553800	-0.54319900

Geometries of all the species reported in Fig.3 / CHANNEL F2

- **$TS_{HOBOoHOMCHD}$**

C	0.28663000	-0.56493200	0.00000200
C	0.03167800	0.83195200	-0.00000200
C	-0.78423800	-1.45111100	0.00000300
C	-1.29469300	1.29564700	-0.00000400
C	-2.32389400	0.39465900	-0.00000300
C	-2.07712800	-0.98718600	0.00000000
C	1.69828300	-1.02931000	0.00000400
O	2.65674100	-0.09733000	0.00000300
O	1.03184100	1.66731000	-0.00000300
H	-0.58294900	-2.51609600	0.00000500
H	-1.46420000	2.36256900	-0.00000700
H	-3.34495600	0.74992900	-0.00000500
H	1.83046700	-1.73532100	0.85360600
H	1.83046900	-1.73532500	-0.85359400
H	1.90851700	1.05614200	-0.00000100
H	-2.90583100	-1.68005900	0.00000100

- **oHOMCHD (o-Hydroxymethyl cyclodienhexenone radical)**

C	-0.34281500	-0.50430300	0.15172100
C	0.03470400	0.90309900	0.10632900
C	0.62901300	-1.46260600	0.05205000
C	1.43754600	1.21630700	-0.04379100
C	2.36987300	0.22846500	-0.13829900
C	1.97455500	-1.11618700	-0.09113200
C	-1.78994300	-0.83230600	0.34676300
O	-2.62935400	-0.19739600	-0.57612800
O	-0.81784800	1.79778400	0.19706900
H	0.34997400	-2.50850100	0.08253100
H	1.69854300	2.26487100	-0.07863400
H	3.41684600	0.47198800	-0.25286300
H	-1.93859400	-1.90729400	0.23886000
H	-2.06647100	-0.57219300	1.37850500
H	-2.48068900	0.74818300	-0.46672700
H	2.72040600	-1.89498200	-0.17104600

Geometries of all the species reported in Fig.3 / CHANNEL F3

- **$TS_{HOBOHOP}$**

C	-0.13351200	-0.50890100	0.01026600
C	0.09864900	0.84787400	0.02876500
C	0.80136000	-1.49775800	-0.03736800
C	1.44501700	1.21502400	0.01569000
C	2.43583900	0.25186500	-0.02296700
C	2.13463100	-1.10140200	-0.05197500
C	-2.30168000	-0.98470900	0.26115600

O	-2.84221200	-0.07751200	-0.34470900
O	-0.84352000	1.79504200	0.07008300
H	0.52540800	-2.54412600	-0.05947800
H	1.68318100	2.27018000	0.03264800
H	3.46993100	0.56657700	-0.03546200
H	-2.16678900	-1.98266000	-0.19031300
H	-2.10807400	-0.92809600	1.34592900
H	2.91988200	-1.84263900	-0.08628200
H	-1.71951500	1.38856900	-0.03143200

- **HOP.CH2O (Hydroxyphenyl radical-formaldehyde)**

C	0.03893200	0.56677100	-0.00001600
C	0.28509300	-0.79436500	-0.00000300
C	0.99507700	1.55237800	-0.00002500
C	1.63276300	-1.17716900	0.00000200
C	2.63523800	-0.21284400	-0.00000700
C	2.33525700	1.14971400	-0.00002000
C	-3.15586300	0.97002700	0.00005000
O	-3.09055700	-0.23534400	-0.00000400
O	-0.66923600	-1.74387300	0.00000500
H	0.72911000	2.60823500	-0.00003600
H	1.86560500	-2.24016600	0.00001200
H	3.67461000	-0.53300900	-0.00000200
H	-4.13183800	1.49451600	0.00010500
H	-2.24069200	1.60162300	0.00004400
H	-1.54662000	-1.31698800	-0.00000500
H	3.12918100	1.89246100	-0.00002700

- **HOP (Hydroxyphenyl radical)**

C	-0.96885100	-0.04590000	-0.00000100
C	-0.20488900	-1.18432900	0.00000000
C	-0.28079600	1.16320800	0.00000000
C	1.15663200	-1.22873200	0.00000000
C	1.83089500	-0.01071100	0.00000000
C	1.10295800	1.16900500	0.00000000
H	-0.84973000	2.08352300	0.00000100
H	1.69797500	-2.16535100	0.00000100
H	2.91141200	0.01007800	0.00000000
H	1.62334300	2.11648100	0.00000100
O	-2.32032600	-0.03495500	0.00000000
H	-2.63609200	-0.94032800	0.00000400

<u>Geometries of all the species reported in Fig.3 / CHANNEL F3a</u>

- **C (Catechol)**

C	0.50176800	-0.72612500	0.00000000
C	0.52951200	0.66755500	0.00000000
C	-0.71489400	-1.37927100	0.00000000

C	-0.64571600	1.38910700	0.00000000
C	-1.86508800	0.72808900	0.00000000
C	-1.89582200	-0.65380600	0.00000000
H	-0.71664300	-2.46011200	0.00000000
H	-0.60495400	2.47203800	0.00000000
H	-2.78334100	1.29684200	0.00000000
H	-2.84183000	-1.17568500	0.00000000
O	1.65126600	-1.43422500	0.00000000
H	2.38661900	-0.81522700	0.00000000
O	1.77343900	1.22756900	0.00000000
H	1.70395300	2.18210500	-0.00000100

Geometries of all the species reported in Fig.3 / CHANNEL F3b

- **HOPw (Hydroxyphenyl radical water)**

C	-0.18850200	-0.65885500	-0.01048900
C	-0.18427600	0.71747900	-0.02240700
C	1.06721000	-1.26545700	0.01172300
C	0.91852700	1.51891000	-0.01233400
C	2.16010500	0.89068200	0.00985500
C	2.21535200	-0.49484700	0.02159300
H	1.11348900	-2.34637600	0.02048700
H	0.84244200	2.59820800	-0.02205500
H	3.06631800	1.47923600	0.01739500
H	3.17693700	-0.98881800	0.03870900
O	-1.28987100	-1.42013500	-0.01975700
H	-2.07291500	-0.84386100	-0.03738200
H	-2.51866800	1.27652200	-0.24065100
O	-3.21111700	0.63451300	-0.05327200
H	-3.53018800	0.86258800	0.82007300

- **$TS_{HOPwPhOH}$**

C	0.23184500	0.75701900	-0.02971500
C	0.37390700	-0.61972900	-0.10038700
C	-1.06291600	1.26625600	0.02085000
C	-0.67834100	-1.49895400	-0.03286400
C	-1.96264200	-0.97659000	0.02072100
C	-2.13825200	0.40011000	0.04561000
H	-1.19633900	2.33848600	0.05953400
H	-0.51635100	-2.56745300	-0.05581500
H	-2.81534000	-1.63977100	0.03595800
H	-3.13866300	0.80725000	0.09585900
O	1.27843800	1.58996500	-0.02291200
H	2.08653600	1.05206100	-0.02562200
H	1.56176900	-0.96731600	-0.25259600
O	2.79626900	-0.82439700	-0.01524600
H	2.83911800	-1.11647400	0.90266200

- **PhOH (Phenol OH radical)**

C	0.39431000	0.66901200	-0.27848700
C	0.30059000	-0.68541800	-0.62603400
C	-0.72339400	1.33297700	0.21623600
C	-0.88863500	-1.37338700	-0.40302100
C	-1.98600900	-0.71986400	0.11223600
C	-1.89967400	0.64049200	0.40416800
H	-0.63557000	2.38074200	0.46479000
H	-0.94949200	-2.41994300	-0.66576400
H	-2.91311900	-1.25039100	0.27281900
H	-2.76266700	1.15848400	0.79930100
O	1.54037000	1.34982500	-0.41141300
H	2.25726100	0.71030900	-0.28327200
H	1.11690600	-1.15706200	-1.15185200
O	2.31836300	-1.05468100	0.71935600
H	1.83368800	-0.96617000	1.54985000

- **Ph (Phenol)**

C	0.93390600	-0.02694500	0.00000000
C	0.26401700	1.18742100	0.00000000
C	0.21721000	-1.21483600	0.00000000
C	-1.11987500	1.21028800	0.00000000
C	-1.84173300	0.03024200	0.00000000
C	-1.16351500	-1.17872400	0.00000000
H	0.75875700	-2.15000800	0.00000100
H	-1.63469700	2.16102800	0.00000000
H	-2.92172200	0.05077900	0.00000000
H	-1.71686000	-2.10765200	0.00000000
O	2.28569000	-0.11032400	-0.00000100
H	2.66046900	0.77120400	0.00000900
H	0.82847400	2.11257300	-0.00000100

Geometries of all the species reported in Fig.3 / CHANNEL G

- **$TS_{HPBBOEOH}$**

C	-0.11978700	-0.66562400	0.00376100
C	-0.17050300	0.69851200	-0.00270800
C	-1.31281500	-1.36203300	0.00847200
C	-1.27652900	1.48847700	-0.00869700
C	-2.47577600	0.77012900	-0.00476000
C	-2.48830600	-0.61733300	0.00389600
C	1.33577400	-0.93999400	0.00558900
O	1.74977300	0.43685600	0.00295200
O	3.43150000	0.23115200	-0.06434900
H	-1.34457100	-2.44300200	0.01453900
H	-1.25679500	2.56807800	-0.01607200
H	-3.41263500	1.31108500	-0.00895900
H	1.71215900	-1.45069500	0.89347200

H	1.71046700	-1.44905500	-0.88424200
H	3.62801500	1.01796700	0.45256700
H	-3.43916900	-1.13125200	0.00655400

- **BOEOH (Benzo[2,3-b]oxoetane OH radical)**

C	0.22593000	0.86270900	0.14260700
C	0.01743300	-0.48319000	0.34743700
C	1.44006600	1.35601500	-0.25151700
C	0.96404300	-1.45804100	0.18717800
C	2.20543400	-0.95676100	-0.21546800
C	2.44038900	0.39526000	-0.42777000
C	-1.21286500	1.05915700	0.52316700
O	-1.30972000	-0.40223500	0.71742600
O	-3.57846900	-0.31885700	-0.90510200
H	1.63750000	2.40450500	-0.42167300
H	0.79243600	-2.51080500	0.34942300
H	3.01872200	-1.65254300	-0.36856000
H	-1.42261800	1.57605700	1.45687100
H	-1.89996100	1.37902600	-0.26062500
H	-2.92952000	-0.78790400	-0.34923100
H	3.42637400	0.70951200	-0.73860300

- **BOE (Benzo[2,3-b]oxoetane)**

C	-0.81513900	-0.31156400	0.00000000
C	-0.31089700	-1.58319400	0.00000000
C	0.00000000	0.80103000	0.00000000
C	1.08556100	-1.66776400	0.00000000
C	1.88730300	-0.53420600	0.00000000
C	1.36923600	0.76447400	0.00000000
C	-1.96703300	0.65441600	0.00000000
O	-0.99054600	1.75129500	0.00000000
H	-0.92266200	-2.47409300	0.00000000
H	1.55716900	-2.64002100	0.00000000
H	2.96112500	-0.66218100	0.00000000
H	2.00331000	1.63755900	0.00000000
H	-2.58437800	0.69461500	0.89548400
H	-2.58437800	0.69461500	-0.89548400

Geometries of all the species reported in Fig.3 / CHANNEL D

- $TS_{BPDOESB}$

C	0.35473400	0.14692600	-0.27605800
C	-0.26630500	-1.14921300	-0.30624700
C	-0.50417200	1.28563700	-0.13873500
C	-1.61576300	-1.29100800	-0.07102100
C	-2.41781400	-0.15955900	0.13387200
C	-1.85074700	1.12638900	0.09133900
C	1.75554200	0.29360700	-0.84558600

O	2.45698700	-0.29371000	0.23256900
O	1.41886800	0.00385900	1.18550400
H	0.37715100	-2.01403400	-0.45642500
H	-0.05375300	2.27593100	-0.16780400
H	-2.06181400	-2.28208000	-0.04859800
H	-3.48297500	-0.27517200	0.31905200
H	-2.48400000	1.99873500	0.23297500
H	2.01930300	1.35577100	-0.97383800
H	1.94638500	-0.25701200	-1.77533100

- **DOESB (1,2-Dioxetanspirobenzyl radical)**

C	0.43054400	-0.08285400	0.03190900
C	-0.29555100	1.20567100	0.02199300
C	-0.44662400	-1.26570800	0.00566400
C	-1.64837300	1.27060200	0.01702000
C	-2.42721400	0.09845100	0.01650200
C	-1.79859700	-1.15931100	0.00289600
C	1.64306500	-0.11956700	0.96877400
O	2.49814300	0.19030700	-0.12440300
O	1.42968500	-0.11679800	-1.06202600
H	0.31796200	2.09774800	0.00105600
H	0.04469400	-2.22971300	-0.02379300
H	-2.14055500	2.23343900	0.00247200
H	-3.50556000	0.16441100	0.00789100
H	-2.40589300	-2.05387900	-0.01685000
H	1.84071600	-1.11561600	1.37524600
H	1.68250800	0.63182300	1.75686100

- **$TS_{DOESBpOHCHD}$**

C	0.33137700	-0.11527000	-0.26007700
C	-0.29268300	1.16139200	-0.29090300
C	-0.49007800	-1.25917100	-0.17293700
C	-1.62739600	1.27594200	-0.00518700
C	-2.40581700	0.13717300	0.20671600
C	-1.83240300	-1.12371700	0.09799600
C	1.67630200	-0.04514800	1.12485700
O	2.46485600	0.25684000	0.04527100
O	1.55812800	-0.27309900	-0.97506300
H	0.32583400	2.02535300	-0.49320900
H	-0.02548500	-2.22926500	-0.28372200
H	-2.08758100	2.25381100	0.02488300
H	-3.46128100	0.23561800	0.41586100
H	-2.44702600	-2.00581600	0.21122100
H	1.73061600	-1.07191300	1.48413900
H	1.62522700	0.73507800	1.87637100

- **PhO (Phenoxy radical)**

C	0.00000000	0.00000000	1.04531600
C	0.00000000	1.23253200	0.28656500

C	0.00000000	-1.23253200	0.28656500
C	0.00000000	1.21706200	-1.07762600
C	0.00000000	0.00000000	-1.76997500
C	0.00000000	-1.21706200	-1.07762600
H	0.00000000	2.15317000	0.85346100
H	0.00000000	-2.15317000	0.85346100
H	0.00000000	2.14407300	-1.63424500
H	0.00000000	0.00000000	-2.85119600
H	0.00000000	-2.14407300	-1.63424500
O	0.00000000	0.00000000	2.28168100

- **pOHCHD (p-Hydroxy cyclohexadienone)**

C	-1.50196800	0.00000000	-0.05084400
C	-0.72669400	-1.25127700	0.02172900
C	-0.72669400	1.25127700	0.02172900
C	0.58785300	-1.24895500	0.20799100
C	1.38474800	0.00000000	0.37854200
C	0.58785300	1.24895500	0.20799100
O	-2.70510700	0.00000000	-0.18321000
O	2.53247800	0.00000000	-0.44937500
H	-1.29410300	-2.16489400	-0.09394700
H	-1.29410300	2.16489400	-0.09394700
H	1.15666500	-2.17022700	0.24415400
H	1.78647100	0.00000000	1.40019100
H	1.15666500	2.17022700	0.24415400
H	2.23885100	0.00000000	-1.36274800

- **pPoxCHD (p-Peroxo cyclohexadienone radical)**

C	1.87968100	-0.11083000	-0.06047900
C	1.24514200	1.22050600	-0.00681300
C	0.97175400	-1.26927000	0.02206000
C	-0.05952800	1.36798900	0.18054500
C	-0.95340100	0.20924600	0.41809500
C	-0.33225200	-1.12326300	0.21587000
O	3.07434900	-0.24121300	-0.19143400
O	-2.10808900	0.36628900	-0.47410000
H	1.90764200	2.06488400	-0.13790500
H	1.43221000	-2.24063600	-0.09482100
H	-0.52504000	2.34510200	0.20130300
H	-1.37366300	0.26493600	1.42911700
H	-1.00748300	-1.96803800	0.26299800
O	-3.08401500	-0.40414000	-0.11901100

- **$TS_{pPoxpHPox}$**

C	0.06646900	-1.91872600	0.00000000
C	0.09977900	-1.13508300	1.23651700
C	0.09977900	-1.13508300	-1.23651700
C	0.09977900	0.21026100	1.23245100
C	-0.09096300	0.91369800	0.00000000

C	0.09977900	0.21026100	-1.23245100
O	0.02589400	-3.13587600	0.00000000
O	0.44127100	2.26424800	0.00000000
H	0.10551400	-1.69724100	2.15984700
H	0.10551400	-1.69724100	-2.15984700
H	0.17079600	0.78391100	2.14779300
H	-1.24674300	1.99097100	0.00000000
H	0.17079600	0.78391100	-2.14779300
O	-0.66136500	2.99209200	0.00000000

- **pHPoxCHD (p-Hydroperoxo cyclohexadienone radical)**

C	-1.91658200	-0.13845600	0.00479300
C	-0.98059400	-1.23996000	0.00000900
C	-1.33730000	1.18919100	-0.00033500
C	0.37049500	-1.04599100	-0.00447100
C	0.86407300	0.26090200	-0.00361700
C	0.00650600	1.37623000	-0.00726000
O	-3.13874400	-0.31821500	0.01198700
O	2.17561300	0.58811700	-0.00279500
H	-1.40298200	-2.23511400	0.00017500
H	-2.02475300	2.02324400	-0.00053900
H	1.05892500	-1.87527100	-0.01120300
H	3.60663600	-0.38242700	0.64697700
H	0.44691800	2.36375600	-0.01495000
O	2.99758900	-0.55811300	-0.07858800

- **$TS_{pHPoxpBQ}$**

C	1.91345100	-0.12912700	0.06110300
C	0.99247500	-1.25564400	-0.10817500
C	1.31586300	1.20498500	0.10374700
C	-0.33095400	-1.06756300	-0.24473300
C	-0.87285900	0.25761300	-0.22451600
C	-0.01546000	1.37901500	-0.00430600
O	3.11725500	-0.29707300	0.16653400
O	-2.11917500	0.48484400	-0.44009600
H	1.44064000	-2.23928400	-0.12875700
H	1.99692800	2.03258600	0.24462200
H	-1.02452700	-1.88698300	-0.36702400
H	-3.76548200	-0.55474400	0.00940100
H	-0.47963800	2.35435800	0.03996100
O	-3.02095600	-0.44297200	0.61144800

- **pBQ (1,4-Benzoquinone)**

C	1.43537000	0.00000000	0.00000000
C	0.66463500	-1.26090200	0.00000000
C	0.66463500	1.26090200	0.00000000
C	-0.66463500	-1.26090200	0.00000000
C	-1.43537000	0.00000000	0.00000000

C	-0.66463500	1.26090200	0.00000000
O	2.64381300	0.00000000	0.00000000
O	-2.64381300	0.00000000	0.00000000
H	1.25262900	-2.16892600	0.00000000
H	1.25262900	2.16892600	0.00000000
H	-1.25262900	-2.16892600	0.00000000
H	-1.25262900	2.16892600	0.00000000

Absolute E+ZPE energies of the species studied in this work.

All energies are referred to Toluene (T) plus two hydroxyl radicals and one molecular oxygen species ($T+2OH+O_2$). When necessary, H_2O or HOO was added at the r.h.s. of the equation to keep the balance, even if not explicitly listed. G4 E+ZPE energies were used to build Figs. 1 to 3 in the main text of the manuscript.

Table S2. Species included in Fig. 1 in the main text. Δ (E+ZPE) in kcal mol^{-1}.

Chemical Species		Theoretical Method		
**Type**	**Species**	**CBS-QB3**	**G4**	**M06/cc-pVQZ**
**Reactants (reference)**	T+2OH + O_2	0.0	0.0	0.0
**Intermediates**	M01_TR	-1.5	-1.7	-4.6
	M02_TR	-28.8	-29.8	-31.4
	TR	-28.5	-28.2	-29.0
	M03_oC	-18.0	-17.6	-19.3
	M04_oC	-18.9	-18.2	-19.9
	M05_oC	-54.8	-55.3	-54.2
	M07_oC	-29.7	-31.1	-27.6
	M08_oC	-46.4	-44.4	-44.5
	M09_oC	-47.4	-45.5	-45.7
	M10_oC	-17.7	-18.4	-13.6
	M11_oC	18.1	18.4	25.1
	M12_oC	-79.2	-80.1	-77.7
	M13_oC	-100.5	-99.2	-101.1
	M14_oC	-75.7	-74.0	-76.0
	M15_oC	-23.4	-23.2	-24.5
**Transition States**	TS01_TR	1.3	-0.5	-1.9
	TS02_oC	-1.1	-0.4	-3.2
	TS03_oC	-13.0	-10.1	-18.7
	TS04_oC	-6.3	-17,2	-17.0
	TS05_oC	-44.1	-42.2	-42.4
	TS06_oC	-38.9	-35.3	-36.5
	TS07_oC	-4.8	-3.4	0.3
	TS08_oC	-28.1	-27.3	-31.4
	TS09_oC	-49.1	-47.6	-52.1
	TS10_oC	-29.0	-28.9	-29.5
	TS11_oC	-10.4	-9.8	-9.4
	TS12_oC	-20.5	-19.0	-21.6
**Products**	BA (M28_TR)	-110.0	-107.6	-107.2
	M16_oC	-91.6	-89.9	-89.9
	oC (M06_oC) + OOH	-48.6	-47.3	-48.1
	oC (M06_oC) + H_2O	-118.5	-116.1	-116.8

Table S3. Species included in Fig. 2 in the main text (some of the species are already included in Table S2). Δ(E+ZPE) in kcal mol^{-1}.

**Chemical Species**		**Theoretical Method**		
**Type**	**Species**	**CBS-QB3**	**G4**	**M06/cc-pVQZ**
**Intermediates**	M17_pC	-1.0	-2.1	-3.8
	M18_pC	-16.9	-16.5	-18.0
	M19_pC	-18.9	-30.0	-18.7
	M20_pC	-53.5	-54.1	-53.3
	M22_pC	-83.0	-82.2	-79.6
	M23_pC	-59.0	-57.7	-61.8
	M24_pC	-83.9	-82.7	-82.4
	M25_pC	-86.2	-85.0	-83.2
	M26_pC	-28.6	-31.0	-24.9
	M27_pC	-20.6	-21.5	-15.3
	M28_pC	16.5	16.8	24.2
	M30_pC	-46.7	-45.1	-44.0
	M31_pC	-53.8	-54.3	-52.3
**Transition States**	TS13_pC	-0.2	0.9	-1.9
	TS14_pC	-11.8	-8.9	-17.5
	TS15_pC	-27.2	-26.5	-30.2
	TS16_pC	-28.6	-26.9	-29.6
	TS17_pC	-24.1	-21.6	-24.1
	TS18_pC	---	36.7	39.7
	TS19_pC	-8.8	-6.9	-1.9
	TS20_pC	-40.0	-36.5	-36.2
**Products**	pC (M21_pC) + HOO	-47.6	-46.3	-47.5
	pC (M21_pC) + H_2O	-117.4	-115.0	-116.2

Table S4. Species included in Fig. 3 in the main text (some of the species are already included in Tables S2 or S3). Δ(E+ZPE) in kcal mol^{-1}.

**Chemical Species**		**Theoretical Method**		
**Reaction Channel**	**Species**	**CBS-QB3**	**G4**	**M06/cc-pVQZ**
**Channel A**	BP	-50.7	-50.9	-47.0
	BO	10.6	9.2	9.4
	PhR + CH_2O	35.9	34.0	31.3
**Channel B**	TS_{BPBHP}	16.4	17.9	21.4
	BHP	---	---	-19.0
	TS_{BHPBOH}	---	---	-19.4
	BOH	-55.9	-57.3	-54.4
	B	-51.4	-50.9	-49.5
**Channel B'**	BPw1	-33.4	-39.0	-21.5
	$TS1_{BPwBHPw}$	9.2	10.8	9.9
	BHPw	-28.6	-27.1	-26.0
	TS_{BHPwB}	-28.7	-25.8	-25.4
	BHOw	---	---	-61.6
	$TS_{BHOwBZO2w}$	-62.0	-62.0	-62.9
	BZO2w	-86.5	-86.4	-84.4
	BZO	-79.7	-80.1	-75.6
	BZOic	-188.9	-186.0	-185.4
**Channel B''**	BPw2	-26.1	-26.8	-22.5
**Channel C**	TS_{BPHPB}	10.2	10.1	12.7
	HPB	??	??	-19.0
**Channel D**	$TS_{BPDOESB}$	8.4	11.9	16.3
	DOESB	4.0	5.5	10.0
	PhO.CH_2O	16.5	20.4	21.3
	pOHCHD	-127.1	-125.3	-125.5
	pPoxCHD	-65.6	-66.6	-63.1
	$TS_{pPoxpHPox}$	-15.5	-15.5	-16.9
	pHPoxCHD	-89.4	-86.2	-88.0
	$TS_{pHPoxpBQ}$	-83.9	-78.2	-80.7
	pBQ	-97.2	-97.0	-96.3
**Channel F**	$TS_{HPBHOBO}$	29.2	31.2	35.3
	HOBO	-64.4	-64.9	-66.7
**Channel F1**	HOBOw	-68.5	-68.7	-70.8
**Channel F1a**	oHOMP	-52.2	-51.1	-50.7
**Channel F1b**	oHOMCHDw	-90.0	-88.2	-89.4
	4OH2HOMCHDw	-149.9	-147.3	-147.0
**Channel F2**	$TS_{HOBOoHOMCHD}$	-64.7	-58.8	-66.5
	oHOMCHD	-84.3	-82.0	-82.8
**Channel F3**	$TS_{HOBOHOP}$	-40.1	-41.3	-44.7
	HOP.CH2O	-42.5	-44.2	-44.7
	HOP	-36.7	-37.8	-40.9
**Channel F3a**	C	-152.1	-148.6	-151.2
**Channel F3b**	HOPw	-41.7	-43.0	-47.7
	$TS_{HOPwPhOH}$	-31.5	-30.6	-34.1

	PhOH	-34.0	-34.5	-39.9
	Ph	-33.2	-32.6	-34.2
**Channel G**	$TS_{HPBBOEOH}$	10.2	10.1	12.7
	BOEOH	-22.8	-23.7	-19.7
	BOE	-18.8	-17.9	-17.0

Table S5. Some species corresponding to the *meta* addition are included in Scheme 2 in the main text. Δ(E+ZPE) in kcal mol^{-1}.

Chemical Species		Theoretical Method		
**Type**	**Species**	**CBS-QB3**	**G4**	**M06/cc-pVQZ**
**Intermediates**	M32_mC	-1.0	-2.1	-3.8
	M33_mC	-16.6	-16.3	-17.4
	M34_mC	-23.5	-21.2	-24.5
	M35_mC	-54.1	-54.8	-54.0
	M37_mC	-28.6	-30.4	-25.8
	M40_mC	-46.1	-44.1	-44.6
	M41_mC	-53.9	---	---
	M44_mC	-85.7	-84.5	-83.6
	M46_mC	-69.0	-68.0	-69.8
**Transition States**	TS21_mC	0.2	1.2	-1.2
	TS22_mC	-16,7	---	-16.7
	TS23_mC	-17.4	-14.2	-17.5
	TS24_mC	-7.0	-5.1	---
	TS26_mC	-40.2	---	---
**Products**	mC(M36_mC) + OOH	-48.0	-46.8	-48.2
	mC(M36_mC) + H2O	-117.8	-115.6	-116.9

Table S6. Absolute and relative energies of the isomers of BP and reaction coordinate energy profile for the addition

						T + OH + O2 => BP + w				TR + O2 => BP			
**Benzylperoxo radical (BP)**		E	E+ZPE	H	G	ΔE	Δ(E+ZPE)	ΔH(298)	ΔG(298)	ΔE	Δ(E+ZPE)	ΔH(298)	ΔG(298)
CBS-QB3		-420.701247	-420.578069	-420.569298	-420.612375	-54.6	-50.7	-50.9	-42.0	-26,26	-22,20	-22,97	-12,56
G4		-421.172154	-421.049464	-421.040684	-421.083681	-54.8	-50.9	-51.2	-42.1	-26,64	-22,67	-23,44	-12,97
M06	6-31+G(d,p)	-421.005490	-420.881041	-420.872348	-420.915119	-54.9	-50.8	-51.6	-40.7	-25,55	-21,37	-22,18	-11,58
	6-311++G(3df,2pd)	-421.117450	-420.993355	-420.984682	-421.027347	-54.9	-50.8	-51.6	-40.8	-24,26	-20,10	-20,91	-10,28
	cc-pVQZ	-421.160856	-421.035956	-421.027332	-421.069882	-51.1	-47.0	-47.9	-36.3	-22,16	-17,99	-18,80	-8,15
								Murakami CBS-QB3			-22.3		

						T + OH + O2 => BP + w				TR + O2 => BP			
**I-Benzyl peroxy radical (iBO2)**		E	E+ZPE	H	G	ΔE	Δ(E+ZPE)	ΔH(298)	ΔG(298)	ΔE	Δ(E+ZPE)	ΔH(298)	ΔG(298)
CBS-QB3		-420.699102	-420.575965	-420.567184	-420.610337	-53.2	-49.4	-49.6	-40.7	-24,92	-20,88	-21,64	-11,28
G4		-421.132401	-421.015387	-421.004521	-421.056798	-29.8	-29.5	-28.5	-25.2	-1,70	-1,29	-0,75	3,90
M06	6-31+G(d,p)	-420.884102	-420.767177	-420.757304	-420.801489	21.3	20.7	20.6	30.7	50,62	50,08	50,01	59,73
	6-311++G(3df,2pd)	-420.995260	-420.878627	-420.868782	-420.912892	21.8	21.2	21.1	31.0	52,42	51,89	51,81	61,54
	cc-pVQZ	-421.037671	-420.920470	-420.910649	-420.954739	26.2	25.5	25.4	36.0	55,14	54,48	54,42	64,10

						T + OH + O2 => BP + w				TR + O2 => BP			
**o-Bezyl peroxy radical (oBO2)**		E	E+ZPE	H	G	ΔE	Δ(E+ZPE)	ΔH(298)	ΔG(298)	ΔE	Δ(E+ZPE)	ΔH(298)	ΔG(298)
CBS-QB3		-420.652264	-420.531107	-420.522009	-420.565247	-23.9	-21.2	-21.3	-12.4	4,47	7,26	6,70	17,01
G4		-421.126184	-421.005357	-420.996243	-421.039498	-25.9	-23.2	-23.3	-14.4	2,20	5,01	4,45	14,76
M06	6-31+G(d,p)	-420.956120	-420.833491	-420.824506	-420.867423	-23.9	-20.9	-21.6	-10.7	5,43	8,47	7,84	18,35
	6-311++G(3df,2pd)	-421.068512	-420.946102	-420.937142	-420.980024	-24.2	-21.1	-21.8	-11.1	6,45	9,55	8,92	19,41
	cc-pVQZ	-421.111390	-420.988401	-420.979463	-421.022323	-20.0	-17.1	-17.8	-6.4	8,88	11,85	11,24	21,69

						T + OH + O2 => BP + w				TR + O2 => BP			
**m-Bezyl peroxy radical (mBO2)**		E	E+ZPE	H	G	ΔE	Δ(E+ZPE)	ΔH(298)	ΔG(298)	ΔE	Δ(E+ZPE)	ΔH(298)	ΔG(298)
CBS-QB3		-420.594166	-420.477032	-420.477032	-420.521539	12.6	12.7	7.0	15.0	44,59	44,98	38,71	48,23
G4		-421.066816	-420.949901	-420.940324	-420.984536	11.3	11.6	11.8	20.1	-41,35	-40,94	-41,21	-31,50
M06	6-31+G(d,p)	-420.910455	-420.791594	-420.782283	-420.825628	4.8	5.4	4.9	15.5	34,09	34,76	34,34	44,58
	6-311++G(3df,2pd)	-421.021582	-420.902977	-420.893709	-420.936930	5.3	5.9	5.5	15.9	35,90	36,61	36,17	46,45
	cc-pVQZ												

						T + OH + O2 => BP + w				TR + O2 => BP			
**p-Bezyl peroxy radical (pBO2)**		E	E+ZPE	H	G	ΔE	Δ(E+ZPE)	ΔH(298)	ΔG(298)	ΔE	Δ(E+ZPE)	ΔH(298)	ΔG(298)
CBS-QB3		-420.657744	-420.536362	-420.527346	-420.570340	-27.3	-24.5	-24.6	-15.6	1,03	3,97	3,35	13,82
G4		-421.130999	-421.009946	-421.000912	-421.043925	-29.0	-26.1	-26.2	-17.1	-0,82	2,13	1,52	11,98
M06	6-31+G(d,p)	-420.961030	-420.838225	-420.829308	-420.871945	-27.0	-23.9	-24.6	-13.6	2,35	5,50	4,83	15,51
	6-311++G(3df,2pd)	-421.074017	-420.951360	-420.942497	-420.984977	-27.6	-24.4	-25.2	-14.2	2,99	6,25	5,56	16,30
	cc-pVQZ	-421.116993	-420.993695	-420.984864	-421.027269	-23.5	-20.5	-21.2	-9.5	5,36	8,53	7,85	18,59

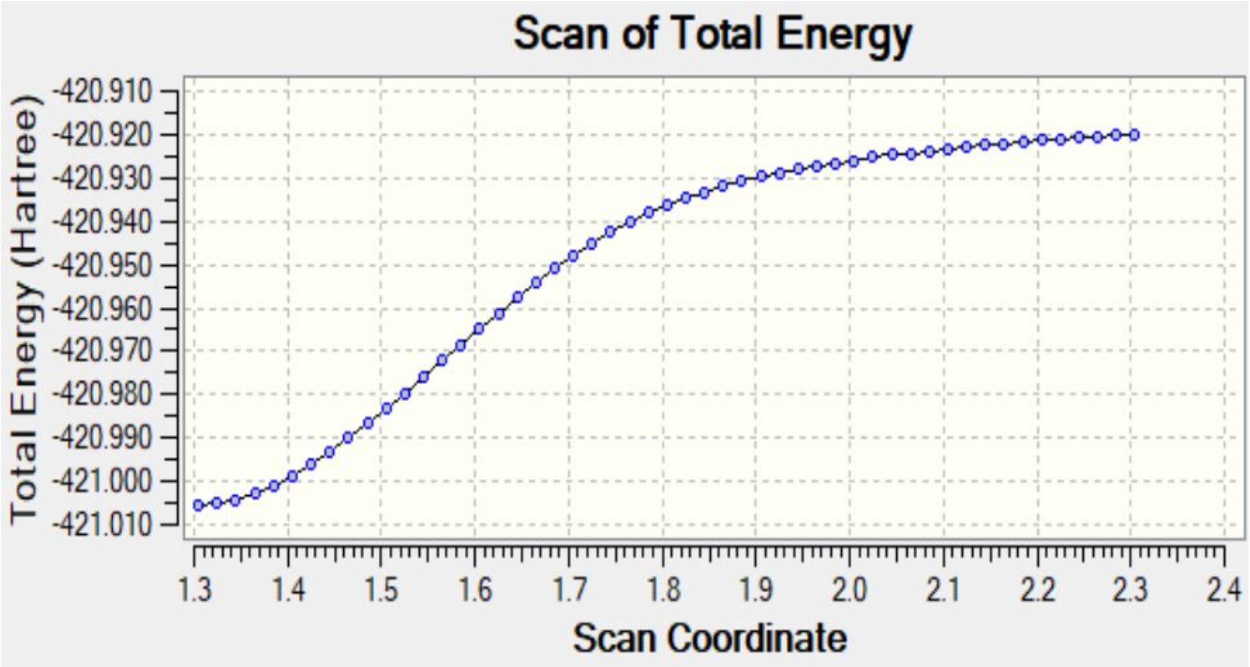

Figure S2. Energy profile for the addition of O_2 to TR. No transition state was found at any of the theoretical levels used in this work. Scan coordinate is the C-O distance in Å.

Full reference of Gaussian 16 (ref. (45) in the paper)

(45) Gaussian 16, Revision B.01, Frisch, M. J.; Trucks, G. W.; Schlegel, H. B.; Scuseria, G. E.; Robb, M. A.; Cheeseman, J. R.; Scalmani, G.; Barone, V.; Petersson, G. A.; Nakatsuji, H.; Li, X.; Caricato, M.; Marenich, A. V.; Bloino, J.; Janesko, B. G.; Gomperts, R.; Mennucci, B.; Hratchian, H. P.; Ortiz, J. V.; Izmaylov, A. F.; Sonnenberg, J. L.; Williams-Young, D.; Ding, F.; Lipparini, F.; Egidi, F.; Goings, J.; Peng, B.; Petrone, A.; Henderson, T.; Ranasinghe, D.; Zakrzewski, V. G.; Gao, J.; Rega, N.; Zheng, G.; Liang, W.; Hada, M.; Ehara, M.; Toyota, K.; Fukuda, R.; Hasegawa, J.; Ishida, M.; Nakajima, T.; Honda, Y.; Kitao, O.; Nakai, H.; Vreven, T.; Throssell, K.; Montgomery, J. A., Jr.; Peralta, J. E.; Ogliaro, F.; Bearpark, M. J.; Heyd, J. J.; Brothers, E. N.; Kudin, K. N.; Staroverov, V. N.; Keith, T. A.; Kobayashi, R.; Normand, J.; Raghavachari, K.; Rendell, A. P.; Burant, J. C.; Iyengar, S. S.; Tomasi, J.; Cossi, M.; Millam, J. M.; Klene, M.; Adamo, C.; Cammi, R.; Ochterski, J. W.; Martin, R. L.; Morokuma, K.; Farkas, O.; Foresman, J. B.; Fox, D. J. Gaussian, Inc., Wallingford CT, 2016.
